# Genome-Wide Characterization and Comparative Analyses of Simple Sequence Repeats among Four Miniature Pig Breeds

**DOI:** 10.3390/ani10101792

**Published:** 2020-10-02

**Authors:** Hongyang Wang, Yang Fu, Peng Gu, Yingying Zhang, Weilong Tu, Zhe Chao, Huali Wu, Jianguo Cao, Xiang Zhou, Bang Liu, Jennifer J. Michal, Chun Fan, Yongsong Tan

**Affiliations:** 1 Institute of Animal Husbandry and Veterinary Science, Shanghai Academy of Agricultural Sciences, Shanghai 201106, China; wanghongyang@saas.sh.cn (H.W.); zhangyingying@saas.sh.cn (Y.Z.); tuweilong@saas.sh.cn (W.T.); wuhuali@saas.sh.cn (H.W.); caojianguo@saas.sh.cn (J.C.); 2 Shanghai Engineering Research Center of Breeding Pig, Shanghai 201302, China; 3 Research Institute of Edible Fungi, Shanghai Academy of Agricultural Sciences, Shanghai 201403, China; fuyang@saas.sh.cn; 4 Institute of Comparative Medicine & Laboratory Animal Management Center, Southern Medical University, Guangzhou 510515, China; kupper@smu.edu.cn; 5 Institute of Animal Science and Veterinary Medicine, Hainan Academy of Agricultural Sciences, Haikou 571100, China; zhanggavin@webmail.hzau.edu.cn; 6 Key Laboratory of Agricultural Animal Genetics, Breeding, and Reproduction of Ministry of Education, Huazhong Agricultural University, Wuhan 430070, China; zhouxiang@mail.hzau.edu.cn (X.Z.); liubang@mail.hzau.edu.cn (B.L.); 7 Department of Animal Sciences, Washington State University, Pullman, WA 99164, USA; jennifer_michal@wsu.edu; 8 Shanghai Laboratory Animal Research Center, Shanghai 201203, China; fanchun@slarc.org.cn

**Keywords:** SSR, repeat unit, length polymorphism, miniature pig, molecular marker

## Abstract

**Simple Summary:**

Simple sequence repeats (SSRs) are present at high densities in regulatory elements, suggesting that they may affect gene function and phenotypic traits. Therefore, SSRs can be exploited in marker-assisted selection. In addition, they can be widely used as molecular markers to study genetic diversity, population structure, and evolution. While SSRs have been widely studied in many mammalian species, very little research has focused on genome-wide SSRs of miniature pigs, a small but special group of pigs that express the dwarf phenotype. Based on the SSR-enriched library building and sequencing, about 30,000 novel polymorphic SSRs for four miniature pig breeds were mapped to the Duroc pig reference genome. The four miniature pig breeds had different numbers and types of SSRs and distributions of repeat units. There were 2518 polymorphic SSRs in the intron or exon regions that were common to all four breeds and functional analyses revealed 17 genes that were associated with body size and other genes that were associated with growth and development. In conclusion, the SSRs detected in the miniature pigs in this study may provide useful genetic markers for the selection of farm animals and the polymorphic SSRs provide valuable insights into the determination of mature body size, as well as the immunity, growth and development of animals.

**Abstract:**

Simple sequence repeats (SSRs) are commonly used as molecular markers in research on genetic diversity and discrimination among taxa or breeds because polymorphisms in these regions contribute to gene function and phenotypically important traits. In this study, we investigated genome-wide characteristics, repeat units, and polymorphisms of SSRs using sequencing data from SSR-enriched libraries created from Wuzhishan (WZS), Bama (BM), inbred Luchuan (LC) and Zangxiang (ZX) miniature pig breeds. The numbers and types of SSRs, distributions of repeat units and polymorphic SSRs varied among the four breeds. Compared to the Duroc pig reference genome, 2518 polymorphic SSRs were unique and common to all four breeds and functional annotation revealed that they may affect the coding and regulatory regions of genes. Several examples, such as *FGF23*, *MYF6*, *IGF1R*, and *LEPROT*, are associated with growth and development in pigs. Three of the polymorphic SSRs were selected to confirm the polymorphism and the corresponding alleles through fluorescence polymerase chain reaction (PCR) and capillary electrophoresis. Together, this study provides useful insights into the discovery, characteristics and distribution of SSRs in four pig breeds. The polymorphic SSRs, especially those common and unique to all four pig breeds, might affect associated genes and play important roles in growth and development.

## 1. Introduction

Simple sequence repeats (SSRs), also known as microsatellites or short tandem repeats (STRs), consist of 2 to 6 base-pair motifs repeated several times in tandem. As a consequence of their wide distribution and high mutation rate in eukaryotic genomes [1], SSRs have been used in genetic diversity and population structure studies [2,3,4,5], for discrimination among species or breeds [6,7], in marker-assisted selection [8,9,10] and in evolution analysis [11]. In humans, SSRs were predicted to be bound by protein-coding transcripts, long non-coding RNAs (lncRNAs) and circular RNAs (circRNAs) and affect competing endogenous RNA crosstalk [12]. Besides being an important category of regulatory elements, polymorphic SSRs could quantitatively regulate the transcription of tissue-specific genes in the development of the frog embryo [13]. Another study showed that polymorphic SSRs play an important role in shaping splicing regulatory elements and lead to alternative splicing events in different stress environments [14]. Over the past decade, an increasing number of studies on SSR discovery and functional analysis have been conducted, providing evidence for the importance of SSRs in gene function and complex traits [15].

Considering their widely functional role, SSRs have been discovered in various taxonomies, most of which discoveries were based on reference genome scanning. However, a poorly assembled genome leads to imperfect SSRs with inaccurate repeat units or repeat number, leading to the limited use of SSRs. For that reason, a high-throughput SSR isolation method based on SSR-enriched library building and next-generation sequencing (NGS) was developed. The major probes designed to enrich the SSR sequences were validated on 13 species, resulting in the acquisition of high-quality genetic markers [16]. Until now, the method has been utilized to isolate SSRs in humans, plants, fungi, invertebrates, and birds. Although SSRs started to be used as markers for breeding projects and genetic diversity studies in pigs in the mid-1990s [17], accurate and genome-wide SSRs are lacking. To our knowledge, only one study isolated polymorphic SSRs from pooled pig breeds based on a porcine reference genome and genome resequencing data [18].

The miniature pig is considered the best model organism for the study of growth and development of animals with small body size. For instance, Wuzhishan pigs (WZS), the most famous indigenous miniature pig, are characterized by their small adult size with mature body weights of only 30 kg [19]. Some genetic mechanisms associated with poor body growth and immunity-related genes were discovered based on transcriptome analyses of liver and muscle tissues of Jeju Native and miniature pigs [20]. The miniature pig shares many anatomical and physiological features with humans and has been used as an animal model in biomedical research, resulting in great contributions to the medical advances of human beings [21]. Recently, studies on chronic renal failure [22], progressive hearing loss [23], and diabetes [24] were conducted on Bama pigs. Of the Chinese indigenous miniature pig breeds, the genome of the Wuzhishan pig was the first to be assembled at the scaffold level [19]. This breed has been widely used for research on metabolic disease [25], diphyodont and craniofacial development [26], mesenchymal stem cells [27] and corneal xenotransplantation [28]. Even so, accurate sequences of genome wide SSRs of miniature pig breeds are not currently available. 

This study was aimed to discover genomic SSRs of the Wuzhishan, Bama (BM), inbred Luchuan (LC) and Zangxiang (ZX) miniature pig breeds based on an SSR-enrichment library. The distribution and functional annotation of SSRs were also compared among the four pig breeds. All the results provided molecular markers for conservation and utilization of germplasm resources of the miniature pig.

## 2. Materials and Methods 

### 2.1. Ethics Statement

These experiments were carried out in accordance with local guidelines for the care of laboratory animals and were approved by the institution’s ethics committee for research using laboratory animals, approval code: SN-XS-20190143.

### 2.2. Animals

Fifteen male pigs with distant relationships from each of four miniature pig breeds (*n* = 60) were involved in this study. Wuzhishan (WZS) pigs were obtained from Hainan Academy of Agricultural Sciences and Zangxiang (ZX) pigs were obtained from Southern Medical University, while Bama (BM) and inbred Luchuan (LC) pigs were obtained from Shanghai Academy of Agricultural Sciences. The inbred Luchuan pigs have been inbred since the 2000s. The smallest boars and gilts, with shorter body lengths than non-inbred Luchuan pigs, were selected for breeding over the past 20 years. The four pig breeds have no relationship with each other and are mainly raised in extensive or semi-extensive farming systems. Ear tissues were collected from the 60 male piglets when they were weaned at 50 days and weighed 2.5~4.0 kg. Tissues were placed in tubes containing 75% ethanol and taken back to the laboratory where they were stored at −80 ℃ for subsequent DNA extraction.

### 2.3. Dna Extraction and Sequencing Based on Simple Sequence Repeat (SSR)-Enriched Library

Genomic DNA was isolated from all samples using a DNeasy Blood and Tissue kit (Qiagen, Hilden, Germany) according to the manufacturer’s instructions. The quantity and quality of the extracted DNA were assessed using a NanoDrop spectrophotometer (Thermo Scientific, Wilmington, DE, USA) and agarose gel electrophoresis (1%), respectively. For each breed, equal amounts of DNA from the 15 pigs were pooled and used for the SSR-enriched library preparation. The protocol of the SSR-enriched library building was similar to that of a previous study [29]. In short, the pooled genomic DNA was digested to small fragments and a standard genomic library was built with a 400-bp insert size. Next, eight biotin-labeled oligonucleotides were used to hybridize SSR repeat sequences in the genomic library and the resulting four libraries from the four pig breeds were sequenced on an Illumina MiSeq platform at Shanghai Personal Biotechnology Co., Ltd. (Shanghai, China). The eight probes which have been described in a previous study were designed to enrich sequences with the following motifs: (AG)_10_, (AC)_10_, (AAC)_8_, (ACG)_8_, (AAG)_8_, (AGG)_8_, (ACAT)_6_ and (ATCT)_6_ [16]. The raw sequence data in fastq format is deposited in the Sequence Read Archive (SRA) and the reviewer link is https://dataview.ncbi.nlm.nih.gov/object/PRJNA628105?reviewer5mfadppoucgn41cpgj2idujl86.

### 2.4. Data Treatment and SSRs Scanning

For each library, paired-end data (2 × 250 bp) were generated from the sequencing platform in fastq format. AdapterRemoval software (v2.1.7) [30] was used to remove adapters and low-quality reads. First, the Q value of the base pair (bp) was scanned with 5 bp sliding window and 1 bp sliding step for all reads. In a window, if the average Q value was less than 20 or the Q value of the last base pair was less than 2, the base pair next to the last and the previous base pair was kept. Second, paired reads were removed if the length of one of the pairs was less than 50 bp. After quality filtering, FLASH software (V1.2.11) [31] was utilized to combine read1 and read2 from each of the paired reads and used to generate longer sequences with the following criteria: (1) Min overlap: 100; (2) Max mismatch density: 0.1; (3) Allow “outie” pairs: false; (4) Cap mismatch quals: false. SSRs were scanned and counted for the pig reference genome (*Sscrofa11.1*, GenBank: GCA_000003025.6) and the combined sequences from each of the four datasets using the MIcroSAtellite (MISA) script [32,33]. In this step, the most important parameter is minimum repeat, which was defined as 6, 5, 5, 5 and 5 for di-, tri-, tetra-, penta-, and hexanucleotides, respectively [34]. Distributions of repeat units for the pig reference genome and four pig breeds were drawn using R software (3.6.1).

### 2.5. Analysis of Polymorphic SSRs and Functional Annotation

Polymorphic SSRs depended on SSR length polymorphism (SSLP) and were discovered from SSR-containing sequences that were obtained from the combined sequences. First of all, an in-house Perl script was utilized to identify and mask SSRs with “R” in the SSR-containing sequences. In this step, if the length of the flanking sequence of SSR was less than 20 bp, the sequence would be removed for the reason that it could not be accurately used for similarity analysis. After that, clustering was performed based on similarity of the flanking sequence using CD-HIT software [35]. Similarity and coverage were 90% and 70%, respectively. Other parameters were defined as 1 for gap and 0 for gep-ext. For the clustering results, another in-house Perl script was used to identify SSLP. If only one type of length existed in a cluster, the corresponding SSLP would be defined as 1. If the length of the SSR had two types, the SSLP of the SSR would be defined as 2, and so on. Finally, we obtained the polymorphic SSRs and SSLP for each type of SSR.

SSRs with an SSLP more than 1 were selected and alignment was performed based on the flanking sequences of SSRs in the corresponding cluster. The flanking sequences longer than 20 bp were extracted and mapped to the reference genome (*Sscrofa11.1*) using Burrows–Wheeler Alignment software [36]. According to chromosome coordinates of the mapped SSRs, overlapping was analyzed to find common and specific SSRs among the four pig breeds using the UpSetR package [37]. SSRs annotation and associated functional genes were discovered using annotated files from the Ensembl database (*Sus_scrofa.Sscrofa11.1.97*). Functional enrichment analysis was performed using the clusterProfiler [38] package and corresponding database (*org.Ss.eg.db, V3.10.0*).

### 2.6. Designing Primers and Experimental Validation

Based on the flanking sequences of SSRs with SSLP more than 1, primer pairs were designed using Primer3 (v2.3.6) [39]. Three primer pairs were chosen to detect alleles of the SSR in all 60 pigs at high resolution using fluorescence polymerase chain reaction (PCR) and capillary electrophoresis. First of all, we checked specific amplification and length of PCR products using normal PCR followed by agarose gel electrophoresis. After that, forward primers were fluorescence-labeled with HEX at the 5′ end as described in a previous study [40]. Fluorescence PCR were performed on ABI-2720 thermal cycle (Applied Biosystems, Foster City, CA, USA) and each 25 μL reaction contained 1 μL of each primer (10 μm), 1μL of template DNA, 2 μL 10 × buffer, 0.5 μL dNTP, 0.5 μL Taq enzyme and 14 μL ddH2O. Cycling conditions were 95 °C for 4 min, followed by 10 cycles with 60 °C for 30 s, 72 °C for 30 s and 95 °C for 30 s, followed by 25 cycles with 52 °C for 30 s, 72 °C for 30 s and 72 °C for 7 min. The final amplicons were subjected to capillary electrophoresis (ABI-3730XL, Applied Biosystems, Foster City, CA, USA) and the output data was analyzed by GeneMapper software (V2.2.0).

## 3. Results

### 3.1. Overview of SSRs and Repeat Units in the Pig Reference Genome

**Figure 1:**
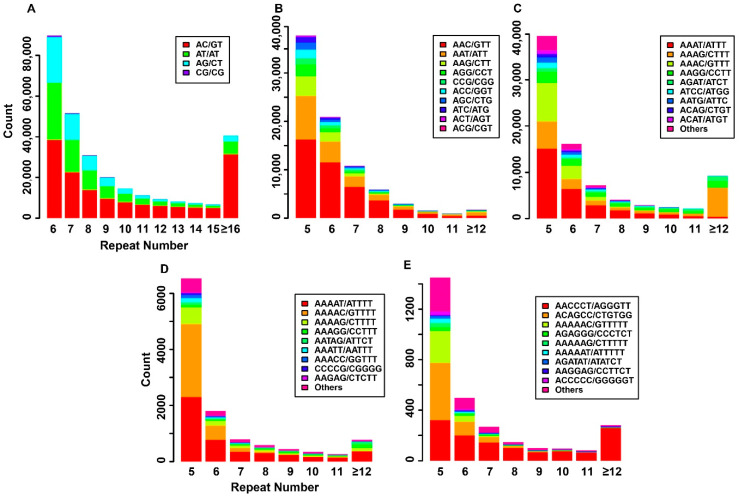
Distributions of repeat units for different simple sequence repeat (SSR) types in the pig reference genome. (**A**–**E**) The distributions of repeat units in dinucleotide repeat (Di-SSR), trinucleotide repeat (Tri-SSR), tetranucleotide repeat (Tetra-SSR), pentanucleotide repeat (Penta-SSR) and hexanucleotide repeat (Hexa-SSR). The X axis represents repeat number, Y axis represents the count of the repeat unit corresponding to different colors.

Using MISA software and the parameters described above, we scanned the pig reference genome (*Sscrofa11.1*) to discover the profiles of the SSRs and repeat units in the pig. We discovered a total of 471,287 SSRs, including 290,373 dinucleotide repeats (Di-SSRs), 82,517 trinucleotide repeats (Tri-SSRs), 83,936 tetranucleotide repeats (Tetra-SSRs), 11,545 pentanucleotide repeats (Penta-SSRs) and 2916 hexanucleotide repeats (Hexa-SSRs). All the SSRs occupied 0.4% of the reference genome (Table S1). The size and proportion of the repeat units were also discovered in different types of SSR. Among Di-SSRs, AC/GT (52.2%) was the most common repeat unit and CG/CG (0.5%) was the least abundant repeat unit. For Tri-SSRs, the most abundant type was AAC/GTT (50.2%), followed by 22.7% and 9.1% for AAT/ATT and AAG/CTT, respectively. In Tetra-SSRs, AAAT/ATTT (34.6%), AAAG/CTTT (21.1%) and AAAC/GTTT (15.0%) were the three major types and occupied 70.7% of all the tetranucleotide repeat units. A/T-rich motifs were the main types in Penta-SSRs, which was similar to the tetranucleotide repeats. However, the most abundant repeat unit for Hexa-SSRs was AACCCT/AGGGTT (42.2%), followed by ACAGCC/CTGTGG (21.4%) (Figure 1).

### 3.2. SRR Discovery from Four Miniature Pig Breeds

A total of 60.6 million raw reads were obtained from four datasets. 54.7 million (90.3%) reads with an average length of 232 bp were left after quality filtering using AdapterRemoval software (v2.1.7). According to the overlapped and mismatched reads, we combined a total of 47.9 million (87.6%) reads utilizing FLASH software (V1.2.11), and obtained 6.6, 4.3, 3.8 and 9.0 million combined sequences for Wuzhishan (WZS), Bama (BM), inbred Luchuan (LC) and Zangxiang (ZX) pigs, respectively (Table 1). In all four datasets, we found the length of the sequences ranged from 100 bp to 500 bp and sequences with 300 bp in length were the most abundant (Figure S1). The raw SSRs data generated from MISA software is displayed in File S1, which showed that the number of SSRs was greatest in WZS, followed by BM and LC, while ZX had the least number of SSRs. In the four pig breeds, Di-SSRs were far more frequent (75.7%, 70.5%, 75.5% and 51.5% for WZS, BM, LC and ZX, respectively) than other SSR types, followed by Tri- and Tetra-SSRs, which is similar to the proportion of different SSR types in the reference genome as described above (Table 1). 

### 3.3. Frequency of Repeat Units

For each type of SSR, the frequency of the repeat units at the position of combined sequences was checked and most of the repeat units were located in proximal sequences in all datasets (Figure S2). Furthermore, the distributions of the number of repeat units were calculated in each type of SSR (Table S2) and a comparison was performed between the four miniature pig breeds and the reference genome. In the four pig breeds, AC/GT and AAC/GTT repeats, for Di- and Tri-SSRs, respectively, were more common than others in the corresponding SSR type, which was similar to trends observed in the reference genome. However, in the four pig breeds, AGAT/ATCT, AATAG/ATTCT and AAGGAG/CCTTCT were the most abundant repeat units for Tetra-, Penta- and Hexa-SSRs, respectively, which were different than the distributions of repeat units in the reference genome.

**Figure 2:**
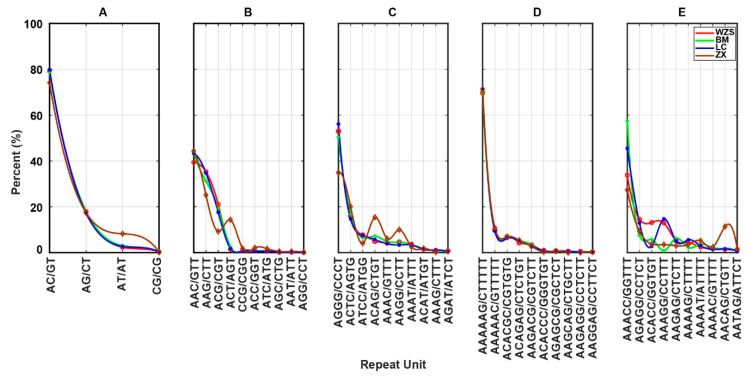
Distribution models of different repeat units among four miniature pig breeds. (**A**–**E**) Reading from left to right are the distributions of repeat units in Di-SSR, Tri-SSR, Tetra-SSR, Penta-SSR and Hexa-SSR, respectively. The X axis represents types of repeat unit, Y axis represents percentage of repeat units in corresponding total SSRs. The red, green, blue and brown dots connected by lines represent WZS, BM, LC and ZX, respectively.

We selected all the repeat units for Di- and Tri-SSRs and the top 10 repeat units for Tetra-, Penta- and Hexa-SSRs and compared the proportion based distribution models among the four miniature pig breeds (Figure 2). There were no differences in Di- and Penta-SSRs among the four pig breeds, which showed a similar distribution. Special distributions were found in the ZX pig, which showed a high abundance of ACT/AGT in Tri-SSRs and ACAG/CTGT and AAGG/CCTT in Tetra-SSRs. In comparison, the other three pig breeds had similar distribution models. There were extremely diverse distributions of repeat units of Hexa-SSR among the four pig breeds.

### 3.4. Polymorphic and Functional SSRs in Four Miniature Pig Breeds

**Figure 3:**
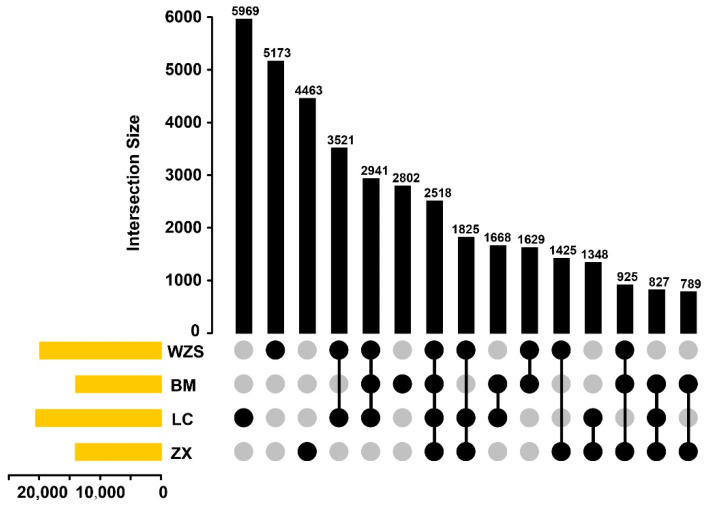
Overlapping analysis of SSRs among four pig breeds. The horizontal frames (in yellow) represent the total number of mapped SSRs with an SSLP more than 1 corresponding to the four pig breeds. The vertical frames (in black) show the SSR number corresponding to the bottom intersection groups.

We discovered SSR length polymorphisms (SSLPs) in all SSRs examined. A summary of the total clusters and corresponding SSLP is displayed in Table 2 and the details are shown in File S2. We focused on 60,020, 70,886, 63,968 and 42,400 clusters containing SSRs with SSLP more than 1 for the WZS, BM, LC and ZX pig breeds, respectively (Table 2). Among them, 19,957, 14,099, 20,671 and 14,120 clusters containing 26,393, 17,722, 28,387 and 16,886 SSRs, respectively, were mapped to the reference genome. The details of total SSRs with SSLP and mapped clusters are shown in File S3. According to the results of the overlapping analysis among the four pig breeds, 5173, 2802, 5969 and 4463 clusters were specific for the WZS, BM, LC and ZX pig breeds, respectively, and 2518 clusters were common among all four pig breeds (Figure 3).

**Figure 4:**
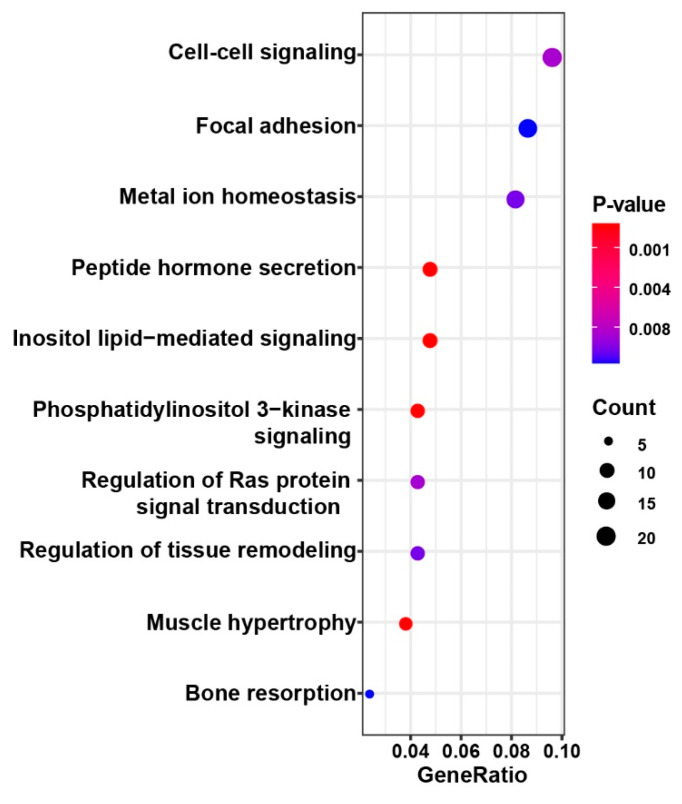
Enrichment analysis of functional genes affected by polymorphic SSRs.

We merged the 2518 common clusters to annotate and ascertain the universal functions of the SSRs in the four pig breeds. Results showed that most were located in intergenic regions (63.0~65.4%) and 80, 357 and 436 clusters overlapped with 5’ untranslated region (5′ UTR), 3’ untranslated region (3′ UTR) and the coding sequence (CDS), respectively. The results illustrate that polymorphic SSRs were commonly found in noncoding regions and the rest of the SSRs were located in exons, which might affect the function of associated genes. For the SSRs located in the exons, functional enrichment analysis of associated genes was conducted and we found most of the genes were involved in cell–cell signaling, peptide hormone secretion and other biological processes with *p*-value less than 0.01 (Figure 4). Finally, we identified the functional genes corresponding to the polymorphic SSRs with repeat units. Most of these genes were associated with bone remodeling, muscle development and immunity and are described in Table 3 and Table S3. 

### 3.5. Experiment Validation Using Fluorescence Polymerase Chain Reaction (PCR) and Capillary Electrophoresis

**Figure 5:**
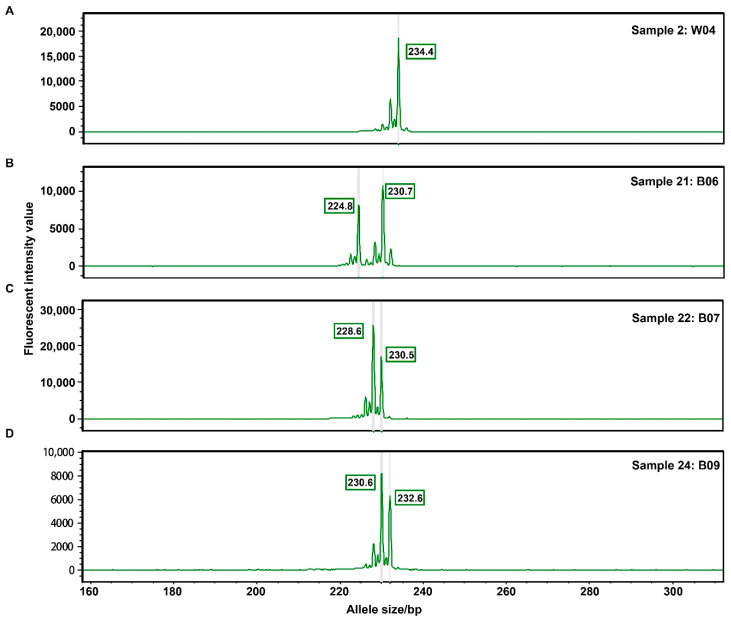
Different alleles of SSR located in chr1:272,578,714-272,578,954. The output data from capillary electrophoresis was analyzed by GeneMapper software (V2.2.0) and generated the allele report for 60 pigs. (**A**–**D**) Five alleles 224, 228, 230, 232 and 234 bp in length existed in chr1:272,578,714-272,578,954 bp are exampled in corresponding pig individuals. The X axis represents allele size, Y axis represents fluorescent intensity for different allele size, one or two peaks on the green line represents homozygosity and heterozygosity, respectively. The sample number W04 means the individual in the Wuzhishan pig breed. The sample number B06, B07 and B09 mean the individuals in Bama pig breed.

Three primers were selected to detect polymorphic SSRs in the 60 pigs to confirm the sequencing results (Table 4). For the first locus, our predicted result showed that five variations located in the region ranged from 272,578,714 bp to 272,578,954 bp of chromosome 1, and the corresponding SSRs consisted of an (AC) repeat unit ranging from 12 to 17 repeats. Capillary electrophoresis analysis of PCR amplicons confirmed that five alleles (except for one rare allele with 226 bp in length) 224, 228, 230, 232 and 234 bp in length existed in all four pig breeds (Figure 5). We verified that six alleles occurred in each of two other loci, which was confirmed with the polymorphic SSRs (Figures S3 and S4). The raw data from capillary electrophoresis and alleles are displayed in Table S4.

## 4. Discussion

Because of the rapid development of NGS, SSRs have been discovered through scanning the reference genome and genotyping based on a large set of genome resequencing data in pigs. Here, for the first time, an SSR-enriched library was built, sequenced and analyzed to describe characteristics of SSRs in four miniature pig breeds, including different types, distribution of repeat units, polymorphism and function, providing accurate genetic markers for pig breeding and polymorphic SSRs for gene function analysis.

Based on the SSR-enriched library, we obtained an average of 1,225,072 SSRs in the four pig breeds, which is less than the number of SSRs in the MicroSatellite DataBase (MSDB) [54]. In addition, Hexa- and Tetra-SSR were the most abundant types in the MSDB (56%) and another previous study (31.3%) [18], respectively. However, Di-SSRs were far more frequent than other SSR types and similar trends were found for the reference genome in our study. The difference between our study and previous results is probably because of the minimum repeat size used for the SSR scanning. The most commonly used methods for SSR scanning contain MISA, Tandem Repeats Finder [55] and other custom scripts [56] based on Python or Perl, which are based on similar principles in terms of minimum repeat size. The two studies defined minimum repeats as 6, 4, 3, 3, 3 (or 2) for di-, tri-, tetra-, penta-, and hexanucleotides, respectively. In the present, the minimum repeat size was set to 5 for repeat units longer than 2, the same as other studies [34,57,58,59], which led to a smaller number of Hexa- and Tetra-SSR. Moreover, previous studies report that mononucleotide repeats are most frequent in eukaryotes, followed by dinucleotide repeats, while trinucleotide repeats are more abundant in prokaryotic genomes [60,61,62].

Consistent with previous results [18], AC/GT and AAC/GTT were the most abundant repeat units in the pig for Di- and Tri-SSR, respectively. In contrast, GC-containing SSR, such as CG/GC and ACG/CGT, accounted for a small percentage in the reference genome and the four pig breeds, which is similar to reports in other species [63,64,65,66]. The bias against GC sequences in the process of library building and sequencing might explain why the GC-SSRs were relatively rare, however, eight probes which contained (ACG)_8_ and (AGG)_8_ were used to hybridize the GC-containing SSR in this study and should have ensured comprehensive genome-wide SSR enrichment. Therefore, GC-containing SSRs are infrequent and have fewer polymorphisms, explaining why the GC enrichment sequence is always associated with functionality [67]. Furthermore, AGAT/ATCT, AATAG/ATTCT and AAGGAG/CCTTCT were the most abundant repeat units in the four pig breeds for Tetra-, Penta- and Hexa-SSRs, respectively, which was different from the reference genome and due to the fact that the repeat units used for enrichment were over-represented in these SSRs, in particular AAG, AGG and ATCT. Nevertheless, all different types of repeat unit were discovered and displayed different distributions among the four pig breeds.

SSR-based genotyping has been used to study genetic diversity and breed identification within pigs, and most of the studies depending on SSR markers were developed in the domestic pig [17]. However, SSRs and primers developed from different species or breeds always lead to most SSRs with no polymorphism of interest. At the genome-wide level, 1,620,469 SSRs were discovered in the pig reference genome (Duroc) and only 16,527 SSRs displayed high polymorphism in a total of 102 pigs, including 8 Chinese domestic pig breeds and 6 commercial pig breeds [18]. In the current study, 60,020, 70,886, 63,968 and 42,400 SSRs with SSLP more than 1 were discovered for the WZS, BM, LC and ZX pig breeds, respectively, providing genetic markers for further analysis. In addition, frequency analysis of repeat units showed different distributions for Hexa-SSRs among the four pig breeds, and specific distributions of Tri- and Tetra-SSRs in the ZX pigs. We speculated that distribution analysis of repeat units combined with validation of SSR polymorphism on a population scale might accurately discriminate among pig breeds.

Body size is one of the most important traits for the research of growth and development and improving production in farm animals. Based on the wide functions of SSRs in genes and traits described above, we examined polymorphic SSRs in genes associated with body size in four miniature pig breeds. Interestingly, about 17 genes involved in body size are affected by polymorphic SSRs. In humans, mutations in *IGF1*, *SHOX*, *GHRHR*, *ZBTB38* and *PIT1* genes can explain part of height variation. We found two variations of 6 and 8 repeats of the (GCG) repeat unit that affect the 5′ UTR of *IGF1* gene. Polymorphic SSRs were also discovered in the introns of *ZBTB7C*, *ZBTB16* and *ZBTB20*, which belong to the Krueppel C2H2-type zinc-finger protein family. The coding sequence of the *LEPROT* gene is affected by polymorphic SSRs with (CA) repeat units ranging from 18 to 23 in our findings. A previous study confirmed that the *LEPROT* gene was related to the fat content of Duroc pigs [50] and had a role in the leptin receptor which was related to the reduction of body size in domestic fowl [68]. Compared to the 11 genes related to small body size in the Chinese Debao Pony [69], we found six genes belonging to the FACIT (fibril-associated collagens with interrupted triple helices) collagen family, including *COL6A6*, *COL8A1*, *COL25A1*, *COL12A1*, *COL11A1* and *COL14A1*, and other genes such as *FGF14*, *FGF23*, *GDF3*, *BMP10*, *LEMD1* and *PCSK6* that are associated with bone and muscle development also had polymorphic SSRs. For such complex quantitative traits such as body size, the *IGF1* gene contributes to only 16% of height variation in humans and the majority of body size in dogs. The genes and their corresponding contribution to body size still need to be discovered in pigs. However, the polymorphic SSRs and associated genes discovered in this study might provide some useful information that may contribute to future understanding of mature body size in pigs.

## 5. Conclusions

In summary, we built and sequenced an SSR-enrichment library and analyzed SSRs at the genome-wide level. We described unique SSR characteristics among four miniature pig breeds, including frequency of SSR type and distribution models of the repeat units. Polymorphic SSRs that were common to the four pig breeds were discovered and annotated, revealing that functional polymorphic SSRs might be related to the growth and development of the miniature pig through their effects on associated genes. The SSRs discovered from this study supplement the genetic variation information of the pig genome and molecular markers of the miniature pig. The established method might provide a reference for SSR analysis, identification of different species and breeds, and a genome-wide association study based on SSRs in the future.

## Figures and Tables

**Table 1:** Statistics of four datasets and SSRs discovered from four pig breeds.

Items	WZS ^1^	BM ^1^	LC ^1^	ZX ^1^
Raw reads	17,083,436	10,335,212	10,466,408	22,759,784
High quality reads	15,473,282	9,660,700	9,255,072	20,279,502
Total length of high quality reads (base pair, bp)	3,549,570,313	2,367,772,949	2,010,410,274	4,756,156,243
Combined sequences	6,658,339	4,369,729	3,885,823	9,043,156
Total length of combined reads (bp)	2,103,692,216	1,435,035,633	1,177,952,198	2,948,109,147
Dinucleotide repeats (Di-SSRs)	4,127,838	1,843,882	2,216,458	951,588
Trinucleotide repeat (Tri-SSRs)	586,364	264,912	316,662	101,743
Tetranucleotide repeat (Tetra-SSRs)	234,811	125,631	134,298	122,506
Pentanucleotide repeat (Penta-SSRs)	33,676	23,977	18,932	46,849
Hexanucleotide repeat (Hexa-SSRs)	17,693	7088	9198	2382

^1^ WZS, BM, LC and ZX represent Wuzhishan, Bama, inbred Luchuan and Zangxiang pigs.

**Table 2:** SSR length polymorphisms (SSLPs) and corresponding number of SSRs in four pig breeds.

Items ^1^	WZS	BM	LC	ZX
SSR-containing sequences	3,804,507	1,849,907	2,061,668	1,448,491
Total clusters	377,558	398,960	433,945	453,677
SSLP = 1	317,538	328,074	369,977	411,277
SSLP = 2	37,841	44,188	41,098	30,024
SSLP = 3	13,861	16,865	14,879	8542
SSLP = 4	5420	6341	5210	2650
SSLP = 5	1840	2221	1779	747
SSLP = 6	567	704	525	202
SSLP = 7	206	257	182	75
SSLP = 8	88	103	101	39
SSLP = 9	60	61	50	29
SSLP ≥ 10	137	146	144	92

^1^ SSLP means SSR length polymorphism.

**Table 3:** Summary information of functional SSRs (SSLP ≥ 2) and associated genes.

Genomic Coordinate of SSR Flanking Sequence ^1^	Repeat Sequence	Gene	Functional Region ^2^	Gene Function
1:137690845_137690967	(GCG)_6_, (GCG)_8_,	*IGF1R*	5′ UTR	Muscle development [41]
3:23673114_23673194	(GT)_13_, (CA)_15_	*IGSF6*	5′ UTR	Inflammatory disease [42]
3:73527603_73527759	(AC)_9_, (GT)_13_, (GT)_15_, (GT)_17_, (GT)_18_, (GT)_20_	*BMP10*	3′ UTR	Skeletal development [43]
4:93179209_93179533	(TG)_15_, (CA)_16_, (CA)_17_, (TG)_18_, (CA)_22_	*PEAR1*	3′ UTR	Platelet activation [44], myoblast differentiation [45]
4:102696359_102696552	(CA)_22_, (GT)_23_	*SPAG17*	CDS	Sperm motility [46]
5:62835807_62836251	(AC)_12_, (TG)_13_, (AC)_14_, (AC)_16_, (GT)_17_	*GDF3*	CDS	BMP inhibiting [47]
5:66028468_66028506	(GT)_15_, (GT)_16_, (TG)_17_, (GT)_18_	*FGF23*	5′ UTR	Bone remodeling [48]
5:100760758_100761162	(GT)_14_, (AC)_18_	*MYF6*	3′ UTR	Muscle development [49]
6:146979743_146980171	(CA)_18_, (TG)_19_, (CA)_20_, (CA)_22_, (CA)_23_	*LEPROT*	CDS	Fat content [50]
9:136235771_136236041	(AC)_17_, (AC)_19_	*SPATA48*	CDS	Spermatogenesis [51]
13:111022378_111022649	(GT)_15_, (GT)_16_	*TNFSF10*	3′ UTR	Cell apoptosis [52]
14:88520086_88520470	(CA)_15_, (GT)_18_, (CA)_21_, (GT)_22_, (TG)_23_, (TG)_24_, (TG)_25_, (TG)_26_, (TG)_28_	*GDF10*	3′ UTR	TGFβ superfamily [53]

^1^ genomic coordinate corresponding to chromosome: start_end. ^2^ 5’ UTR, 3’UTR and CDS mean 5’ untranslated region, 3’ untranslated region and coding sequence, respectively.

**Table 4:** Information and validation of 3 SSRs in 60 pigs.

Loci ^1^	Primer Sequences (5′–3′) ^2^	Repeat Sequence ^2^	Allele Size/bp ^2^	NO. of Alleles ^3^
chr1: 272,578,714-272,578,954	F: TACACACCACGCGTGTACCT	(AC)_12_, (AC)_14_, (AC)_15_, (AC)_16_, (AC)_17_	207~275	5
	R: ACAACGGTTGCTGCTTTTTC			
chr11:70,376,652-70,376,765	F: CCAGCTTTCCAGTTTCGTGT	(GT)_14_, (GT)_15_, GT)_16_, (GT)_17_, (GT)_18,_ (GT)_19_	77~149	6
	R: AGATTGTGAGTGCGCAGATG			
chr18:1,858,964-1,859,153	F: CTCCAAGGTCAAAAGCCAAA	(AC)_13_, (AC)_14_, (AC)_15_, (AC)_16_, (AC)_17_, (AC)_18_	154~226	6
	R: CGACTTGGGGTTTCCTAACA			

^1^ Based on reference genome. ^2^ Based on SSR-containing sequences. ^3^ Based on the fragment analysis of 60 pigs on an ABI-3730XL.

## Supplementary Materials

The raw sequence data generated from Illumina MiSeq platform are deposited in the Sequence Read Archive (SRA) and the reviewer link is https://dataview.ncbi.nlm.nih.gov/object/PRJNA628105?reviewer=5mfadppoucgn41cpgj2idujl86. The supplementary files were uploaded to Zenodo (10.5281/zenodo.3963831, https://zenodo.org/deposit/3963831) during the manuscript submission process. The following are available online at https://www.mdpi.com/2076-2615/10/10/1792/s1. **File S1**: SSRs discovered from MISA software for four pig breeds, **File S2**: Sequences of total clusters and corresponding predicted polymorphism of SSRs for four pig breeds, **File S3**: Information of the mapped SSRs and corresponding primers designed for four pig breeds. Supplementary figures and tables were uploaded during the manuscript submission process, **Figure S1**: Length distribution of combined sequences in four datasets, **Figure S2**: Start position of repeat units at combined sequences for data of four breeds, **Figure S3**: Different alleles of SSR located in chr11:70,376,652-70,376,765, **Figure S4**: Different alleles of SSR located in chr18:1,858,964-1,859,153, **Table S1**: Summary description of SSRs discovered in pig reference genome (*SScrofa11.1*), **Table S2**: Details of repeat units in each types of SSRs, **Table S3**: Annotation results of common SSRs existed among four pig breeds and functional genes affected by SSRs, **Table S4**: Alleles of three polymorphic SSRs detected in 60 pigs. All data were obtained from at least three independent experiments.
