# Supplementary material for: Genome-Wide Characterization and Comparative Analyses of Simple Sequence Repeats among Four Miniature Pig Breeds
Source: Animals (Basel). 2020 Oct 2;10(10):1792. doi: 10.3390/ani10101792 (PMC7600727; doi:10.3390/ani10101792)
Supplement: Supplementary file 1 [file animals-10-01792-s001.pdf]

Supplementary Material

1 Length distribution of combined sequences

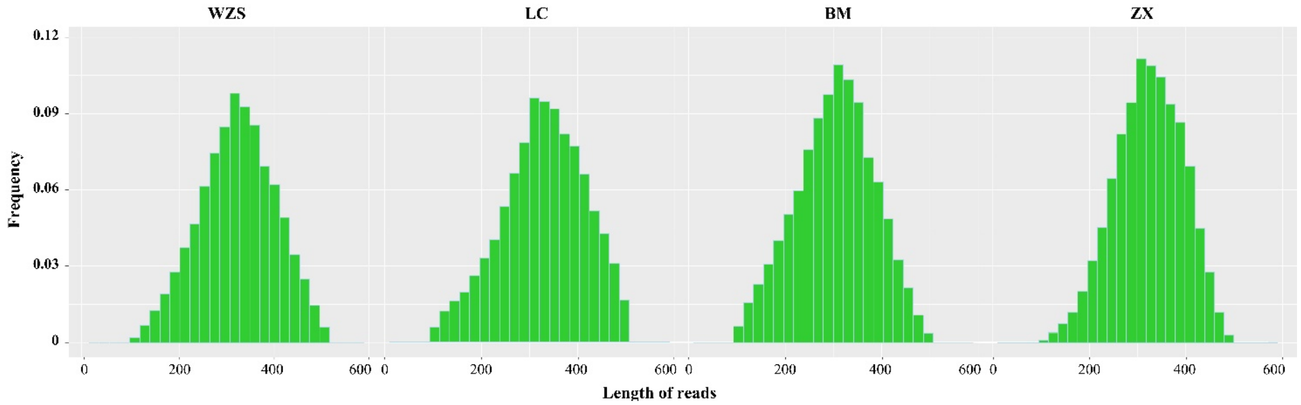

Supplementary Figure S1. Length distribution of combined sequences in four datasets. The X axis represents length of combined sequences, Y axis represents frequency of combined sequence with corresponding length in each dataset. Wuzhishan (WZS), inbred Luchuan (LC), Bama (BM) , and Zangxiang (ZX).

2 Frequency statistics of start position of repeat units at combined sequences

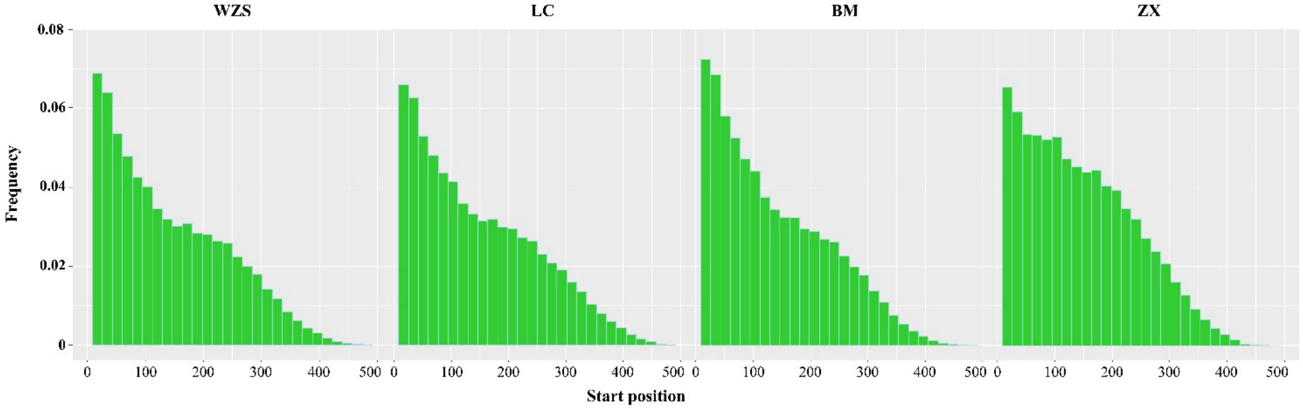

Supplementary Figure S2. Start position of repeat units at combined sequences for data of four breeds. The X axis means position of combined sequences, Y axis means frequency of repeat units at corresponding position in each dataset. Wuzhishan (WZS), inbred Luchuan (LC), Bama (BM) , and Zangxiang (ZX).

3 Alleles of SSR located in chr11:70,376,652-70,376,765

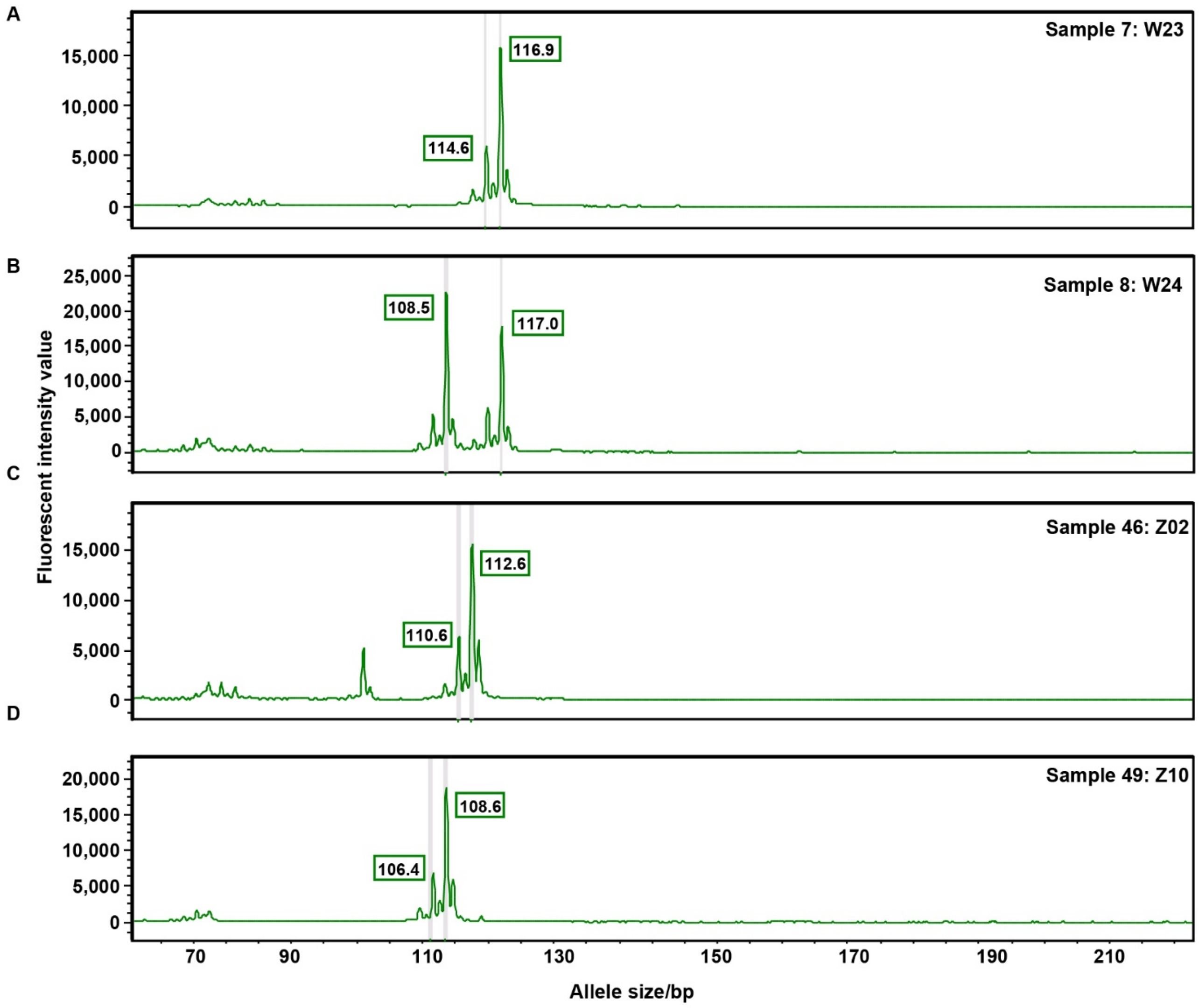

Supplementary Figure S3. Different alleles of SSR located in chr11:70,376,652-70,376,765. The output data from capillary electrophoresis was analyzed by GeneMapper software (V2.2.0) and generated the allele report for 60 pigs. **(A-D)** Six alleles 107, 109, 111, 113, 115 and 117 bp in length existed in chr11:70,376,652-70,376,765 bp are exampled in corresponding pig individuals. The X axis represents allele size, Y axis represents fluorescent intensity for different allele size. The sample number W23 and W24 mean the individuals in Wuzhishan pig breed. The sample number Z02 and Z10 mean the individuals in Zangxiang pig breed.

4 Alleles of SSR located in chr18:1,858,964-1,859,153

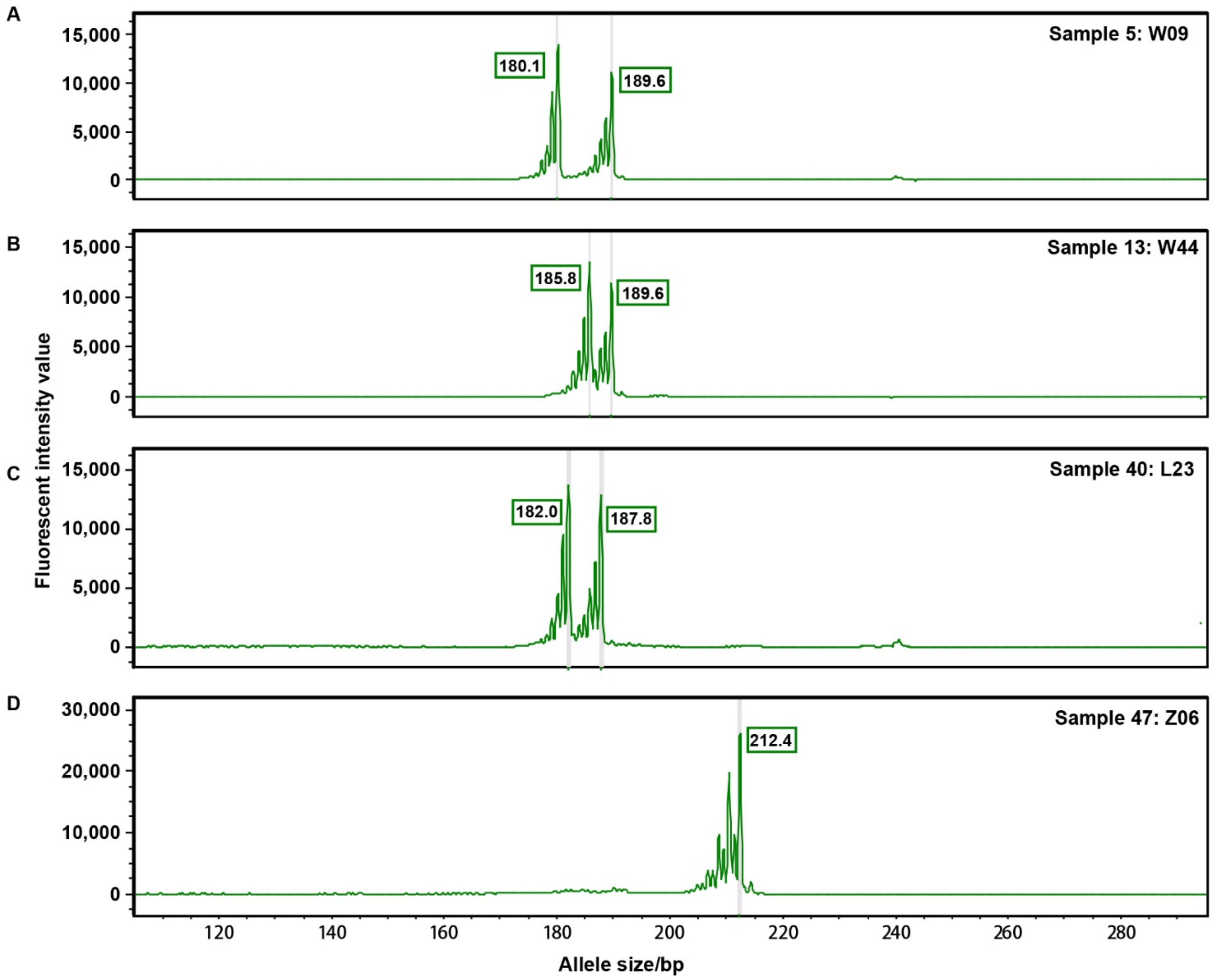

Supplementary Figure S4. Different alleles of SSR located in chr18:1,858,964-1,859,153. The output data from capillary electrophoresis was analyzed by GeneMapper software (V2.2.0) and generated the allele report for 60 pigs. **(A-D)** Six alleles 180, 182, 186, 188, 190 and 212 bp in length existed in chr18:1,858,964-1,859,153 bp are exampled in corresponding pig individuals. The X axis represents allele size, Y axis represents fluorescent intensity for different allele size, one and two peaks on the green line represents homozygosity and heterozygosity, respectively. The sample number W09 and W44 mean the individuals in Wuzhishan pig breed. The sample number L23 and Z06 mean the individual in inbred Luchuan and Zangxiang pig breed, respectively.

5 Statistic of SSRs in pig reference genome (*Sus Scrofa 11.1*)

Types	Counts	Percentage in total SSRs (%)	Length (bp)	Percentage in total length (%)
Di-SSRs	290,373	61.61	5,671,612	0.227
Tri-SSRs	82,517	17.51	1,540,512	0.062
Tetra-SSRs	83,936	17.81	2,394,808	0.096
Penta-SSRs	11,545	2.45	374,295	0.015
Hexa-SSRs	2,916	0.62	130,530	0.005
Total	471,287	100	10,111,757	0.404

Supplementary Table S1. Summary description of SSRs discovered in pig reference genome (*Sus Scrofa 11.1*).

6 Statistics of all repeat units for four pig breeds

Types of SSR	Repeat units	WZS		BM		LC		ZX	
		Number[a]	Percentage[a]	Number[a]	Percentage[a]	Number[a]	Percentage[a]	Number[a]	Percentage[a]
Di-SSR	AC/GT	3,278,190	79.42	1,451,158	78.70	1,769,789	79.85	705,333	74.12
	AG/CT	741,091	17.95	331,201	17.96	380,473	17.17	165,852	17.43
	AT/AT	96,222	2.33	56,327	3.05	60,033	2.71	78,414	8.24
	CG/CG	12,335	0.30	5,196	0.28	6,163	0.28	1,989	0.21
Tri-SSR	AAC/GTT	230,945	39.39	115,792	43.71	137,176	43.32	45,173	44.40
	AAG/CTT	206,986	35.30	82,705	31.22	110,024	34.74	25,537	25.10
	AGG/CCT	123,547	21.07	51,703	19.52	55,931	17.66	9,399	9.24
	AAT/ATT	8,935	1.52	6,665	2.52	5,502	1.74	14,611	14.36
	AGC/CTG	4,864	0.83	2,195	0.83	2,403	0.76	1,810	1.78
	ATC/ATG	4,428	0.76	2,118	0.80	2,067	0.65	2,169	2.13
	ACC/GGT	3,801	0.65	2,018	0.76	2,164	0.68	1,764	1.73
	CCG/CGG	1,099	0.19	741	0.28	606	0.19	548	0.54
	ACT/AGT	1,117	0.19	653	0.25	514	0.16	526	0.52
	ACG/CGT	642	0.11	322	0.12	275	0.09	206	0.20
Tetra-SSR	AGAT/ATCT	124353	52.96	63380	50.45	75397	56.14	42796	34.93
	AAAG/CTTT	37531	15.98	21064	16.77	19795	14.74	24636	20.11
	ACAT/ATGT	18103	7.71	8185	6.52	10060	7.49	4960	4.05
	AAAT/ATTT	11634	4.95	9024	7.18	7653	5.70	18901	15.43

	AAAC/GTTT	10858	4.62	5689	4.53	4502	3.35	12258	10.01
	AAGG/CCTT	10077	4.29	6121	4.87	5211	3.88	7512	6.13
	ACAG/CTGT	8601	3.66	4441	3.53	4462	3.32	3135	2.56
	ATCC/ATGG	3674	1.56	2272	1.81	1999	1.49	2188	1.79
	ACTC/AGTG	2297	0.98	1217	0.97	1444	1.08	252	0.21
	AGGG/CCCT	1748	0.74	620	0.49	758	0.56	670	0.55
	AATG/ATTC	1233	0.53	935	0.74	638	0.48	1862	1.52
	ACCT/AGGT	1126	0.48	717	0.57	667	0.50	523	0.43
	ACGC/CGTG	891	0.38	445	0.35	452	0.34	220	0.18
	AATC/ATTG	486	0.21	299	0.24	229	0.17	588	0.48
	AGGC/CCTG	392	0.17	188	0.15	169	0.13	239	0.20
	AATT/AATT	385	0.16	316	0.25	222	0.17	812	0.66
	AAGC/CTTG	358	0.15	219	0.17	166	0.12	305	0.25
	AACC/GGTT	235	0.10	139	0.11	114	0.08	230	0.19
	AGCC/CTGG	232	0.10	101	0.08	126	0.09	70	0.06
	AGCT/AGCT	153	0.07	78	0.06	78	0.06	60	0.05
	AGCG/CGCT	144	0.06	36	0.03	47	0.03	22	0.02
	ACCC/GGGT	94	0.04	25	0.02	32	0.02	59	0.05
	ACGG/CCGT	68	0.03	14	0.01	3	0.00	19	0.02
	AACT/AGTT	32	0.01	25	0.02	13	0.01	57	0.05
	AAGT/ACTT	27	0.01	12	0.01	14	0.01	44	0.04
	ATGC/ATGC	25	0.01	19	0.02	12	0.01	36	0.03
	AACG/CGTT	20	0.01	12	0.01	18	0.01	26	0.02
	ATCG/ATCG	18	0.01	15	0.01	4	0.00	7	0.01
	ACTG/AGTC	12	0.01	14	0.01	11	0.01	11	0.01
	CCGG/CCGG	2	0.00	4	0.00	2	0.00	---	---
	ACCG/CGGT	1	0.00	3	0.00	---	---	4	0.00
	CCCG/CGGG	1	0.00	2	0.00	---	---	3	0.00
	ACGT/ACGT	---	---	---	---	---	---	1	0.00
Penta-SSR	AATAG/ATTCT	23493	69.76	16780	69.98	13516	71.39	33432	71.36
	AACAG/CTGTT	3297	9.79	2224	9.28	1787	9.44	5092	10.87
	AAAAC/GTTTT	2346	6.97	1633	6.81	1188	6.28	3226	6.89
	AAAAT/ATTTT	1452	4.31	1313	5.48	943	4.98	2529	5.40

AAAAG/CTTTT	1135	3.37	854	3.56	553	2.92	1263	2.70
AAGAG/CTCTT	325	0.97	175	0.73	166	0.88	146	0.31
AAAGG/CCTTT	261	0.78	197	0.82	104	0.55	296	0.63
ACACC/GGTGT	200	0.59	129	0.54	120	0.63	19	0.04
AGAGG/CCTCT	170	0.50	111	0.46	59	0.31	28	0.06
AAACC/GGTTT	100	0.30	87	0.36	78	0.41	130	0.28
AAAGC/CTTTG	135	0.40	39	0.16	66	0.35	39	0.08
AAATT/AATTT	115	0.34	69	0.29	54	0.29	152	0.32
AGGGG/CCCCT	113	0.34	49	0.20	18	0.10	6	0.01
AACAC/GTGTT	96	0.29	49	0.20	61	0.32	11	0.02
AATAC/ATTGT	65	0.19	7	0.03	9	0.05	36	0.08
AAGGG/CCCTT	63	0.19	41	0.17	30	0.16	45	0.10
ACATC/ATGTG	40	0.12	17	0.07	35	0.18	6	0.01
AACAT/ATGTT	34	0.10	13	0.05	27	0.14	30	0.06
AATGG/ATTCC	34	0.10	23	0.10	11	0.06	25	0.05
AAGAT/ATCTT	21	0.06	---	---	1	0.01	6	0.01
AAATC/ATTTG	16	0.05	9	0.04	5	0.03	23	0.05
AAGAC/CTTGT	16	0.05	10	0.04	4	0.02	14	0.03
AATAT/ATATT	16	0.05	9	0.04	16	0.08	33	0.07
ACCCC/GGGGT	13	0.04	3	0.01	6	0.03	9	0.02
ACTCT/AGAGT	13	0.04	7	0.03	8	0.04	20	0.04
ACTAG/AGTCT	12	0.04	7	0.03	10	0.05	11	0.02
AAACT/AGTTT	11	0.03	8	0.03	7	0.04	11	0.02
ACTCC/AGTGG	10	0.03	7	0.03	5	0.03	5	0.01
AGGAT/ATCCT	9	0.03	15	0.06	7	0.04	11	0.02
AAATG/ATTTC	7	0.02	13	0.05	5	0.03	40	0.09
ACCAG/CTGGT	7	0.02	2	0.01	---	---	---	---
ATCCC/ATGGG	7	0.02	2	0.01	---	---	5	0.01
AATTC/AATTG	6	0.02	---	---	3	0.02	5	0.01
AGGGC/CCCTG	6	0.02	2	0.01	2	0.01	5	0.01
AATCT/AGATT	4	0.01	12	0.05	3	0.02	13	0.03
AGCCC/CTGGG	4	0.01	4	0.02	3	0.02	15	0.03

ATATC/ATATG	3	0.01	7	0.03	---	---	11	0.02
AAACG/CGTTT	2	0.01	---	---	---	---	---	---
ACACT/AGTGT	2	0.01	5	0.02	2	0.01	2	0.00
ACAGG/CCTGT	2	0.01	2	0.01	---	---	7	0.01
ACTGG/AGTCC	2	0.01	---	---	1	0.01	3	0.01
AGATG/ATCTC	2	0.01	2	0.01	2	0.01	7	0.01
AGCCT/AGGCT	2	0.01	2	0.01	---	---	4	0.01
CCCCG/CGGGG	2	0.01	4	0.02	1	0.01	5	0.01
AAAGT/ACTTT	1	0.00	1	0.00	4	0.02	3	0.01
AATCG/ATTCG	1	0.00	12	0.05	1	0.01	7	0.01
AATGC/ATTGC	1	0.00	1	0.00	---	---	3	0.01
AATGT/ACATT	1	0.00	---	---	1	0.01	1	0.00
ACCCG/CGGGT	1	0.00	3	0.01	2	0.01	1	0.00
ACGAG/CGTCT	1	0.00	---	---	2	0.01	3	0.01
AGAGC/CTCTG	1	0.00	---	---	---	---	---	---
AACCC/GGGTT	---	---	---	---	---	---	3	0.01
AACCT/AGGTT	---	---	1	0.00	1	0.01	---	---
AACGT/ACGTT	---	---	---	---	---	---	2	0.00
AACTC/AGTTG	---	---	---	---	1	0.01	---	---
AAGCC/CTTGG	---	---	---	---	---	---	4	0.01
AAGGC/CCTTG	---	---	---	---	---	---	5	0.01
AAGGT/ACCTT	---	---	1	0.00	---	---	---	---
AATCC/ATTGG	---	---	---	---	2	0.01	6	0.01
ACAGC/CTGTG	---	---	2	0.01	---	---	1	0.00
ACAGT/ACTGT	---	---	1	0.00	---	---	---	---
ACATG/ATGTC	---	---	---	---	---	---	4	0.01
ACCAT/ATGGT	---	---	2	0.01	1	0.01	2	0.00
ACCCT/AGGGT	---	---	---	---	---	---	4	0.01
ACCTC/AGGTG	---	---	1	0.00	---	---	1	0.00
ACGGG/CCCGT	---	---	---	---	---	---	7	0.01
ACGTC/ACGTG	---	---	---	---	---	---	1	0.00
ACTAT/AGTAT	---	---	5	0.02	1	0.01	4	0.01
AGATC/ATCTG	---	---	1	0.00	---	---	2	0.00

	AGCAT/ATGCT	---	---	1	0.00	---	---	3	0.01
	AGGCC/CCTGG	---	---	1	0.00	---	---	2	0.00
	AGGCG/CCTCG	---	---	1	0.00	---	---	---	---
	ATGCC/ATGGC	---	---	1	0.00	---	---	4	0.01
Hexa-SSR	AAGGAG/CCTTCT	5985	33.83	4078	57.53	4184	45.49	651	27.33
	AAGAGG/CCTCTT	2588	14.63	540	7.62	1193	12.97	223	9.36
	AAGCAG/CTGCTT	2325	13.14	406	5.73	257	2.79	90	3.78
	AGAGCG/CGCTCT	2296	12.98	59	0.83	1356	14.74	84	3.53
	ACACCC/GGGTGT	927	5.24	444	6.26	462	5.02	72	3.02
	AAGACG/CGTCTT	769	4.35	158	2.23	512	5.57	86	3.61
	ACAGAG/CTCTGT	430	2.43	263	3.71	283	3.08	124	5.21
	ACACGC/CGTGTG	334	1.89	152	2.14	132	1.44	58	2.43
	AAAAAC/GTTTTT	254	1.44	130	1.83	146	1.59	271	11.38
	AAAAAG/CTTTTT	225	1.27	36	0.51	46	0.50	40	1.68
	AAGCGG/CCGCTT	198	1.12	30	0.42	40	0.43	---	---
	ACACAG/CTGTGT	195	1.10	73	1.03	92	1.00	26	1.09
	AGAGGG/CCCTCT	177	1.00	151	2.13	91	0.99	28	1.18
	ACAGCC/CTGTGG	166	0.94	101	1.42	66	0.72	211	8.86
	AGGCGG/CCGCCT	92	0.52	56	0.79	45	0.49	3	0.13
	ACTCGG/AGTCCG	66	0.37	29	0.41	50	0.54	3	0.13
	AGAGAT/ATCTCT	66	0.37	20	0.28	3	0.03	3	0.13
	AAGGGG/CCCCTT	64	0.36	38	0.54	36	0.39	11	0.46
	AACAGC/CTGTTG	61	0.34	11	0.16	6	0.07	10	0.42
	AAAGAG/CTCTTT	37	0.21	6	0.08	19	0.21	8	0.34
	AAGAAT/ATTCTT	37	0.21	2	0.03	3	0.03	2	0.08
	AACCCT/AGGGTT	36	0.20	48	0.68	23	0.25	124	5.21
	ACGGCC/CCGTGG	23	0.13	1	0.01	3	0.03	2	0.08
	AGATAT/ATATCT	23	0.13	50	0.71	27	0.29	32	1.34
	ACTCTC/AGAGTG	21	0.12	2	0.03	---	---	---	---
	ACATAT/ATATGT	20	0.11	16	0.23	13	0.14	18	0.76
	AACAAG/CTTGTT	19	0.11	1	0.01	---	---	---	---
	AAGATG/ATCTTC	19	0.11	---	---	1	0.01	---	---

AAAACC/GGTTTT	18	0.10	4	0.06	7	0.08	12	0.50
AAGGCG/CCTTCG	18	0.10	---	---	4	0.04	1	0.04
AAGTAG/ACTTCT	17	0.10	1	0.01	---	---	---	---
ACACTC/AGTGTG	13	0.07	8	0.11	3	0.03	4	0.17
AGCAGG/CCTGCT	13	0.07	7	0.10	3	0.03	1	0.04
AAATAT/ATATTT	12	0.07	14	0.20	13	0.14	5	0.21
AACATC/ATGTTG	12	0.07	9	0.13	---	---	---	---
AAAAAT/ATTTTT	11	0.06	7	0.10	20	0.22	28	1.18
AGGGCG/CCCTCG	11	0.06	6	0.08	8	0.09	18	0.76
AACCAC/GGTTGT	10	0.06	---	---	---	---	---	---
ACCCCC/GGGGGT	9	0.05	---	---	1	0.01	5	0.21
ACCTCC/AGGTGG	7	0.04	1	0.01	---	---	---	---
AAGTAT/ACTTAT	6	0.03	---	---	1	0.01	---	---
ACACAT/ATGTGT	6	0.03	10	0.14	3	0.03	2	0.08
AGCGCC/CGCTGG	6	0.03	1	0.01	1	0.01	14	0.59
AGGATG/ATCCTC	6	0.03	1	0.01	---	---	---	---
AATACT/AGTATT	5	0.03	1	0.01	3	0.03	1	0.04
AGCATC/ATGCTG	5	0.03	---	---	---	---	---	---
AAAGGG/CCCTTT	4	0.02	3	0.04	1	0.01	2	0.08
AAAGGT/ACCTTT	4	0.02	---	---	---	---	---	---
AATGAT/ATCATT	3	0.02	---	---	---	---	---	---
ACCCAG/CTGGGT	3	0.02	---	---	1	0.01	---	---
AGGGGG/CCCCCT	3	0.02	1	0.01	---	---	---	---
AACAAT/ATTGTT	2	0.01	1	0.01	---	---	2	0.08
AACATG/ATGTTC	2	0.01	---	---	---	---	---	---
AACGAC/CGTTGT	2	0.01	6	0.08	3	0.03	1	0.04
AATAGT/ACTATT	2	0.01	1	0.01	---	---	2	0.08
ACCCTC/AGGGTG	2	0.01	---	---	1	0.01	1	0.04
ACCTCT/AGAGGT	2	0.01	---	---	---	---	---	---
ACTCCT/AGGAGT	2	0.01	---	---	---	---	1	0.04
ACTGCT/AGCAGT	2	0.01	1	0.01	---	---	1	0.04
AGATGG/ATCTCC	2	0.01	3	0.04	1	0.01	---	---
AGCCCT/AGGGCT	2	0.01	---	---	4	0.04	---	---

AAAACT/AGTTTT	1	0.01	---	---	---	---	2	0.08
AAAAGG/CCTTTT	1	0.01	1	0.01	1	0.01	4	0.17
AAAATT/AATTTT	1	0.01	---	---	---	---	---	---
AACACC/GGTGTT	1	0.01	6	0.08	3	0.03	1	0.04
AAGTGT/ACACTT	1	0.01	---	---	---	---	---	---
ACACCT/AGGTGT	1	0.01	---	---	---	---	---	---
ACAGAT/ATCTGT	1	0.01	3	0.04	1	0.01	5	0.21
ACAGGG/CCCTGT	1	0.01	2	0.03	1	0.01	---	---
ACCAGC/CTGGTG	1	0.01	---	---	---	---	---	---
ACCATC/ATGGTG	1	0.01	---	---	---	---	---	---
ACCCGC/CGGGTG	1	0.01	1	0.01	3	0.03	---	---
ACCGCC/CGGTGG	1	0.01	---	---	---	---	---	---
ACGAGG/CCTCGT	1	0.01	---	---	1	0.01	1	0.04
ACTCCG/AGTCGG	1	0.01	---	---	---	---	---	---
ACTGCC/AGTGGC	1	0.01	1	0.01	1	0.01	7	0.29
AGCCGC/CGGCTG	1	0.01	---	---	---	---	---	---
AGCGGC/CCGCTG	1	0.01	8	0.11	10	0.11	---	---
AGGGCC/CCCTGG	1	0.01	1	0.01	---	---	1	0.04
AAAAGC/CTTTTG	---	---	1	0.01	2	0.02	1	0.04
AAAATC/ATTTTG	---	---	1	0.01	1	0.01	1	0.04
AAAATG/ATTTTC	---	---	2	0.03	---	---	---	---
AAAGAC/CTTTGT	---	---	---	---	---	---	2	0.08
AAAGCC/CTTTGG	---	---	---	---	---	---	5	0.21
AAATAC/ATTTGT	---	---	---	---	---	---	3	0.13
AAATAG/ATTTCT	---	---	---	---	---	---	1	0.04
AAATCT/AGATTT	---	---	2	0.03	---	---	3	0.13
AACATT/AATGTT	---	---	---	---	---	---	1	0.04
AACTAC/AGTTGT	---	---	63	0.89	---	---	---	---
AACTAG/AGTTCT	---	---	---	---	---	---	2	0.08
AAGCTG/AGCTTC	---	---	---	---	---	---	2	0.08
AAGGGC/CCCTTG	---	---	---	---	---	---	1	0.04
AATACC/ATTGGT	---	---	---	---	---	---	1	0.04

AATCAT/ATGATT	---	---	1	0.01	---	---	---	---
AATGAC/ATTGTC	---	---	---	---	---	---	1	0.04
AATTAG/AATTCT	---	---	---	---	---	---	3	0.13
ACCAGG/CCTGGT	---	---	---	---	---	---	1	0.04
ACCCTG/AGGGTC	---	---	2	0.03	---	---	5	0.21
ACCGGC/CCGGTG	---	---	1	0.01	---	---	---	---
ACCGGG/CCCGGT	---	---	---	---	---	---	1	0.04
ACGGAG/CCGTCT	---	---	2	0.03	---	---	19	0.80
ACTAGC/AGTGCT	---	---	---	---	---	---	1	0.04
ACTATC/AGTGAT	---	---	---	---	---	---	1	0.04
AGAGGC/CCTCTG	---	---	---	---	---	---	3	0.13
AGCCCC/CTGGGG	---	---	2	0.03	2	0.02	6	0.25
AGCCTC/AGGCTG	---	---	1	0.01	2	0.02	4	0.17
AGCTCC/AGCTGG	---	---	---	---	---	---	3	0.13
AGCTGC/AGCTGC	---	---	---	---	---	---	1	0.04
AGGCCG/CCTCGG	---	---	---	---	1	0.01	2	0.08
AGGCGC/CCTGCG	---	---	---	---	1	0.01	1	0.04
AGGGAT/ATCCCT	---	---	---	---	---	---	1	0.04
ATATCC/ATATGG	---	---	---	---	---	---	2	0.08
ATCCCC/ATGGGG	---	---	---	---	---	---	1	0.04
CCCCCG/CGGGGG	---	---	---	---	1	0.01	---	---
CCCCGG/CCGGGG	---	---	---	---	---	---	1	0.04

[a] Counted in each types of SSR.

Supplementary Table S2. Details of repeat units in each types of SSRs.

7 Coding regions of genes affected by SSRs

Cluster			Overlapping region in gene				Associated gene information			
Chr	Start	End	Chr	Start	End	Type	ID (Ensembl)	Name	Type	Description
1	2113086	2113549	1	2113534	2113614	CDS	ENSSSCG00000004023	FGFR1OP	protein_coding	FGFR1 oncogene partner
1	7368613	7368697	1	7368103	7369489	3'UTR	ENSSSCG00000004044	IGF2R	protein_coding	insulin like growth factor 2 receptor
1	7684097	7684415	1	7684076	7684192	CDS	ENSSSCG00000040317	SOD2	protein_coding	superoxide dismutase 2
1	15889610	15889900	1	15889611	15889643	CDS	ENSSSCG00000004094	PPP1R14C	protein_coding	protein phosphatase 1 regulatory inhibitor subunit 14C
1	19671001	19671287	1	19671110	19671181	CDS	ENSSSCG00000004120	SHPRH	protein_coding	SNF2 histone linker PHD RING helicase
1	20804671	20804968	1	20804539	20804689	CDS	ENSSSCG00000004123	UTRN	protein_coding	utrophin
1	27499055	27499758	1	27499706	27499818	CDS	ENSSSCG00000026041	MAP3K5	protein_coding	mitogen-activated protein kinase kinase
1	30945026	30945671	1	30945448	30945604	CDS	ENSSSCG00000004178	SLC18B1	protein_coding	solute carrier family 18 member B1
1	31722354	31722693	1	31722508	31726531	3'UTR	ENSSSCG00000004193	ENPP1	protein_coding	ectonucleotide pyrophosphatase/phosphodiesterase 1
1	31906774	31907446	1	31907178	31907257	CDS	ENSSSCG00000025992	ENPP3	protein_coding	ectonucleotide pyrophosphatase/phosphodiesterase 3
1	32050825	32051495	1	32051209	32051376	5'UTR	ENSSSCG00000004195	ARG1	protein_coding	arginase 1
1	33937259	33937577	1	33937353	33937487	CDS	ENSSSCG00000033894		protein_coding	laminin subunit alpha 2
1	34002000	34002362	1	34002332	34002459	CDS	ENSSSCG00000033894		protein_coding	laminin subunit alpha 2
1	35776997	35777265	1	35774048	35778966	3'UTR	ENSSSCG00000004215	KIAA0408	protein_coding	KIAA0408 ortholog
1	37019160	37019439	1	37019177	37019199	CDS	ENSSSCT00000047133			
1	44426151	44426754	1	44426130	44426190	CDS	ENSSSCT00000029401			
1	49273568	49274342	1	49273530	49273638	CDS	ENSSSCG00000004270	ADGRB3	protein_coding	adhesion G protein-coupled receptor B3
1	49472040	49472705	1	49472386	49472489	CDS	ENSSSCG00000004270	ADGRB3	protein_coding	adhesion G protein-coupled receptor B3
1	63914975	63915250	1	63911539	63915353	3'UTR	ENSSSCG00000035240	GPR63	protein_coding	G protein-coupled receptor 63
1	63914975	63915250	1	63911310	63915353	3'UTR	ENSSSCG00000035240	GPR63	protein_coding	G protein-coupled receptor 63
1	65848373	65848608	1	65847289	65848875	3'UTR	ENSSSCT00000004803			
1	66391474	66391590	1	66390523	66391979	3'UTR	ENSSSCG00000004351	USP45	protein_coding	ubiquitin specific peptidase 45
1	66391474	66391590	1	66390568	66391979	3'UTR	ENSSSCG00000004351	USP45	protein_coding	ubiquitin specific peptidase 45
1	66465507	66465869	1	66464204	66465641	3'UTR	ENSSSCG00000038757		protein_coding	thiosulfate sulfurtransferase like domain containing 3
1	71295361	71295496	1	71295217	71299820	3'UTR	ENSSSCG00000037612	LIN28B	protein_coding	lin-28 homolog B
1	72312636	72313054	1	72312938	72313427	5'UTR	ENSSSCG00000004369	PRDM1	protein_coding	PR/SET domain 1
1	72415058	72415133	1	72413994	72417326	3'UTR	ENSSSCG00000004370	ATG5	protein_coding	autophagy related 5
1	73844128	73844438	1	73843000	73850016	3'UTR	ENSSSCG00000033392	SCML4	protein_coding	Scm polycomb group protein like 4
1	73844128	73844438	1	73843006	73846087	3'UTR	ENSSSCG00000033392	SCML4	protein_coding	Scm polycomb group protein like 4
1	76247856	76248551	1	76248278	76250754	3'UTR	ENSSSCG00000004402	METTL24	protein_coding	methyltransferase like 24
1	76422163	76422327	1	76421581	76423600	3'UTR	ENSSSCG00000004399	SLC22A16	protein_coding	solute carrier family 22 member 16
1	77625740	77626091	1	77625795	77625944	CDS	ENSSSCG00000004421	FYN	protein_coding	FYN proto-oncogene%2C Src family tyrosine kinase
1	82998126	82998359	1	82996827	83000515	3'UTR	ENSSSCG00000029129	RWDD2A	protein_coding	RWD domain containing 2A
1	86973740	86974320	1	86974023	86974073	CDS	ENSSSCG00000004470	HMGN3	protein_coding	high mobility group nucleosomal binding domain 3
1	89863886	89864586	1	89864399	89864569	CDS	ENSSSCG00000004477	IMPG1	protein_coding	interphotoreceptor matrix proteoglycan 1
1	89863886	89864586	1	89864399	89864599	CDS	ENSSSCG00000004477	IMPG1	protein_coding	interphotoreceptor matrix proteoglycan 1
1	89863886	89864586	1	89864399	89864611	CDS	ENSSSCG00000004477	IMPG1	protein_coding	interphotoreceptor matrix proteoglycan 1
1	89863886	89864586	1	89864399	89864602	CDS	ENSSSCG00000004477	IMPG1	protein_coding	interphotoreceptor matrix proteoglycan 1
1	90685976	90686384	1	90684782	90688161	3'UTR	ENSSSCG00000033155	TMEM30A	protein_coding	transmembrane protein 30A
1	92202799	92203520	1	92203431	92203661	CDS	ENSSSCG00000004485	CD109	protein_coding	CD109 molecule
1	95284663	95285075	1	95285004	95285178	CDS	ENSSSCG00000004491	SLC14A2	protein_coding	solute carrier family 14 member 2
1	96313626	96313891	1	96313614	96313700	CDS	ENSSSCG00000032053	ST8SIA5	protein_coding	ST8 alpha-N-acetyl-neuraminide alpha-2%2C8-sialyltransferase 5
1	99417114	99417376	1	99417231	99418449	3'UTR	ENSSSCG00000004511		protein_coding	myosin VB
1	100630551	100630792	1	100626714	100632822	3'UTR	ENSSSCT00000045286			
1	100630551	100630792	1	100626714	100632822	3'UTR	ENSSSCT00000059572			
1	106692886	106693625	1	106692613	106696638	3'UTR	ENSSSCG00000038971	ST8SIA3	protein_coding	ST8 alpha-N-acetyl-neuraminide alpha-2%2C8-sialyltransferase 3
1	106692886	106693625	1	106692613	106697980	3'UTR	ENSSSCG00000038971	ST8SIA3	protein_coding	ST8 alpha-N-acetyl-neuraminide alpha-2%2C8-sialyltransferase 3
1	107868784	107869581	1	107869296	107869402	CDS	ENSSSCG00000004558	CSNK1G1	protein_coding	casein kinase 1 gamma 1
1	108366717	108366933	1	108366580	108366785	CDS	ENSSSCG00000004561	HERC1	protein_coding	HECT and RLD domain containing E3 ubiquitin protein ligase family member 1
1	108987566	108988014	1	108987764	108987846	CDS	ENSSSCG00000004570	TPM1	protein_coding	tropomyosin 1
1	108987566	108988014	1	108985778	108987763	3'UTR	ENSSSCG00000004570	TPM1	protein_coding	tropomyosin 1
1	110060296	110060965	1	110060666	110060830	CDS	ENSSSCG00000036431	VPS13C	protein_coding	vacuolar protein sorting 13 homolog C
1	110084657	110085283	1	110085031	110085194	CDS	ENSSSCG00000036431	VPS13C	protein_coding	vacuolar protein sorting 13 homolog C
1	115553376	115554292	1	115553445	115553579	CDS	ENSSSCG00000004602	TEX9	protein_coding	testis expressed 9
1	116396515	116397010	1	116396390	116396621	CDS	ENSSSCG00000004608	DNAAF4	protein_coding	dynein axonemal assembly factor 4
1	119384632	119385275	1	119384545	119384719	CDS	ENSSSCG00000004620	MYO5A	protein_coding	myosin VA
1	119957127	119957283	1	119956596	119963815	3'UTR	ENSSSCG00000004628	TMOD2	protein_coding	tropomodulin 2
1	122503631	122504525	1	122503953	122504076	CDS	ENSSSCG00000004651	GALK2	protein_coding	fibroblast growth factor 7
1	122503631	122504525	1	122503953	122504038	CDS	ENSSSCG00000004651	GALK2	protein_coding	fibroblast growth factor 7
1	122503631	122504525	1	122504039	122504076	5'UTR	ENSSSCG00000004651	GALK2	protein_coding	fibroblast growth factor 7
1	123448799	123449308	1	123448516	123451275	3'UTR	ENSSSCG00000032141	DUT	protein_coding	deoxyuridine triphosphatase
1	123536718	123537512	1	123537334	123537418	CDS	ENSSSCG00000004659	SLC12A1	protein_coding	solute carrier family 12 member 1
1	126663798	126664042	1	126663663	126663838	CDS	ENSSSCG00000004679	SORD	protein_coding	sorbitol dehydrogenase
1	129039517	129040089	1	129039956	129040057	CDS	ENSSSCG00000004729		protein_coding	glucosidase alpha%2C neutral C
1	130236442	130236476	1	130236347	130236529	CDS	ENSSSCG00000004753	INO80	protein_coding	INO80 complex subunit
1	130657817	130658043	1	130657750	130657818	3'UTR	ENSSSCG00000039107		protein_coding	kinetochore scaffold 1
1	130657817	130658043	1	130657750	130657818	CDS	ENSSSCT00000039183			
1	132742947	132743570	1	132742911	132743016	CDS	ENSSSCG00000004791	RASGRP1	protein_coding	RAS guanyl releasing protein 1

1	132742947	132743570	1	132743036	132743588	5'UTR	ENSSSCG00000004791	RASGRP1	protein_coding	RAS guanyl releasing protein 1
1	132742947	132743570	1	132742911	132743024	5'UTR	ENSSSCG00000004791	RASGRP1	protein_coding	RAS guanyl releasing protein 1
1	137690845	137690967	1	137690406	137691058	5'UTR	ENSSSCG00000030560	IGF1R	protein_coding	insulin like growth factor 1 receptor
1	141664258	141665140	1	141665071	141665151	CDS	ENSSSCG00000004830	ATP10A	protein_coding	ATPase phospholipid transporting 10A
1	141985480	141985601	1	141984712	141986547	3'UTR	ENSSSCG00000004832	UBE3A	protein_coding	ubiquitin protein ligase E3A
1	142804092	142804744	1	142803701	142807511	3'UTR	ENSSSCG00000004839		protein_coding	cholinergic receptor nicotinic alpha 7
1	142805715	142805952	1	142803701	142807511	3'UTR	ENSSSCG00000004839		protein_coding	cholinergic receptor nicotinic alpha 7
1	145310995	145311653	1	145311332	145311455	CDS	ENSSSCG00000005781	TARSL2	protein_coding	threonyl-tRNA synthetase like 2
1	145792805	145792822	1	145792472	145793410	CDS	ENSSSCG00000005787	CTDP1	protein_coding	CTD phosphatase subunit 1
1	152722256	152722341	1	152722151	152722263	CDS	ENSSSCG00000032574	RTTN	protein_coding	rotatin
1	157878503	157879190	1	157879071	157879190	CDS	ENSSSCG00000004890	SERPINB2	protein_coding	serpin family B member 2
1	158020399	158020749	1	158020725	158020842	CDS	ENSSSCG00000022337	SERPINB11	protein_coding	serpin family B member 11
1	163083665	163084071	1	163083021	163085423	5'UTR	ENSSSCT00000039321			
1	163083665	163084071	1	163084039	163091014	3'UTR	ENSSSCG00000038071	RASL12	protein_coding	RAS like family 12
1	165521714	165522429	1	165522393	165522457	CDS	ENSSSCG00000004955	MAP2K5	protein_coding	mitogen-activated protein kinase kinase 5
1	168759195	168759798	1	168759175	168759275	5'UTR	ENSSSCT00000045716			
1	176861229	176861992	1	176861468	176861616	CDS	ENSSSCG00000005002	MDGA2	protein_coding	MAM domain containing glycosylphosphatidylinositol anchor 2
1	179582653	179582913	1	179582747	179582805	CDS	ENSSSCG00000032489	KLHDC1	protein_coding	kelch domain containing 1
1	180034597	180035254	1	180034432	180034599	5'UTR	ENSSSCG00000005016	L2HGDH	protein_coding	L-2-hydroxyglutarate dehydrogenase [Source:NCBI gene;Acc:100152180]
1	180034597	180035254	1	180034432	180034599	CDS	ENSSSCG00000005016	L2HGDH	protein_coding	L-2-hydroxyglutarate dehydrogenase
1	180755820	180756539	1	180756073	180756226	CDS	ENSSSCG00000005025	TRIM9	protein_coding	tripartite motif containing 9
1	181636793	181637103	1	181636864	181636881	CDS	ENSSSCT00000062421			
1	194095907	194096340	1	194096241	194096443	CDS	ENSSSCG00000005110	SYNE2	protein_coding	spectrin repeat containing nuclear envelope protein 2
1	203331743	203331761	1	203328931	203333767	3'UTR	ENSSSCG00000005167	SLC24A2	protein_coding	solute carrier family 24 member 2
1	203710726	203711138	1	203709021	203713121	3'UTR	ENSSSCG00000035863	PLIN2	protein_coding	perilipin 2
1	217351378	217351574	1	217350953	217352499	3'UTR	ENSSSCG00000005221	SPATA6L	protein_coding	spermatogenesis associated 6 like
1	222758961	222758989	1	222758442	222762146	3'UTR	ENSSSCG00000005250	APBA1	protein_coding	amyloid beta precursor protein binding family A member 1
1	223034655	223034991	1	223032881	223037616	3'UTR	ENSSSCG00000005252	PTAR1	protein_coding	protein prenyltransferase alpha subunit repeat containing 1
1	235895977	235896089	1	235895623	235896708	3'UTR	ENSSSCG00000005301	FAM214B	protein_coding	family with sequence similarity 214 member
1	235895977	235896089	1	235895615	235896708	3'UTR	ENSSSCG00000005301	FAM214B	protein_coding	family with sequence similarity 214 member
1	235895977	235896089	1	235895616	235896997	3'UTR	ENSSSCG00000005301	FAM214B	protein_coding	family with sequence similarity 214 member
1	236897284	236897314	1	236896857	236898276	3'UTR	ENSSSCG00000005338	RECK	protein_coding	reversion inducing cysteine rich protein with kazal motifs
1	237575467	237575619	1	237575059	237579234	3'UTR	ENSSSCG00000036086	PAX5	protein_coding	paired box 5
1	237575467	237575619	1	237575061	237581626	3'UTR	ENSSSCG00000036086	PAX5	protein_coding	paired box 5
1	238468451	238468834	1	238468783	238468885	CDS	ENSSSCG00000005352	FRMPD1	protein_coding	FERM and PDZ domain containing 1
1	252824925	252825147	1	252825048	252825268	CDS	ENSSSCG00000005465	SUSD1	protein_coding	sushi domain containing 1
1	254153826	254154397	1	254153444	254156331	3'UTR	ENSSSCG00000005480	RGS3	protein_coding	regulator of G protein signaling 3
1	259488005	259488591	1	259488319	259488536	CDS	ENSSSCG00000005504	BRINP1	protein_coding	BMP/retinoic acid inducible neural specific
1	259488005	259488591	1	259488537	259488586	5'UTR	ENSSSCG00000005504	BRINP1	protein_coding	BMP/retinoic acid inducible neural specific
1	261897404	261897434	1	261896308	261902333	3'UTR	ENSSSCG00000038036	TTLL11	protein_coding	
1	267288220	267288327	1	267286729	267291145	3'UTR	ENSSSCG00000035968	LMX1B	protein_coding	LIM homeobox transcription factor 1 beta
1	268238209	268238416	1	268237679	268240605	3'UTR	ENSSSCG00000005620	SH2D3C	protein_coding	SH2 domain containing 3C
1	268238209	268238416	1	268237679	268239409	3'UTR	ENSSSCG00000005620	SH2D3C	protein_coding	SH2 domain containing 3C
1	271102343	271102533	1	271100024	271102404	3'UTR	ENSSSCG00000034178	AIF1L	protein_coding	allograft inflammatory factor 1 like
1	271102343	271102533	1	271102531	271102799	3'UTR	ENSSSCG00000034178	AIF1L	protein_coding	allograft inflammatory factor 1 like
1	271102343	271102533	1	271100024	271102394	3'UTR	ENSSSCG00000034178	AIF1L	protein_coding	allograft inflammatory factor 1 like
1	271102343	271102533	1	271102468	271102799	3'UTR	ENSSSCG00000034178	AIF1L	protein_coding	allograft inflammatory factor 1 like
1	271156535	271156906	1	271156409	271156669	CDS	ENSSSCG00000005711	NUP214	protein_coding	nucleoporin 214
1	272373963	272374167	1	272373601	272374066	CDS	ENSSSCG00000005730	BARHL1	protein_coding	BarH like homeobox 1
1	273334560	273334625	1	273334476	273334575	CDS	ENSSSCG00000005743	VAV2	protein_coding	vav guanine nucleotide exchange factor 2
2	4767260	4767328	2	4765387	4767688	3'UTR	ENSSSCG00000031459	KMT5B	protein_coding	lysine methyltransferase 5B
2	10738396	10738427	2	10735930	10740547	3'UTR	ENSSSCG00000013111	CD6	protein_coding	CD6 molecule
2	11955446	11955991	2	11955850	11956138	CDS	ENSSSCG00000048276		protein_coding	
2	11955446	11955991	2	11955850	11956105	CDS	ENSSSCG00000043180		protein_coding	olfactory receptor 4D11 [Source:NCBI gene;Acc:100515525]
2	12383964	12384017	2	12383543	12384192	3'UTR	ENSSSCG00000040088		protein_coding	glycine N-acyltransferase-like
2	12383964	12384017	2	12383543	12387512	3'UTR	ENSSSCG00000040088		protein_coding	glycine N-acyltransferase-like
2	12499648	12500342	2	12500272	12500644	CDS	ENSSSCG00000013155		protein_coding	ZFP91 zinc finger protein
2	12499648	12500342	2	12500134	12500644	CDS	ENSSSCG00000013155		protein_coding	ZFP91 zinc finger protein
2	12499648	12500342	2	12495198	12500133	3'UTR	ENSSSCG00000013155		protein_coding	ZFP91 zinc finger protein
2	13629613	13629645	2	13628806	13630026	3'UTR	ENSSSCG00000029059	SSRP1	protein_coding	structure specific recognition protein 1
2	14601809	14602271	2	14602169	14602393	CDS	ENSSSCG00000023709	PTPRJ	protein_coding	protein tyrosine phosphatase receptor type J
2	15752370	15752988	2	15752898	15753037	CDS	ENSSSCG00000013249	CKAP5	protein_coding	cytoskeleton associated protein 5
2	16121029	16121123	2	16117986	16122864	3'UTR	ENSSSCG00000013262	AMBRA1	protein_coding	autophagy and beclin 1 regulator 1
2	24731242	24731716	2	24731152	24731255	5'UTR	ENSSSCG00000013292	PRR5L	protein_coding	proline rich 5 like
2	25273544	25274004	2	25272622	25277064	3'UTR	ENSSSCG00000013295	TRIM44	protein_coding	tripartite motif containing 44
2	33610745	33610922	2	33610717	33610869	CDS	ENSSSCG00000013338	SLC5A12	protein_coding	solute carrier family 5 member 12
2	33610745	33610922	2	33610870	33613549	3'UTR	ENSSSCG00000013338	SLC5A12	protein_coding	solute carrier family 5 member 12
2	33610745	33610922	2	33608638	33613549	3'UTR	ENSSSCG00000013338	SLC5A12	protein_coding	solute carrier family 5 member 12
2	33686358	33686471	2	33685955	33686402	5'UTR	ENSSSCG00000013340	MUC15	protein_coding	mucin 15%2C cell surface associated
2	33698666	33699126	2	33698773	33698833	CDS	ENSSSCG00000013339	ANO3	protein_coding	anoctamin 3
2	33698666	33699126	2	33697392	33699691	3'UTR	ENSSSCG00000013340	MUC15	protein_coding	mucin 15%2C cell surface associated
2	33698666	33699126	2	33695409	33699691	3'UTR	ENSSSCG00000013340	MUC15	protein_coding	mucin 15%2C cell surface associated
2	46403373	46403494	2	46403207	46403548	3'UTR	ENSSSCG00000013399	TEAD1	protein_coding	TEA domain transcription factor 1

2	46403373	46403494	2	46400066	46403548	3'UTR	ENSSSCG00000013399	TEAD1	protein_coding	TEA domain transcription factor 1
2	47526460	47527130	2	47526958	47527071	CDS	ENSSSCG00000013402	USP47	protein_coding	ubiquitin specific peptidase 47
2	48698908	48699594	2	48697163	48701307	3'UTR	ENSSSCG00000013406	CTR9	protein_coding	CTR9 homolog%2C Paf1/RNA polymerase II complex component
2	48950360	48951001	2	48950221	48950535	CDS	ENSSSCG00000029886	LYVE1	protein_coding	lymphatic vessel endothelial hyaluronan receptor 1
2	60127914	60128019	2	60125393	60128772	3'UTR	ENSSSCT00000038819			
2	61741450	61741511	2	61741068	61745155	3'UTR	ENSSSCG00000032765		protein_coding	cytochrome P450%2C family 4%2C subfamily F%2C polypeptide 2
2	64365979	64366628	2	64365487	64369886	3'UTR	ENSSSCG00000036342		protein_coding	adhesion G protein-coupled receptor E2-like
2	64367868	64368182	2	64365487	64369886	3'UTR	ENSSSCG00000036342		protein_coding	adhesion G protein-coupled receptor E2-like
2	64369804	64370025	2	64365487	64369886	3'UTR	ENSSSCG00000036342		protein_coding	adhesion G protein-coupled receptor E2-like
2	69466724	69466785	2	69466668	69466746	CDS	ENSSSCG00000013637	QTRT1	protein_coding	queuine tRNA-ribosyltransferase catalytic subunit 1
2	69568550	69569116	2	69568766	69568788	CDS	ENSSSCG00000032334		protein_coding	
2	69568550	69569116	2	69568970	69568987	CDS	ENSSSCG00000032334		protein_coding	
2	71139389	71140019	2	71139838	71139906	CDS	ENSSSCG00000013594	FBN3	protein_coding	fibrillin 3
2	72667392	72667620	2	72666895	72668963	3'UTR	ENSSSCG00000013538	KHSRP	protein_coding	KH-type splicing regulatory protein
2	72667392	72667620	2	72666895	72668714	3'UTR	ENSSSCG00000013538	KHSRP	protein_coding	KH-type splicing regulatory protein
2	74298482	74298502	2	74295482	74300462	3'UTR	ENSSSCG00000035176	SEMA6B	protein_coding	semaphorin 6B
2	74545433	74545739	2	74544610	74545938	3'UTR	ENSSSCT00000043714			
2	75926640	75926726	2	75924763	75927234	3'UTR	ENSSSCG00000038013	DIRAS1	protein_coding	DIRAS family GTPase 1
2	76493110	76493134	2	76491527	76495927	3'UTR	ENSSSCG00000032366	MOB3A	protein_coding	MOB kinase activator 3A
2	80252255	80252683	2	80251237	80255506	3'UTR	ENSSSCG00000014034	N4BP3	protein_coding	NEDD4 binding protein 3
2	81302561	81302601	2	81297327	81302861	3'UTR	ENSSSCG00000014053	SNCB	protein_coding	synuclein beta
2	82728981	82729181	2	82729025	82729126	CDS	ENSSSCG00000014068	UTP15	protein_coding	UTP15 small subunit processome
2	85815081	85815205	2	85814352	85817505	5'UTR	ENSSSCG00000014093	CRHBP	protein_coding	corticotropin releasing hormone binding
2	85830463	85831248	2	85830441	85830598	CDS	ENSSSCG00000014093	CRHBP	protein_coding	corticotropin releasing hormone binding
2	85830463	85831248	2	85830599	85831179	3'UTR	ENSSSCG00000014093	CRHBP	protein_coding	corticotropin releasing hormone binding
2	85830463	85831248	2	85830599	85831182	3'UTR	ENSSSCG00000014093	CRHBP	protein_coding	corticotropin releasing hormone binding
2	87872255	87873029	2	87872359	87872450	CDS	ENSSSCG00000014108	BHMT	protein_coding	betaine--homocysteine S-methyltransferase 2 [Source:NCBI gene;Acc:100523776]
2	89528474	89529096	2	89528757	89529388	5'UTR	ENSSSCG00000014127	RASGRF2	protein_coding	Ras protein specific guanine nucleotide releasing factor 2
2	89528474	89529096	2	89528785	89529388	5'UTR	ENSSSCG00000014127	RASGRF2	protein_coding	Ras protein specific guanine nucleotide releasing factor 2
2	89674722	89675429	2	89674983	89675065	CDS	ENSSSCG00000014127	RASGRF2	protein_coding	Ras protein specific guanine nucleotide releasing factor 2
2	90215926	90216687	2	90215949	90215992	CDS	ENSSSCG00000035757	SSBP2	protein_coding	single stranded DNA binding protein 2
2	90215926	90216687	2	90215993	90216028	5'UTR	ENSSSCG00000035757	SSBP2	protein_coding	single stranded DNA binding protein 2
2	91465355	91466317	2	91465341	91465447	CDS	ENSSSCG00000014136	VCAN	protein_coding	X-ray repair cross complementing 4
2	97942872	97943242	2	97942450	97942923	CDS	ENSSSCG00000035025	ADGRV1	protein_coding	adhesion G protein-coupled receptor V1
2	97947167	97947700	2	97947561	97947704	CDS	ENSSSCG00000035025	ADGRV1	protein_coding	adhesion G protein-coupled receptor V1
2	101574831	101575180	2	101574896	101574989	CDS	ENSSSCG00000025286	MCTP1	protein_coding	multiple C2 and transmembrane domain containing 1
2	103387570	103388315	2	103387922	103388078	CDS	ENSSSCG00000014171	ERAP1	protein_coding	endoplasmic reticulum aminopeptidase 1
2	103746334	103746914	2	103746877	103746954	CDS	ENSSSCG00000023396	LIX1	protein_coding	limb and CNS expressed 1
2	119759101	119759162	2	119758633	119760045	3'UTR	ENSSSCG00000038141		protein_coding	toll like receptor adaptor molecule 2
2	125560482	125561066	2	125561041	125561122	CDS	ENSSSCG00000022656	SRFBP1	protein_coding	serum response factor binding protein 1
2	125690234	125690816	2	125690687	125691721	CDS	ENSSSCG00000014233		protein_coding	zinc finger protein 474
2	125690234	125690816	2	125690474	125690686	5'UTR	ENSSSCG00000014233		protein_coding	zinc finger protein 474
2	131243174	131243495	2	131243248	131243370	CDS	ENSSSCG00000014256	FBN2	protein_coding	fibrillin 2
2	134605227	134605790	2	134605397	134605417	CDS	ENSSSCG00000033210		protein_coding	
2	134633779	134634028	2	134633614	134633796	CDS	ENSSSCG00000014275	SLC22A4	protein_coding	solute carrier family 22 member 4
2	135326360	135326840	2	135326538	135326802	CDS	ENSSSCG00000014292	HSPA4	protein_coding	zinc finger CCHC-type containing 10
2	135326360	135326840	2	135326803	135328712	3'UTR	ENSSSCG00000014292	HSPA4	protein_coding	zinc finger CCHC-type containing 10
2	136927604	136927704	2	136927400	136927606	CDS	ENSSSCG00000014304	SEC24A	protein_coding	SEC24 homolog A%2C COPII coat complex component
2	140426765	140426781	2	140426180	140427159	3'UTR	ENSSSCG00000014335	REEP2	protein_coding	receptor accessory protein 2
2	143878621	143878769	2	143877340	143880981	3'UTR	ENSSSCG00000036322	SPRY4	protein_coding	sprouty RTK signaling antagonist 4
2	143878621	143878769	2	143877346	143880981	3'UTR	ENSSSCG00000036322	SPRY4	protein_coding	sprouty RTK signaling antagonist 4
2	147170625	147170931	2	147170889	147171049	CDS	ENSSSCG00000014406	PRELID2	protein_coding	PRELI domain containing 2
2	148672747	148672830	2	148672587	148672757	CDS	ENSSSCG00000036549	DPYSL3	protein_coding	dihydropyrimidinase like 3
2	150917729	150918021	2	150917780	150917880	CDS	ENSSSCG00000014437	PPARGC1B	protein_coding	PPARG coactivator 1 beta
2	150917729	150918021	2	150917881	150919366	3'UTR	ENSSSCG00000014437	PPARGC1B	protein_coding	PPARG coactivator 1 beta
2	151373337	151373517	2	151373379	151373530	CDS	ENSSSCG00000014450	TCOF1	protein_coding	treacle ribosome biogenesis factor 1
3	7720173	7720663	3	7720164	7720413	CDS	ENSSSCG00000007639		protein_coding	
3	7720173	7720663	3	7720453	7721096	CDS	ENSSSCG00000007639		protein_coding	
3	8535295	8535606	3	8533627	8535521	3'UTR	ENSSSCG00000023255	MOSPD3	protein_coding	motile sperm domain containing 3
3	9732517	9733013	3	9732871	9732982	CDS	ENSSSCT00000008425			
3	10767313	10767541	3	10767494	10767609	CDS	ENSSSCG00000027342	BAZ1B	protein_coding	bromodomain adjacent to zinc finger
3	17098066	17098399	3	17098124	17098748	5'UTR	ENSSSCG00000035952	TGFB1I1	protein_coding	transforming growth factor beta 1 induced transcript 1
3	17456612	17456642	3	17456346	17459516	3'UTR	ENSSSCG00000021238	STX1B	protein_coding	syntaxin 1B
3	19653614	19653919	3	19653405	19654218	3'UTR	ENSSSCG00000007818	KDM8	protein_coding	lysine demethylase 8
3	23118243	23118353	3	23116008	23118418	3'UTR	ENSSSCG00000030424	USP31	protein_coding	ubiquitin specific peptidase 31
3	23252598	23252774	3	23251941	23256748	3'UTR	ENSSSCG00000036475	HS3ST2	protein_coding	heparan sulfate-glucosamine 3-sulfotransferase 2
3	23673114	23673194	3	23673010	23673207	5'UTR	ENSSSCG00000027967	IGSF6	protein_coding	immunoglobulin superfamily member 6
3	24653184	24653807	3	24653735	24653836	CDS	ENSSSCG00000007848		protein_coding	

3	28802270	28802296	3	28801516	28802501	3'UTR	ENSSSCG00000007878	PARN	protein_coding	poly(A)-specific ribonuclease
3	28802270	28802296	3	28801516	28802504	3'UTR	ENSSSCG00000007878	PARN	protein_coding	poly(A)-specific ribonuclease
3	28802270	28802296	3	28801516	28802505	3'UTR	ENSSSCG00000007878	PARN	protein_coding	poly(A)-specific ribonuclease
3	33129802	33130229	3	33130144	33130382	CDS	ENSSSCG00000031290	GRIN2A	protein_coding	glutamate ionotropic receptor NMDA type subunit 2A
3	44101518	44102238	3	44102040	44102153	CDS	ENSSSCG00000008096	ZC3H6	protein_coding	zinc finger CCCH-type containing 6
3	44577765	44577856	3	44577602	44577795	CDS	ENSSSCG00000008105	ANAPC1	protein_coding	anaphase promoting complex subunit 1
3	46751432	46751666	3	46750673	46751505	3'UTR	ENSSSCG00000008124	NEURL3	protein_coding	neuralized E3 ubiquitin protein ligase 3
3	47061104	47061247	3	47061111	47061172	CDS	ENSSSCG00000008126	ASTL	protein_coding	astacin like metalloendopeptidase
3	51834819	51835358	3	51834054	51837665	5'UTR	ENSSSCT00000051220			
3	53417244	53417370	3	53417126	53418480	5'UTR	ENSSSCG00000008171	NPAS2	protein_coding	neuronal PAS domain protein 2
3	54726515	54726798	3	54726437	54726541	3'UTR	ENSSSCG00000008179	REV1	protein_coding	REV1 DNA directed polymerase
3	54726515	54726798	3	54726437	54727872	3'UTR	ENSSSCG00000008179	REV1	protein_coding	REV1 DNA directed polymerase
3	54726515	54726798	3	54726315	54726541	CDS	ENSSSCG00000008179	REV1	protein_coding	REV1 DNA directed polymerase
3	54726515	54726798	3	54726315	54727872	3'UTR	ENSSSCG00000008179	REV1	protein_coding	REV1 DNA directed polymerase
3	56353241	56353383	3	56353310	56353350	5'UTR	ENSSSCG00000008192	TMEM131	protein_coding	transmembrane protein 131
3	56353241	56353383	3	56353310	56353350	CDS	ENSSSCG00000008192	TMEM131	protein_coding	transmembrane protein 131
3	58042709	58043419	3	58042701	58042764	CDS	ENSSSCG00000035478	RMND5A	protein_coding	required for meiotic nuclear division 5 homolog A
3	58314502	58314786	3	58314374	58314546	CDS	ENSSSCG00000008221	KDM3A	protein_coding	lysine demethylase 3A
3	68553837	68554399	3	68551412	68556397	3'UTR	ENSSSCG00000008275	TTC31	protein_coding	tetratricopeptide repeat domain 31
3	69161767	69161902	3	69161674	69161773	CDS	ENSSSCG00000008300	STAMBP	protein_coding	STAM binding protein
3	71840721	71840988	3	71840069	71840861	3'UTR	ENSSSCG00000020736	ADD2	protein_coding	adducin 2
3	73527603	73527759	3	73525649	73530472	3'UTR	ENSSSCG00000032071	BMP10	protein_coding	bone morphogenetic protein 10
3	75485534	75485852	3	75482261	75486309	3'UTR	ENSSSCG00000039161	MEIS1	protein_coding	Meis homeobox 1
3	75485534	75485852	3	75482261	75486593	3'UTR	ENSSSCG00000039161	MEIS1	protein_coding	Meis homeobox 1
3	77314878	77315136	3	77313080	77317323	3'UTR	ENSSSCG00000037002	SERTAD2	protein_coding	SERTA domain containing 2
3	77314878	77315136	3	77313080	77317328	3'UTR	ENSSSCG00000037002	SERTAD2	protein_coding	SERTA domain containing 2
3	80739534	80740347	3	80739889	80740007	5'UTR	ENSSSCT00000047963			
3	83535514	83536095	3	83535941	83536075	5'UTR	ENSSSCG00000008395	VRK2	protein_coding	VRK serine/threonine kinase 2
3	91984044	91984746	3	91984228	91984299	CDS	ENSSSCG00000008421		protein_coding	Sus scrofa lutropin-choriogonadotropic hormone receptor-like
3	92761879	92762179	3	92761995	92762122	CDS	ENSSSCG00000029485	FBXO11	protein_coding	F-box protein 11
3	96065821	96066651	3	96066361	96066425	CDS	ENSSSCG00000008447	CAMKMT	protein_coding	calmodulin-lysine N-methyltransferase
3	102029311	102029429	3	102027497	102029667	3'UTR	ENSSSCG00000008487	ATL2	protein_coding	atlastin GTPase 2
3	102215582	102216094	3	102215739	102219595	3'UTR	ENSSSCG00000008488	RMDN2	protein_coding	regulator of microtubule dynamics 2
3	103492645	103492959	3	103492726	103492968	5'UTR	ENSSSCG00000008501	VIT	protein_coding	vitrin
3	103492645	103492959	3	103492726	103493379	5'UTR	ENSSSCG00000008501	VIT	protein_coding	vitrin
3	103492645	103492959	3	103492726	103492967	5'UTR	ENSSSCG00000008501	VIT	protein_coding	vitrin
3	106888270	106888786	3	106888583	106888705	CDS	ENSSSCG00000008512	TTC27	protein_coding	tetratricopeptide repeat domain 27
3	107370709	107371371	3	107369159	107370736	CDS	ENSSSCT00000029026			
3	107870784	107871479	3	107871315	107871334	CDS	ENSSSCT00000037294			
3	108105186	108105211	3	108100401	108105268	3'UTR	ENSSSCG00000035987	EHD3	protein_coding	EH domain containing 3
3	110153587	110154186	3	110154012	110154147	CDS	ENSSSCG00000008535	CLIP4	protein_coding	CAP-Gly domain containing linker protein family member 4
3	110820923	110821577	3	110819947	110824933	3'UTR	ENSSSCG00000032527	FOSL2	protein_coding	FOS like 2%2C AP-1 transcription factor subunit
3	111512390	111512461	3	111512301	111512529	CDS	ENSSSCG00000008549	SLC4A1AP	protein_coding	solute carrier family 4 member 1 adaptor
3	112718952	112719285	3	112719023	112719102	CDS	ENSSSCG00000008569	HADHB	protein_coding	hydroxyacyl-CoA dehydrogenase trifunctional multienzyme complex subunit
3	118761999	118762162	3	118757921	118762766	5'UTR	ENSSSCG00000008606	OSR1	protein_coding	odd-skipped related transcription factor 1
3	120178595	120178613	3	120177895	120178674	3'UTR	ENSSSCG00000008615	VSNL1	protein_coding	visinin like 1
3	120287976	120288144	3	120287790	120288136	5'UTR	ENSSSCG00000008615	VSNL1	protein_coding	visinin like 1
4	831129	831375	4	831070	831456	CDS	ENSSSCG00000039466	SCRIB	protein_coding	scribble planar cell polarity protein
4	8360935	8361137	4	8360803	8361042	CDS	ENSSSCG00000005948	TG	protein_coding	thyroglobulin
4	8606676	8606778	4	8605571	8608372	3'UTR	ENSSSCG00000005952	LRRC6	protein_coding	leucine rich repeat containing 6
4	17582729	17582972	4	17582849	17583723	5'UTR	ENSSSCG00000005992	SHAS2	protein_coding	hyaluronan synthase 2
4	18229386	18229483	4	18228938	18229405	CDS	ENSSSCG00000013683		protein_coding	
4	29344488	29344944	4	29343452	29345385	3'UTR	ENSSSCG00000006033	EIF3E	protein_coding	eukaryotic translation initiation factor 3
4	33894310	33894489	4	33894240	33894350	CDS	ENSSSCG00000006053	BAALC	protein_coding	BAALC binder of MAP3K1 and KLF4
4	46040342	46040548	4	46040307	46040383	CDS	ENSSSCG00000031424	NECAB1	protein_coding	N-terminal EF-hand calcium binding protein
4	50314218	50314804	4	50314754	50314828	CDS	ENSSSCG00000006135	CPNE3	protein_coding	copine 3
4	56591728	56592391	4	56592318	56592359	CDS	ENSSSCG00000006156	TPD52	protein_coding	tumor protein D52
4	64330095	64330446	4	64326121	64330458	3'UTR	ENSSSCG00000040728	EYA1	protein_coding	EYA transcriptional coactivator and phosphatase 1
4	66925427	66925996	4	66925782	66925886	CDS	ENSSSCG00000006199	PREX2	protein_coding	phosphatidylinositol-3%2C4%2C5-trisphosphate dependent Rac exchange
4	68557125	68557786	4	68555871	68557905	3'UTR	ENSSSCG00000006217	DNAJC5B	protein_coding	DnaJ heat shock protein family (Hsp40) member C5 beta
4	74296260	74296621	4	74295922	74297068	3'UTR	ENSSSCG00000006237	SDCBP	protein_coding	syndecan binding protein
4	74296260	74296621	4	74294861	74298193	3'UTR	ENSSSCG00000006236	NSMAF	protein_coding	neutral sphingomyelinase activation associated factor
4	75938531	75938864	4	75937129	75938943	3'UTR	ENSSSCG00000006251	TGS1	protein_coding	trimethylguanosine synthase 1
4	78161763	78162113	4	78160260	78162371	3'UTR	ENSSSCG00000006267	PCMTD1	protein_coding	protein-L-isoaspartate (D-aspartate) O-methyltransferase domain containing 1
4	78161763	78162113	4	78160260	78163062	3'UTR	ENSSSCG00000006267	PCMTD1	protein_coding	protein-L-isoaspartate (D-aspartate) O-methyltransferase domain containing 1
4	78161763	78162113	4	78160260	78163412	3'UTR	ENSSSCG00000006267	PCMTD1	protein_coding	protein-L-isoaspartate (D-aspartate) O-methyltransferase domain containing 1

4	78161763	78162113	4	78160260	78162963	3'UTR	ENSSSCG00000006267	PCMTD1	protein_coding	protein-L-isoaspartate (D-aspartate) O-methyltransferase domain containing 1
4	80430524	80430566	4	80427687	80430926	3'UTR	ENSSSCG00000031503	PRRX1	protein_coding	paired related homeobox 1
4	80430524	80430566	4	80427688	80431065	3'UTR	ENSSSCG00000031503	PRRX1	protein_coding	paired related homeobox 1
4	80858325	80858680	4	80857344	80858920	3'UTR	ENSSSCG00000006281	METTL11B	protein_coding	methyltransferase like 11B
4	83404068	83404329	4	83403991	83404083	CDS	ENSSSCG00000006307	RCSD1	protein_coding	RCSD domain containing 1
4	83404068	83404329	4	83404084	83404102	5'UTR	ENSSSCG00000006307	RCSD1	protein_coding	RCSD domain containing 1
4	89147507	89147879	4	89147581	89147642	CDS	ENSSSCG00000030318	SDHC	protein_coding	succinate dehydrogenase complex subunit C
4	89268903	89269423	4	89268460	89269244	3'UTR	ENSSSCG00000006359	ADAMTS4	protein_coding	ADAM metallopeptidase with thrombospondin type 1 motif 4
4	89902263	89902293	4	89898730	89903942	3'UTR	ENSSSCG00000006380		protein_coding	signaling lymphocytic activation molecule family member 1
4	90263693	90263864	4	90263601	90263959	3'UTR	ENSSSCG00000032164	PEA15	protein_coding	proliferation and apoptosis adaptor protein
4	90263693	90263864	4	90263604	90265555	3'UTR	ENSSSCG00000032164	PEA15	protein_coding	proliferation and apoptosis adaptor protein
4	90283350	90283640	4	90283370	90283481	CDS	ENSSSCG00000006390	CASQ1	protein_coding	calsequestrin 1
4	90455423	90455860	4	90453453	90457406	3'UTR	ENSSSCG00000031858	KCNJ10	protein_coding	potassium voltage-gated channel subfamily J member 10
4	90564100	90564121	4	90563902	90564154	3'UTR	ENSSSCG00000006396	IGSF9	protein_coding	immunoglobulin superfamily member 9
4	90564100	90564121	4	90563902	90566185	3'UTR	ENSSSCG00000006396	IGSF9	protein_coding	immunoglobulin superfamily member 9
4	91373698	91373952	4	91373129	91375525	3'UTR	ENSSSCG00000006418		protein_coding	
4	91373698	91373952	4	91373129	91375096	3'UTR	ENSSSCG00000006418		protein_coding	
4	91658455	91658911	4	91658860	91659783	CDS	ENSSSCG00000044661		protein_coding	
4	91665984	91666329	4	91666209	91667141	CDS	ENSSSCG00000049736		protein_coding	
4	91673265	91673917	4	91673559	91674494	CDS	ENSSSCG00000048221		protein_coding	
4	91713636	91714287	4	91712702	91713745	CDS	ENSSSCG00000026371	OR6P1	protein_coding	olfactory receptor 6Y1
4	93179209	93179533	4	93176752	93179882	3'UTR	ENSSSCG00000006463	PEAR1	protein_coding	platelet endothelial aggregation receptor 1
4	93179209	93179533	4	93178857	93179882	3'UTR	ENSSSCG00000006463	PEAR1	protein_coding	platelet endothelial aggregation receptor 1
4	94013958	94014178	4	94013646	94013966	CDS	ENSSSCT00000047692			
4	94103231	94103869	4	94103297	94103376	CDS	ENSSSCG00000006504	KHDC4	protein_coding	KH domain containing 4%2C pre-mRNA splicing factor
4	97128471	97129305	4	97128371	97128508	CDS	ENSSSCG00000006605		protein_coding	repetin
4	97128471	97129305	4	97129296	97130410	CDS	ENSSSCG00000006605		protein_coding	repetin
4	97385931	97385953	4	97384718	97388804	5'UTR	ENSSSCT00000043021			
4	97487912	97488468	4	97488250	97488270	CDS	ENSSSCT00000036747			
4	97487912	97488468	4	97488250	97488270	CDS	ENSSSCT00000043943			
4	97526124	97526142	4	97525910	97526788	3'UTR	ENSSSCG00000006618	CELF3	protein_coding	CUGBP Elav-like family member 3
4	98388516	98388634	4	98388065	98389457	3'UTR	ENSSSCG00000006646	ARNT	protein_coding	aryl hydrocarbon receptor nuclear
4	98760695	98760899	4	98760547	98760767	CDS	ENSSSCG00000030119	PRPF3	protein_coding	pre-mRNA processing factor 3
4	102696359	102696552	4	102696341	102696500	CDS	ENSSSCG00000006726	SPAG17	protein_coding	sperm associated antigen 17
4	102754247	102754719	4	102754199	102754374	CDS	ENSSSCG00000006726	SPAG17	protein_coding	sperm associated antigen 17
4	103215994	103216205	4	103214939	103220723	3'UTR	ENSSSCG00000006730	MAN1A2	protein_coding	mannosidase alpha class 1A member 2
4	103977590	103977991	4	103977228	103977913	5'UTR	ENSSSCG00000006736	CD2	protein_coding	CD2 molecule
4	108825694	108826017	4	108825852	108825976	5'UTR	ENSSSCG00000006788	ADORA3	protein_coding	transmembrane and immunoglobulin domain containing 3 [Source:NCBI gene;Acc:104564308]
4	108825694	108826017	4	108825852	108825976	5'UTR	ENSSSCG00000006788	ADORA3	protein_coding	transmembrane and immunoglobulin domain containing 3
4	110005077	110005171	4	110005125	110005658	CDS	ENSSSCG00000028425	UBL4B	protein_coding	ubiquitin like 4B
4	111394702	111394720	4	111392338	111395777	3'UTR	ENSSSCG00000006850	FAM102B	protein_coding	family with sequence similarity 102 member
4	130579771	130580041	4	130579961	130580011	CDS	ENSSSCG00000040735	DDAH1	protein_coding	dimethylarginine dimethylaminohydrolase 1
4	130579771	130580041	4	130579706	130579960	5'UTR	ENSSSCG00000040735	DDAH1	protein_coding	dimethylarginine dimethylaminohydrolase 1
4	130649524	130649715	4	130649413	130650295	5'UTR	ENSSSCG00000025691	BCL10	protein_coding	BCL10 immune signaling adaptor
4	130749210	130749835	4	130747860	130749734	3'UTR	ENSSSCG00000006947	SYDE2	protein_coding	synapse defective Rho GTPase homolog 2
5	4107018	4107073	5	4105541	4110137	3'UTR	ENSSSCG00000031423	UPK3A	protein_coding	uroplakin 3A
5	11713749	11714414	5	11713910	11713960	CDS	ENSSSCG00000000152	RBFOX2	protein_coding	RNA binding fox-1 homolog 2
5	13359122	13359346	5	13359059	13359154	CDS	ENSSSCG00000000164	CRY1	protein_coding	cryptochrome circadian regulator 1
5	14974295	14974367	5	14973485	14975486	3'UTR	ENSSSCG00000032202		protein_coding	
5	14974295	14974367	5	14973487	14974603	3'UTR	ENSSSCG00000032202		protein_coding	
5	14974295	14974367	5	14973483	14976191	3'UTR	ENSSSCG00000031381	ARF3	protein_coding	ADP ribosylation factor 3
5	17828898	17829046	5	17829008	17830521	3'UTR	ENSSSCG00000000248	KRT5	protein_coding	keratin 6A
5	17828898	17829046	5	17829034	17830521	3'UTR	ENSSSCG00000000248	KRT5	protein_coding	keratin 6A
5	18015123	18015699	5	18015196	18015321	CDS	ENSSSCG00000000251	KRT1	protein_coding	keratin 1
5	18015123	18015699	5	18015453	18015617	CDS	ENSSSCG00000000251	KRT1	protein_coding	keratin 1
5	18417085	18417103	5	18415790	18418986	3'UTR	ENSSSCG00000036564	ZNF740	protein_coding	zinc finger protein 740
5	19269392	19269493	5	19267446	19270411	3'UTR	ENSSSCG00000038993	HOXC4	protein_coding	homeobox C4
5	19998727	19999596	5	19998898	19998951	CDS	ENSSSCG00000000299	TESPA1	protein_coding	thymocyte expressed%2C positive selection associated 1
5	19998727	19999596	5	19999574	19999623	CDS	ENSSSCG00000000299	TESPA1	protein_coding	thymocyte expressed%2C positive selection associated 1
5	21355400	21355470	5	21355251	21355481	3'UTR	ENSSSCG00000000370	DGKA	protein_coding	diacylglycerol kinase alpha
5	21355400	21355470	5	21355251	21355527	3'UTR	ENSSSCG00000000370	DGKA	protein_coding	diacylglycerol kinase alpha
5	21355400	21355470	5	21355251	21356183	3'UTR	ENSSSCG00000000370	DGKA	protein_coding	diacylglycerol kinase alpha
5	21355400	21355470	5	21355251	21355606	3'UTR	ENSSSCG00000000370	DGKA	protein_coding	diacylglycerol kinase alpha
5	21583261	21583841	5	21583664	21583749	CDS	ENSSSCG00000000382	SMARCC2	protein_coding	SWI/SNF related%2C matrix associated%2C actin dependent regulator of chromatin subfamily c member 2
5	22849794	22850564	5	22849732	22851971	3'UTR	ENSSSCG00000000439	KIF5A	protein_coding	kinesin family member 5A
5	22849794	22850564	5	22849732	22853212	3'UTR	ENSSSCG00000000439	KIF5A	protein_coding	kinesin family member 5A
5	22849794	22850564	5	22848595	22853212	3'UTR	ENSSSCG00000000439	KIF5A	protein_coding	kinesin family member 5A
5	22849794	22850564	5	22849732	22851970	3'UTR	ENSSSCG00000000439	KIF5A	protein_coding	kinesin family member 5A

5	22849794	22850564	5	22849732	22851973	3'UTR	ENSSSCG00000000439	KIF5A	protein_coding	kinesin family member 5A
5	26445818	26446361	5	26446239	26446391	CDS	ENSSSCG00000034686	TAFA2	protein_coding	TAFA chemokine like family member 2
5	33169312	33169574	5	33168646	33170451	3'UTR	ENSSSCG00000023630	CPM	protein_coding	carboxypeptidase M
5	48684323	48684865	5	48684792	48684818	5'UTR	ENSSSCG00000000563	LRMP	protein_coding	lymphoid restricted membrane protein
5	51824932	51825168	5	51824924	51825097	CDS	ENSSSCG00000000576	LDHB	protein_coding	lactate dehydrogenase B
5	54406562	54407283	5	54407101	54407997	CDS	ENSSSCG00000000589	CAPZA3	protein_coding	capping actin protein of muscle Z-line subunit alpha 3
5	54743171	54743683	5	54743268	54743390	CDS	ENSSSCG00000000591	PIK3C2G	protein_coding	phosphatidylinositol-4-phosphate 3-kinase catalytic subunit type 2 gamma
5	62835807	62836251	5	62835979	62836802	CDS	ENSSSCG00000000669	GDF3	protein_coding	growth differentiation factor 3
5	63669354	63669682	5	63667706	63670736	3'UTR	ENSSSCT00000064693			
5	63807116	63807298	5	63807090	63807185	CDS	ENSSSCG00000028373	ENO2	protein_coding	enolase 2
5	64331446	64331484	5	64331237	64331909	3'UTR	ENSSSCG00000000708	TNFRSF1A	protein_coding	TNF receptor superfamily member 1A
5	64331446	64331484	5	64331080	64332503	3'UTR	ENSSSCG00000000709	PLEKHG6	protein_coding	pleckstrin homology and RhoGEF domain containing G6
5	64331446	64331484	5	64331237	64335527	3'UTR	ENSSSCG00000000708	TNFRSF1A	protein_coding	TNF receptor superfamily member 1A
5	66028468	66028506	5	66028365	66028775	5'UTR	ENSSSCG00000039492	FGF23	protein_coding	fibroblast growth factor 23
5	67501581	67501622	5	67500789	67501915	CDS	ENSSSCG00000000748	IQSEC3	protein_coding	IQ motif and Sec7 domain 3
5	67852949	67853241	5	67852652	67853184	CDS	ENSSSCG00000033412	B4GALNT3	protein_coding	beta-1%2C4-N-acetyl-galactosaminyltransferase 3
5	67852949	67853241	5	67853224	67853450	CDS	ENSSSCG00000033412	B4GALNT3	protein_coding	beta-1%2C4-N-acetyl-galactosaminyltransferase 3
5	68143518	68143612	5	68139630	68144519	3'UTR	ENSSSCT00000046239			
5	68143518	68143612	5	68141514	68144519	3'UTR	ENSSSCT00000046779			
5	68143518	68143612	5	68142402	68144386	3'UTR	ENSSSCT00000050037			
5	68143518	68143612	5	68139630	68144519	3'UTR	ENSSSCT00000051733			
5	68143518	68143612	5	68139931	68143818	3'UTR	ENSSSCT00000062839			
5	75402872	75402908	5	75402498	75403615	3'UTR	ENSSSCG00000000801	TMEM117	protein_coding	transmembrane protein 117
5	80763584	80763836	5	80763554	80763631	CDS	ENSSSCG00000000854		protein_coding	stabilin 2
5	82853818	82853982	5	82853759	82853846	CDS	ENSSSCG00000000869	UTP20	protein_coding	UTP20 small subunit processome
5	83892098	83892551	5	83891209	83894636	3'UTR	ENSSSCG00000036721	DEPDC4	protein_coding	DEP domain containing 4
5	84669382	84669826	5	84669650	84670239	CDS	ENSSSCG00000022083	ANKS1B	protein_coding	ankyrin repeat and sterile alpha motif domain containing 1B [Source:NCBI gene;Acc:100513089]
5	84669382	84669826	5	84669650	84670239	CDS	ENSSSCT00000061480			
5	85316986	85317600	5	85317583	85319646	3'UTR	ENSSSCG00000038487	TMPO	protein_coding	thymopoietin
5	85316986	85317600	5	85317567	85319646	3'UTR	ENSSSCG00000038487	TMPO	protein_coding	thymopoietin
5	87253083	87253628	5	87253001	87253134	CDS	ENSSSCG00000028182	CDK17	protein_coding	cyclin dependent kinase 17
5	91885347	91885574	5	91885368	91885777	CDS	ENSSSCG00000039400	CCER1	protein_coding	coiled-coil glutamate rich protein 1
5	91885641	91885659	5	91885368	91885777	CDS	ENSSSCG00000039400	CCER1	protein_coding	coiled-coil glutamate rich protein 1
5	93019579	93020180	5	93019877	93019915	CDS	ENSSSCG00000027898	ATP2B1	protein_coding	ATPase plasma membrane Ca2+
5	97151097	97151362	5	97147620	97151922	3'UTR	ENSSSCG00000000936	SLC6A15	protein_coding	solute carrier family 6 member 15
5	100200541	100200866	5	100200729	100200966	CDS	ENSSSCG00000000940	PPFIA2	protein_coding	PTPRF interacting protein alpha 2
5	100760758	100761162	5	100760487	100763433	3'UTR	ENSSSCG00000026533	MYF6	protein_coding	myogenic factor 6
5	100782289	100782690	5	100782282	100782290	CDS	ENSSSCT00000043314			
5	101541481	101542290	5	101541822	101541952	CDS	ENSSSCG00000038973	PPP1R12A	protein_coding	protein phosphatase 1 regulatory subunit
5	102004270	102004823	5	102004668	102004835	CDS	ENSSSCG00000000948	SYT1	protein_coding	synaptotagmin 1
6	1670956	1671248	6	1671133	1671261	CDS	ENSSSCG00000002652	KLHDC4	protein_coding	kelch domain containing 4
6	14897629	14897805	6	14897673	14897693	CDS	ENSSSCT00000066411			
6	18314752	18315444	6	18315094	18316742	CDS	ENSSSCG00000032372	ZFP90	protein_coding	ZFP90 zinc finger protein
6	18314752	18315444	6	18312936	18315093	3'UTR	ENSSSCG00000032372	ZFP90	protein_coding	ZFP90 zinc finger protein
6	18314752	18315444	6	18315094	18316797	CDS	ENSSSCG00000032372	ZFP90	protein_coding	ZFP90 zinc finger protein
6	18314752	18315444	6	18312970	18315093	3'UTR	ENSSSCG00000032372	ZFP90	protein_coding	ZFP90 zinc finger protein
6	28667064	28667285	6	28667258	28667264	CDS	ENSSSCT00000048470			
6	28667064	28667285	6	28667015	28667257	5'UTR	ENSSSCT00000048470			
6	29418745	29418771	6	29417601	29418834	3'UTR	ENSSSCG00000034192	GNAO1	protein_coding	G protein subunit alpha o1
6	29883114	29883134	6	29880961	29884453	3'UTR	ENSSSCG00000002825		protein_coding	carboxylesterase 1
6	30511518	30512202	6	30511894	30512507	CDS	ENSSSCG00000032145	IRX5	protein_coding	iroquois homeobox 5
6	31051328	31051675	6	31048710	31051867	3'UTR	ENSSSCG00000002831	IRX3	protein_coding	iroquois homeobox 3
6	34604085	34604133	6	34603208	34605773	3'UTR	ENSSSCG00000040887	TENT4B	protein_coding	terminal nucleotidyltransferase 4B
6	44357500	44357548	6	44354236	44357540	3'UTR	ENSSSCG00000002877		protein_coding	zinc finger protein 181
6	45303451	45303543	6	45303447	45304553	5'UTR	ENSSSCG00000002917	NFKBID	protein_coding	NFKB inhibitor delta
6	46093631	46093952	6	46093205	46094409	3'UTR	ENSSSCG00000002935		protein_coding	zinc finger protein 568
6	46093631	46093952	6	46093204	46094409	3'UTR	ENSSSCG00000002935		protein_coding	zinc finger protein 568
6	48658548	48658900	6	48658784	48658967	CDS	ENSSSCG00000025109	PLD3	protein_coding	phospholipase D family member 3
6	48769708	48770333	6	48770317	48770519	CDS	ENSSSCG00000037494	SPTBN4	protein_coding	spectrin beta%2C non-erythrocytic 4
6	49265436	49265519	6	49265357	49265452	CDS	ENSSSCG00000038806	AXL	protein_coding	AXL receptor tyrosine kinase
6	49635121	49635451	6	49635108	49635949	5'UTR	ENSSSCT00000059568			
6	49635121	49635451	6	49634779	49635831	5'UTR	ENSSSCG00000003024	PRR19	protein_coding	proline rich 19
6	49825516	49825540	6	49825293	49825636	CDS	ENSSSCG00000037132	POU2F2	protein_coding	POU class 2 homeobox 2
6	49825516	49825540	6	49825333	49825784	3'UTR	ENSSSCG00000037132	POU2F2	protein_coding	POU class 2 homeobox 2
6	49825516	49825540	6	49825333	49825816	3'UTR	ENSSSCG00000037132	POU2F2	protein_coding	POU class 2 homeobox 2
6	50111277	50111311	6	50110507	50112056	3'UTR	ENSSSCG00000003011		protein_coding	carcinoembryonic antigen-related cell adhesion molecule 5-like [Source:NCBI gene;Acc:102158679]
6	50111277	50111311	6	50110507	50112056	3'UTR	ENSSSCT00000042086			
6	53076287	53076410	6	53076254	53076679	CDS	ENSSSCG00000024240	CCDC9	protein_coding	coiled-coil domain containing 9
6	53076287	53076410	6	53076254	53076647	CDS	ENSSSCG00000024240	CCDC9	protein_coding	coiled-coil domain containing 9
6	55022905	55023349	6	55021919	55023142	3'UTR	ENSSSCG00000037710	ZNF473	protein_coding	zinc finger protein 473
6	55022905	55023349	6	55021919	55023141	3'UTR	ENSSSCG00000037710	ZNF473	protein_coding	zinc finger protein 473

6	56096486	56096653	6	56094810	56096495	3'UTR	ENSSSCG00000033542	VSTM1	protein_coding	V-set and transmembrane domain-containing protein 1
6	56096486	56096653	6	56092621	56096495	3'UTR	ENSSSCG00000033542	VSTM1	protein_coding	V-set and transmembrane domain-containing protein 1
6	56096486	56096653	6	56092296	56096495	3'UTR	ENSSSCG00000033542	VSTM1	protein_coding	V-set and transmembrane domain-containing protein 1
6	58711214	58711239	6	58711123	58711294	CDS	ENSSSCG00000029231		protein_coding	leukocyte immunoglobulin-like receptor subfamily B member 3
6	58711214	58711239	6	58711172	58711294	CDS	ENSSSCG00000029231		protein_coding	leukocyte immunoglobulin-like receptor subfamily B member 3
6	58982850	58982863	6	58982794	58982916	CDS	ENSSSCT00000046487			
6	61982631	61982789	6	61982502	61983606	3'UTR	ENSSSCG00000035387	ZNF550	protein_coding	zinc finger protein 550-like
6	68551218	68551390	6	68549651	68553020	3'UTR	ENSSSCG00000040531		protein_coding	urotensin 2
6	71358416	71359028	6	71356269	71358671	3'UTR	ENSSSCG00000003414	ANGPTL7	protein_coding	angiopoietin like 7
6	71358416	71359028	6	71355721	71358671	3'UTR	ENSSSCG00000003414	ANGPTL7	protein_coding	angiopoietin like 7
6	73137725	73137959	6	73137757	73137765	CDS	ENSSSCG00000020710	PRDM2	protein_coding	PR/SET domain 2 [Source:NCBI gene;Acc:100518559]
6	79021761	79022259	6	79022021	79022990	3'UTR	ENSSSCG00000003512	EIF4G3	protein_coding	eukaryotic translation initiation factor 4
6	79021761	79022259	6	79022220	79022990	3'UTR	ENSSSCG00000003512	EIF4G3	protein_coding	eukaryotic translation initiation factor 4
6	80598850	80599066	6	80597180	80599440	3'UTR	ENSSSCG00000038706	C1QC	protein_coding	complement C1q C chain
6	84821814	84821962	6	84821640	84821859	CDS	ENSSSCG00000003578	FGR	protein_coding	FGR proto-oncogene%2C Src family tyrosine kinase
6	84821814	84821962	6	84821860	84821872	5'UTR	ENSSSCG00000003578	FGR	protein_coding	FGR proto-oncogene%2C Src family tyrosine kinase
6	88419668	88419834	6	88419453	88421259	3'UTR	ENSSSCG00000039885	PTP4A2	protein_coding	protein tyrosine phosphatase 4A2
6	88419668	88419834	6	88419387	88421887	3'UTR	ENSSSCG00000039885	PTP4A2	protein_coding	protein tyrosine phosphatase 4A2
6	89562520	89562522	6	89562405	89562541	CDS	ENSSSCG00000032224	A3GALT2	protein_coding	alpha 1%2C3-galactosyltransferase 2
6	91264259	91264697	6	91264532	91264682	CDS	ENSSSCG00000034618	ZMYM6	protein_coding	zinc finger MYM-type containing 6
6	92129978	92130704	6	92130128	92130264	CDS	ENSSSCG00000003630	AGO3	protein_coding	argonaute RISC component 1
6	93597234	93597262	6	93597059	93598295	3'UTR	ENSSSCG00000029991	SNIP1	protein_coding	Smad nuclear interacting protein 1
6	96062750	96063011	6	96062785	96065860	3'UTR	ENSSSCG00000023322		protein_coding	collagen type IX alpha 2 chain
6	96881164	96881686	6	96881126	96881299	CDS	ENSSSCG00000031730		protein_coding	proteasome assembly chaperone 2
6	108329111	108329646	6	108329570	108329641	CDS	ENSSSCG00000040452	TMEM241	protein_coding	transmembrane protein 241
6	108329111	108329646	6	108329642	108329730	5'UTR	ENSSSCG00000040452	TMEM241	protein_coding	transmembrane protein 241
6	108329111	108329646	6	108329642	108329763	5'UTR	ENSSSCG00000040452	TMEM241	protein_coding	transmembrane protein 241
6	111147435	111147858	6	111146103	111147914	CDS	ENSSSCG00000003719		protein_coding	potassium channel tetramerization domain containing 1
6	115453115	115453683	6	115452062	115454339	3'UTR	ENSSSCT00000025628			
6	123594847	123594922	6	123594858	123594885	CDS	ENSSSCT00000062627			
6	131918372	131918412	6	131917786	131918375	5'UTR	ENSSSCG00000003761	ADGRL2	protein_coding	adhesion G protein-coupled receptor L2
6	131918372	131918412	6	131917594	131918389	5'UTR	ENSSSCG00000003761	ADGRL2	protein_coding	adhesion G protein-coupled receptor L2
6	131918372	131918412	6	131917604	131918383	5'UTR	ENSSSCG00000003761	ADGRL2	protein_coding	adhesion G protein-coupled receptor L2
6	131918372	131918412	6	131917594	131918393	5'UTR	ENSSSCG00000003761	ADGRL2	protein_coding	adhesion G protein-coupled receptor L2
6	134424403	134424664	6	134424360	134424543	CDS	ENSSSCG00000003762	ADGRL4	protein_coding	adhesion G protein-coupled receptor L4
6	134729939	134729961	6	134727342	134730978	3'UTR	ENSSSCG00000039548	PTGFR	protein_coding	prostaglandin F receptor
6	136221220	136221470	6	136221203	136221310	CDS	ENSSSCG00000033425	ST6GALNAC5	protein_coding	ST6 N-acetylgalactosaminide alpha-2%2C6-sialyltransferase 5
6	138613029	138613205	6	138611828	138613063	3'UTR	ENSSSCG00000003781		protein_coding	glutamate rich 3
6	146866459	146866801	6	146866731	146866770	CDS	ENSSSCG00000025188	LEPR	protein_coding	leptin receptor
6	146866459	146866801	6	146866771	146866785	5'UTR	ENSSSCG00000025188	LEPR	protein_coding	leptin receptor
6	146866459	146866801	6	146866771	146866806	5'UTR	ENSSSCG00000025188	LEPR	protein_coding	leptin receptor
6	146979743	146980171	6	146980070	146980256	CDS	ENSSSCG00000003806	LEPROT	protein_coding	leptin receptor overlapping transcript
6	147177808	147177864	6	147177034	147182943	3'UTR	ENSSSCG00000040337	AK4	protein_coding	adenylate kinase 4
6	147177808	147177864	6	147177031	147182943	3'UTR	ENSSSCG00000040337	AK4	protein_coding	adenylate kinase 4
6	147177808	147177864	6	147177032	147178479	3'UTR	ENSSSCG00000040337	AK4	protein_coding	adenylate kinase 4
6	148982625	148983220	6	148982846	148982932	CDS	ENSSSCG00000003814	EFCAB7	protein_coding	EF-hand calcium binding domain 7
6	152476754	152477470	6	152477244	152477406	CDS	ENSSSCG00000003825	CYP2J34	protein_coding	cytochrome P450 2J2
6	160616158	160616873	6	160616153	160616330	CDS	ENSSSCT00000004283			
6	164517834	164518066	6	164517333	164518300	3'UTR	ENSSSCG00000003891	CYP4A24	protein_coding	cytochrome P450 4A24
6	164536770	164536982	6	164536959	164537084	CDS	ENSSSCT00000062187			
6	165222241	165222454	6	165222114	165226578	3'UTR	ENSSSCG00000029145	POMGNT1	protein_coding	protein O-linked mannose N-acetylglucosaminyltransferase 1 (beta
6	165222241	165222454	6	165221824	165226578	3'UTR	ENSSSCG00000029145	POMGNT1	protein_coding	protein O-linked mannose N-acetylglucosaminyltransferase 1 (beta
6	166325040	166325076	6	166324872	166327314	3'UTR	ENSSSCG00000003922	UROD	protein_coding	uroporphyrinogen decarboxylase
6	166325040	166325076	6	166324065	166325995	3'UTR	ENSSSCG00000003921	ZSWIM5	protein_coding	zinc finger SWIM-type containing 5
7	1570116	1570158	7	1567818	1571657	3'UTR	ENSSSCG00000028777	MYLK4	protein_coding	myosin light chain kinase family member 4
7	4752553	4753358	7	4752682	4752877	CDS	ENSSSCG00000001024	RIOK1	protein_coding	RIO kinase 1
7	8126717	8126806	7	8124558	8127427	3'UTR	ENSSSCG00000037420	TMEM170B	protein_coding	transmembrane protein 170B
7	9740953	9741625	7	9735935	9742386	3'UTR	ENSSSCG00000035867	GFOD1	protein_coding	glucose-fructose oxidoreductase domain containing 1
7	11600487	11600788	7	11600307	11601399	3'UTR	ENSSSCG00000001061	JARID2	protein_coding	jumonji and AT-rich interaction domain containing 2
7	11600487	11600788	7	11600307	11602097	3'UTR	ENSSSCG00000001061	JARID2	protein_coding	jumonji and AT-rich interaction domain containing 2
7	12556196	12556534	7	12556288	12556417	5'UTR	ENSSSCG00000001065	ATXN1	protein_coding	ataxin 1
7	13498273	13498327	7	13498222	13499877	3'UTR	ENSSSCG00000020858	KIF13A	protein_coding	kinesin family member 13A
7	14118327	14118369	7	14115547	14119371	3'UTR	ENSSSCG00000001076	RNF144B	protein_coding	ring finger protein 144B
7	14118327	14118369	7	14115547	14119091	3'UTR	ENSSSCG00000001076	RNF144B	protein_coding	ring finger protein 144B
7	14118327	14118369	7	14115547	14119092	3'UTR	ENSSSCG00000001076	RNF144B	protein_coding	ring finger protein 144B

7	14118327	14118369	7	14115547	14119430	3'UTR	ENSSSCG00000001076	RNF144B	protein_coding	ring finger protein 144B
7	19917304	19917836	7	19917558	19917761	CDS	ENSSSCG00000001099	CMAH	protein_coding	cytidine monophosphate-N-acetylneuraminic acid hydroxylase
7	20903648	20904215	7	20902131	20904000	3'UTR	ENSSSCT00000038922			
7	21947575	21947742	7	21944927	21949794	3'UTR	ENSSSCG00000001197	ZNF165	protein_coding	zinc finger protein 165
7	22528466	22528734	7	22527596	22528531	CDS	ENSSSCG00000031213	OLF42	protein_coding	Sus scrofa olfactory receptor-like protein 42-2 (OLF42-2)%2C mRNA.
7	23211557	23211581	7	23209172	23213943	3'UTR	ENSSSCG00000031492	PPP1R18	protein_coding	protein phosphatase 1 regulatory subunit 18
7	23211557	23211581	7	23209172	23212674	3'UTR	ENSSSCG00000031492	PPP1R18	protein_coding	protein phosphatase 1 regulatory subunit 18
7	23634887	23635255	7	23632678	23635383	3'UTR	ENSSSCG00000001398		protein_coding	MHC class I antigen 7
7	23634887	23635255	7	23632678	23636117	3'UTR	ENSSSCG00000001398		protein_coding	MHC class I antigen 7
7	24285443	24285839	7	24285625	24285651	CDS	ENSSSCG00000001441		protein_coding	butyrophilin-like protein 1
7	29713551	29713588	7	29713538	29714110	3'UTR	ENSSSCT00000024368			
7	29713551	29713588	7	29713538	29713685	3'UTR	ENSSSCT00000039374			
7	29713551	29713588	7	29713538	29713713	3'UTR	ENSSSCT00000040002			
7	29713551	29713588	7	29710681	29714110	3'UTR	ENSSSCT00000040284			
7	37921005	37921621	7	37920996	37921057	CDS	ENSSSCG00000001646	BICRAL	protein_coding	BRD4 interacting chromatin remodeling complex associated protein like
7	38623381	38623419	7	38622290	38623835	3'UTR	ENSSSCG00000016346	MAD2L1BP	protein_coding	MAD2L1 binding protein
7	38623381	38623419	7	38622290	38623832	3'UTR	ENSSSCG00000016346	MAD2L1BP	protein_coding	MAD2L1 binding protein
7	40908327	40908925	7	40908523	40908693	CDS	ENSSSCG00000025788	ENPP4	protein_coding	ectonucleotide pyrophosphatase/phosphodiesterase 4
7	40910147	40910215	7	40909394	40913605	3'UTR	ENSSSCG00000025788	ENPP4	protein_coding	ectonucleotide pyrophosphatase/phosphodiesterase 4
7	41456450	41456791	7	41453317	41457879	3'UTR	ENSSSCG00000001720	SLC25A27	protein_coding	solute carrier family 25 member 27
7	41456450	41456791	7	41453317	41457884	3'UTR	ENSSSCG00000001720	SLC25A27	protein_coding	solute carrier family 25 member 27
7	42400358	42400531	7	42398972	42403213	3'UTR	ENSSSCG00000022089	ADGRF4	protein_coding	adhesion G protein-coupled receptor F2 [Source:NCBI gene;Acc:100621059]
7	42400358	42400531	7	42398972	42403213	3'UTR	ENSSSCT00000023296			
7	50149248	50149266	7	50149248	50149388	CDS	ENSSSCT00000045449			
7	58162303	58162417	7	58161883	58163197	3'UTR	ENSSSCG00000001878	PTPN9	protein_coding	protein tyrosine phosphatase non-receptor
7	65253712	65253867	7	65253707	65253820	CDS	ENSSSCG00000001960	EAPP	protein_coding	E2F associated phosphoprotein
7	74855453	74856052	7	74852649	74856941	3'UTR	ENSSSCG00000001984	KHNYN	protein_coding	KH and NYN domain containing
7	74855453	74856052	7	74854214	74856941	3'UTR	ENSSSCG00000001984	KHNYN	protein_coding	KH and NYN domain containing
7	74882388	74882414	7	74879726	74882609	CDS	ENSSSCG00000032473	NYNRIN	protein_coding	NYN domain and retroviral integrase
7	76059381	76059596	7	76058792	76059798	3'UTR	ENSSSCT00000057033			
7	76059381	76059596	7	76058792	76059798	3'UTR	ENSSSCT00000057640			
7	77312960	77312978	7	77310953	77313682	3'UTR	ENSSSCG00000041136		protein_coding	
7	77367670	77367814	7	77367544	77368022	5'UTR	ENSSSCG00000038885		protein_coding	
7	84758176	84758316	7	84757040	84758473	3'UTR	ENSSSCT00000060858			
7	90218831	90219373	7	90218945	90219022	CDS	ENSSSCT00000049863			
7	90218831	90219373	7	90219038	90219124	CDS	ENSSSCT00000049863			
7	109950660	109951296	7	109950721	109950834	CDS	ENSSSCG00000027865	GALC	protein_coding	galactosylceramidase
7	109950660	109951296	7	109950721	109950814	CDS	ENSSSCG00000027865	GALC	protein_coding	galactosylceramidase
7	109950660	109951296	7	109950815	109950834	5'UTR	ENSSSCG00000027865	GALC	protein_coding	galactosylceramidase
7	111037591	111038053	7	111036504	111042787	3'UTR	ENSSSCT00000056692			
7	113510835	113511479	7	113511145	113511200	CDS	ENSSSCG00000002444	FBLN5	protein_coding	fibulin 5
7	113510835	113511479	7	113511201	113511491	5'UTR	ENSSSCG00000002444	FBLN5	protein_coding	fibulin 5
7	114354899	114354985	7	114354872	114355362	CDS	ENSSSCG00000002456	CHGA	protein_coding	chromogranin A
7	114355002	114355326	7	114354872	114355362	CDS	ENSSSCG00000002456	CHGA	protein_coding	chromogranin A
7	117534430	117534926	7	117534750	117534976	CDS	ENSSSCG00000002502	ATG2B	protein_coding	autophagy related 2B
7	117577785	117578513	7	117578320	117578482	CDS	ENSSSCG00000002502	ATG2B	protein_coding	autophagy related 2B
7	120886674	120886722	7	120885789	120887175	3'UTR	ENSSSCG00000027667	EML1	protein_coding	EMAP like 1
8	1922045	1922067	8	1921986	1922113	CDS	ENSSSCG00000008697	HTT	protein_coding	huntingtin
8	6410713	6410953	8	6409441	6414192	3'UTR	ENSSSCG00000036501	WDR1	protein_coding	WD repeat domain 1
8	9194982	9195032	8	9194887	9198309	3'UTR	ENSSSCG00000008735	BOD1L1	protein_coding	biorientation of chromosomes in cell division 1 like 1
8	11274751	11275019	8	11274664	11274869	CDS	ENSSSCG00000008745	PROM1	protein_coding	prominin 1
8	19133010	19133408	8	19133120	19133238	CDS	ENSSSCG00000025315	SEPSECS	protein_coding	Sep (O-phosphoserine) tRNA:Sec (selenocysteine) tRNA synthase
8	28312280	28312540	8	28308274	28313150	3'UTR	ENSSSCT00000009594			
8	29120645	29120886	8	29118512	29121254	3'UTR	ENSSSCG00000008772	RELL1	protein_coding	RELT like 1
8	29136998	29137210	8	29136919	29137057	CDS	ENSSSCG00000008772	RELL1	protein_coding	RELT like 1
8	29589726	29589762	8	29588911	29590571	3'UTR	ENSSSCG00000028983	TBC1D1	protein_coding	TBC1 domain family member 1
8	29589726	29589762	8	29588911	29590569	3'UTR	ENSSSCG00000028983	TBC1D1	protein_coding	TBC1 domain family member 1
8	33088715	33089695	8	33089133	33089235	CDS	ENSSSCG00000008801	SLC30A9	protein_coding	solute carrier family 30 member 9
8	39008706	39009290	8	39008744	39008783	CDS	ENSSSCG00000008831	DCUN1D4	protein_coding	defective in cullin neddylation 1 domain containing 4
8	39706168	39706657	8	39706654	39707124	CDS	ENSSSCG00000008835	RASL11B	protein_coding	RAS like family 11 member B
8	41839874	41840425	8	41840259	41840415	CDS	ENSSSCG00000008844	KDR	protein_coding	kinase insert domain receptor
8	47473641	47473709	8	47473062	47473893	5'UTR	ENSSSCG00000008875	RXFP1	protein_coding	relaxin family peptide receptor 1
8	63332522	63333139	8	63332795	63332980	CDS	ENSSSCG00000008919	EPHA5	protein_coding	EPH receptor A5
8	65636919	65637259	8	65636850	65637500	3'UTR	ENSSSCG00000008927	TMPRSS11A	protein_coding	transmembrane protease serine 11A
8	66098884	66099068	8	66098764	66099202	CDS	ENSSSCG00000035505	YTHDC1	protein_coding	YTH domain containing 1
8	66148576	66149147	8	66148863	66148950	CDS	ENSSSCG00000037635		protein_coding	
8	66148576	66149147	8	66148986	66149125	CDS	ENSSSCG00000037635		protein_coding	
8	66748841	66749415	8	66748607	66748873	5'UTR	ENSSSCG00000032374	SULT1B1	protein_coding	sulfotransferase family cytosolic 1B
8	66752994	66753348	8	66753235	66753278	CDS	ENSSSCT00000049760			

8	70285147	70285718	8	70283900	70286876	5'UTR	ENSSSCG00000008961	MTHFD2L	protein_coding	methylenetetrahydrofolate dehydrogenase (NADP+ dependent) 2 like [Source:NCBI gene;Acc:100525706]
8	70285147	70285718	8	70285640	70285752	CDS	ENSSSCG00000008961	MTHFD2L	protein_coding	methylenetetrahydrofolate dehydrogenase (NADP+ dependent) 2 like
8	77328693	77329302	8	77328699	77329562	3'UTR	ENSSSCG00000020717	FAM160A1	protein_coding	family with sequence similarity 160 member
8	77328693	77329302	8	77328622	77329562	3'UTR	ENSSSCG00000020717	FAM160A1	protein_coding	family with sequence similarity 160 member
8	77340427	77340513	8	77340290	77341727	3'UTR	ENSSSCG00000020717	FAM160A1	protein_coding	family with sequence similarity 160 member
8	81220101	81220513	8	81220484	81220636	CDS	ENSSSCG00000009031	EDNRA	protein_coding	endothelin receptor type A
8	84286587	84287167	8	84286542	84286700	CDS	ENSSSCG00000009048	GAB1	protein_coding	GRB2 associated binding protein 1
8	84286587	84287167	8	84286536	84286700	CDS	ENSSSCG00000009048	GAB1	protein_coding	GRB2 associated binding protein 1
8	100857357	100858264	8	100857917	100858876	CDS	ENSSSCG00000009083	SPRY1	protein_coding	sprouty RTK signaling antagonist 1
8	100857357	100858264	8	100856695	100857916	3'UTR	ENSSSCG00000009083	SPRY1	protein_coding	sprouty RTK signaling antagonist 1
8	100857357	100858264	8	100854658	100857916	3'UTR	ENSSSCG00000009083	SPRY1	protein_coding	sprouty RTK signaling antagonist 1
8	103298549	103299143	8	103298468	103298625	CDS	ENSSSCG00000009101	PRDM5	protein_coding	PR/SET domain 5
8	108027703	108028392	8	108028248	108028433	CDS	ENSSSCG00000033834	NDST4	protein_coding	bifunctional heparan sulfate N-deacetylase/N-sulfotransferase 4
8	108234842	108235016	8	108233520	108235399	3'UTR	ENSSSCG00000031904	UGT8	protein_coding	UDP glycosyltransferase 8
8	108234842	108235016	8	108230635	108235399	3'UTR	ENSSSCG00000031904	UGT8	protein_coding	UDP glycosyltransferase 8
8	108234842	108235016	8	108232640	108235399	3'UTR	ENSSSCG00000031904	UGT8	protein_coding	UDP glycosyltransferase 8
8	112186283	112186725	8	112186137	112190071	3'UTR	ENSSSCG00000036236	ELOVL6	protein_coding	ELOVL fatty acid elongase 6
8	112186283	112186725	8	112186137	112190072	3'UTR	ENSSSCG00000036236	ELOVL6	protein_coding	ELOVL fatty acid elongase 6
8	112186283	112186725	8	112186137	112191298	3'UTR	ENSSSCG00000036236	ELOVL6	protein_coding	ELOVL fatty acid elongase 6
8	112186283	112186725	8	112186137	112191299	3'UTR	ENSSSCG00000036236	ELOVL6	protein_coding	ELOVL fatty acid elongase 6
8	113233473	113233677	8	113233515	113233541	CDS	ENSSSCG00000039813	COL25A1	protein_coding	collagen type XXV alpha 1 chain
8	114024629	114024831	8	114024734	114025369	CDS	ENSSSCG00000009151	CYP2U1	protein_coding	cytochrome P450 2U1
8	115434308	115435335	8	115434915	115437530	3'UTR	ENSSSCG00000024168	AIMP1	protein_coding	aminoacyl tRNA synthetase complex interacting multifunctional protein 1
8	115434308	115435335	8	115432088	115437530	3'UTR	ENSSSCG00000024168	AIMP1	protein_coding	aminoacyl tRNA synthetase complex interacting multifunctional protein 1
8	115698657	115699056	8	115698163	115700902	3'UTR	ENSSSCG00000021784	TBCK	protein_coding	TBC1 domain containing kinase
8	115823070	115823359	8	115822997	115823089	5'UTR	ENSSSCG00000038801	NPNT	protein_coding	nephronectin
8	117994395	117994773	8	117993403	117995216	3'UTR	ENSSSCG00000022282	BDH2	protein_coding	3-hydroxybutyrate dehydrogenase 2
8	118406378	118406828	8	118406433	118406547	CDS	ENSSSCG00000030957	NFKB1	protein_coding	nuclear factor kappa B subunit 1
8	118859606	118860184	8	118859724	118860026	CDS	ENSSSCG00000022361	BANK1	protein_coding	B cell scaffold protein with ankyrin repeats
8	119640503	119641168	8	119640667	119640679	CDS	ENSSSCT00000061221			
8	131085439	131085889	8	131085181	131085759	5'UTR	ENSSSCG00000009216	SPP1	protein_coding	secreted phosphoprotein 1
8	131290022	131290380	8	131290275	131290322	CDS	ENSSSCG00000009220	DMP1	protein_coding	dentin matrix acidic phosphoprotein 1
8	136880096	136880315	8	136878960	136880859	3'UTR	ENSSSCG00000029061	BMP3	protein_coding	bone morphogenetic protein 3
8	137605098	137605388	8	137603847	137605599	3'UTR	ENSSSCG00000036555	PRDM8	protein_coding	PR/SET domain 8
9	7305572	7305767	9	7305540	7305657	CDS	ENSSSCG00000014818	STARD10	protein_coding	StAR related lipid transfer domain
9	18081499	18081945	9	18081876	18082020	CDS	ENSSSCG00000014904	DLG2	protein_coding	discs large MAGUK scaffold protein 2
9	20623013	20623468	9	20622961	20624814	3'UTR	ENSSSCG00000014921	PRSS23	protein_coding	serine protease 23
9	20623013	20623468	9	20622961	20625139	3'UTR	ENSSSCG00000014921	PRSS23	protein_coding	serine protease 23
9	26548495	26548541	9	26546473	26549286	3'UTR	ENSSSCG00000022490	GPR83	protein_coding	G protein-coupled receptor 83
9	28400266	28400516	9	28400359	28400554	CDS	ENSSSCG00000014972	CCDC82	protein_coding	coiled-coil domain containing 82
9	28400266	28400516	9	28400213	28400554	CDS	ENSSSCG00000014972	CCDC82	protein_coding	coiled-coil domain containing 82
9	31158725	31159089	9	31159007	31159056	CDS	ENSSSCT00000055258			
9	35070423	35070683	9	35070382	35070437	CDS	ENSSSCG00000032358		protein_coding	
9	36306357	36306584	9	36306319	36306494	CDS	ENSSSCG00000015001	SLC35F2	protein_coding	solute carrier family 35 member F2
9	37022425	37022821	9	37022527	37022653	CDS	ENSSSCG00000015011		protein_coding	DEAD-box helicase 10
9	40945484	40945524	9	40945476	40947749	3'UTR	ENSSSCG00000015045	NCAM1	protein_coding	neural cell adhesion molecule 1
9	40957616	40958052	9	40957516	40957845	3'UTR	ENSSSCG00000015045	NCAM1	protein_coding	neural cell adhesion molecule 1
9	40957616	40958052	9	40957516	40960580	3'UTR	ENSSSCG00000015045	NCAM1	protein_coding	neural cell adhesion molecule 1
9	40957616	40958052	9	40957516	40960597	3'UTR	ENSSSCG00000015045	NCAM1	protein_coding	neural cell adhesion molecule 1
9	40957616	40958052	9	40957516	40960966	3'UTR	ENSSSCG00000015045	NCAM1	protein_coding	neural cell adhesion molecule 1
9	41067667	41067711	9	41067491	41069869	3'UTR	ENSSSCG00000015048	DRD2	protein_coding	dopamine receptor D2
9	45679796	45680024	9	45679630	45679963	CDS	ENSSSCG00000046892	ATP5MG	protein_coding	ATP synthase membrane subunit g [Source:NCBI gene;Acc:396956]
9	59040743	59041152	9	59037716	59042877	3'UTR	ENSSSCG00000037534	OPCML	protein_coding	opioid binding protein/cell adhesion
9	61135124	61135735	9	61135549	61135670	CDS	ENSSSCT00000056360			
9	61135124	61135735	9	61135703	61135797	CDS	ENSSSCT00000056360			
9	64206707	64206785	9	64204591	64207001	3'UTR	ENSSSCG00000015271	PRELP	protein_coding	proline and arginine rich end leucine rich repeat protein
9	66652707	66652888	9	66652705	66652831	CDS	ENSSSCG00000036352	RAB7B	protein_coding	RAB7B%2C member RAS oncogene family
9	67114593	67114812	9	67114344	67117314	3'UTR	ENSSSCG00000015645		protein_coding	SLIT-ROBO Rho GTPase activating protein
9	67114593	67114812	9	67114344	67117315	3'UTR	ENSSSCG00000015645		protein_coding	SLIT-ROBO Rho GTPase activating protein
9	67725682	67725960	9	67725574	67725717	CDS	ENSSSCG00000015661	C4BPB	protein_coding	complement component 4 binding protein
9	67725682	67725960	9	67725718	67725781	3'UTR	ENSSSCG00000015661	C4BPB	protein_coding	complement component 4 binding protein
9	67725682	67725960	9	67725574	67726357	5'UTR	ENSSSCG00000015662	C4BPA	protein_coding	complement component 4 binding protein%2C alpha
9	67725682	67725960	9	67725718	67725782	3'UTR	ENSSSCG00000015661	C4BPB	protein_coding	complement component 4 binding protein
9	67725682	67725960	9	67725718	67725779	3'UTR	ENSSSCG00000015661	C4BPB	protein_coding	complement component 4 binding protein
9	68547537	68547789	9	68544721	68549293	3'UTR	ENSSSCG00000015299	STEAP4	protein_coding	STEAP4 metalloreductase
9	72427061	72427381	9	72427026	72427149	CDS	ENSSSCG00000029967	PEX1	protein_coding	peroxisomal biogenesis factor 1
9	87766392	87766889	9	87766382	87766503	CDS	ENSSSCG00000015368	HDAC9	protein_coding	histone deacetylase 9
9	92629522	92630218	9	92629747	92629797	CDS	ENSSSCT00000030941			
9	98437287	98437323	9	98437087	98437992	5'UTR	ENSSSCG00000015403	HGF	protein_coding	hepatocyte growth factor
9	102364106	102364546	9	102363944	102367122	3'UTR	ENSSSCG00000038977	RSBN1L	protein_coding	round spermatid basic protein 1 like
9	102810302	102810705	9	102810378	102810605	CDS	ENSSSCG00000015412	CCDC146	protein_coding	coiled-coil domain containing 146

9	102816320	102816551	9	102816291	102816348	CDS	ENSSSCG00000015412	CCDC146	protein_coding	coiled-coil domain containing 146
9	103160068	103160802	9	103160355	103160445	CDS	ENSSSCG00000037822	FBXL13	protein_coding	F-box and leucine rich repeat protein 13
9	105377691	105377829	9	105377668	105378115	CDS	ENSSSCG00000015428	PUS7	protein_coding	pseudouridine synthase 7
9	107805976	107806337	9	107806319	107806398	CDS	ENSSSCT00000050639			
9	109267189	109267402	9	109267356	109267539	CDS	ENSSSCG00000015453	PDIA4	protein_coding	protein disulfide isomerase family A
9	114086945	114087263	9	114083232	114087942	3'UTR	ENSSSCG00000035066	MYOC	protein_coding	myocilin
9	114785401	114785758	9	114785607	114785632	CDS	ENSSSCT00000048945			
9	116261165	116261420	9	116261130	116261360	CDS	ENSSSCG00000015498	RC3H1	protein_coding	ring finger and CCCH-type domains 1
9	116261165	116261420	9	116261361	116261527	5'UTR	ENSSSCG00000015498	RC3H1	protein_coding	ring finger and CCCH-type domains 1
9	117058628	117058726	9	117055061	117060053	3'UTR	ENSSSCG00000015499	RABGAP1L	protein_coding	RAB GTPase activating protein 1 like
9	118500313	118500865	9	118500640	118500833	CDS	ENSSSCG00000015512	PAPPA2	protein_coding	pappalysin 2
9	119820386	119820888	9	119820634	119820929	CDS	ENSSSCG00000039332	SEC16B	protein_coding	SEC16 homolog B%2C endoplasmic reticulum export factor
9	123859211	123859877	9	123859234	123859351	CDS	ENSSSCG00000015548	RGSL1	protein_coding	regulator of G protein signaling like 1
9	123955569	123955818	9	123953292	123958165	3'UTR	ENSSSCG00000039986	RGS8	protein_coding	regulator of G protein signaling 8
9	123955569	123955818	9	123953294	123958165	3'UTR	ENSSSCG00000039986	RGS8	protein_coding	regulator of G protein signaling 8
9	123955569	123955818	9	123953291	123958165	3'UTR	ENSSSCG00000039986	RGS8	protein_coding	regulator of G protein signaling 8
9	123955569	123955818	9	123953068	123958165	3'UTR	ENSSSCG00000039986	RGS8	protein_coding	regulator of G protein signaling 8
9	125183020	125183294	9	125183035	125183111	CDS	ENSSSCG00000030217	COLGALT2	protein_coding	collagen beta(1-O)galactosyltransferase 2
9	125910874	125911636	9	125908864	125912326	3'UTR	ENSSSCG00000015566	EDEM3	protein_coding	ER degradation enhancing alpha-mannosidase like protein 3
9	125910874	125911636	9	125908842	125912326	3'UTR	ENSSSCG00000015566	EDEM3	protein_coding	ER degradation enhancing alpha-mannosidase like protein 3
9	127540684	127540729	9	127540310	127540790	5'UTR	ENSSSCG00000015576	TPR	protein_coding	translocated promoter region%2C nuclear basket protein
9	127540684	127540729	9	127540206	127540790	5'UTR	ENSSSCG00000015576	TPR	protein_coding	translocated promoter region%2C nuclear basket protein
9	129578223	129578390	9	129577933	129578347	5'UTR	ENSSSCT00000061770			
9	130285056	130285558	9	130285243	130285332	CDS	ENSSSCG00000015586	RPS6KC1	protein_coding	ribosomal protein S6 kinase C1
9	130285056	130285558	9	130285551	130287190	CDS	ENSSSCG00000015586	RPS6KC1	protein_coding	ribosomal protein S6 kinase C1
9	130285056	130285558	9	130285243	130285550	3'UTR	ENSSSCG00000015586	RPS6KC1	protein_coding	ribosomal protein S6 kinase C1
9	131705152	131705275	9	131704683	131705539	5'UTR	ENSSSCG00000035369	RD3	protein_coding	retinal degeneration 3%2C GUCY2D
9	132739303	132739473	9	132739060	132739818	CDS	ENSSSCG00000026404	SERTAD4	protein_coding	SERTA domain containing 4
9	133139417	133139447	9	133139295	133139550	CDS	ENSSSCG00000015611	UTP25	protein_coding	UTP25 small subunit processor component
9	133139417	133139447	9	133139371	133140272	5'UTR	ENSSSCG00000015611	UTP25	protein_coding	UTP25 small subunit processor component
9	133231398	133231434	9	133229659	133231863	3'UTR	ENSSSCT00000045066			
9	133231398	133231434	9	133229659	133231863	3'UTR	ENSSSCT00000063087			
9	136235771	136236041	9	136235785	136235908	CDS	ENSSSCG00000015628	SPATA48	protein_coding	spermatogenesis associated 48
10	6126821	6127220	10	6126921	6127549	CDS	ENSSSCG00000038066	USH2A	protein_coding	usherin
10	9724282	9724705	10	9724290	9724392	CDS	ENSSSCG00000010823	IARS2	protein_coding	isoleucyl-tRNA synthetase 2%2C
10	12482517	12483315	10	12483069	12483126	CDS	ENSSSCG00000025686	KMO	protein_coding	kynurenine 3-monooxygenase
10	12486393	12486628	10	12486373	12486455	CDS	ENSSSCG00000025686	KMO	protein_coding	kynurenine 3-monooxygenase
10	21024436	21024917	10	21024398	21024482	CDS	ENSSSCG00000010904	NEK7	protein_coding	NIMA related kinase 7
10	21024436	21024917	10	21024426	21024482	CDS	ENSSSCG00000010904	NEK7	protein_coding	NIMA related kinase 7
10	25386717	25386868	10	25386710	25386805	CDS	ENSSSCG00000011168	ZNF510	protein_coding	zinc finger protein 510
10	30730161	30730187	10	30728839	30731240	3'UTR	ENSSSCG00000010960	SLC28A3	protein_coding	solute carrier family 28 member 3
10	32505898	32506492	10	32506308	32506930	CDS	ENSSSCG00000010984	KIF24	protein_coding	kinesin family member 24
10	32505898	32506492	10	32506292	32506307	5'UTR	ENSSSCG00000010984	KIF24	protein_coding	kinesin family member 24
10	38880129	38880408	10	38880201	38880296	CDS	ENSSSCG00000026552	MFSD14B	protein_coding	major facilitator superfamily domain containing 14B
10	40578343	40578580	10	40578558	40578667	CDS	ENSSSCG00000035848		protein_coding	
10	42518403	42518840	10	42518411	42518674	CDS	ENSSSCG00000011026	ARHGAP12	protein_coding	Rho GTPase activating protein 12
10	42595290	42595351	10	42595285	42595306	3'UTR	ENSSSCG00000011027	KIF5B	protein_coding	kinesin family member 5B
10	45363878	45364580	10	45363929	45364038	CDS	ENSSSCG00000022953	PTER	protein_coding	phosphotriesterase related
10	45363878	45364580	10	45361713	45363928	3'UTR	ENSSSCG00000022953	PTER	protein_coding	phosphotriesterase related
10	46266757	46267151	10	46267135	46267240	CDS	ENSSSCG00000011046	ITGA8	protein_coding	integrin subunit alpha 8
10	46600390	46600820	10	46599807	46601075	3'UTR	ENSSSCG00000011047	FAM171A1	protein_coding	family with sequence similarity 171 member
10	46600390	46600820	10	46599807	46601078	3'UTR	ENSSSCG00000011047	FAM171A1	protein_coding	family with sequence similarity 171 member
10	46791920	46792145	10	46791834	46791962	CDS	ENSSSCG00000034119	MEIG1	protein_coding	meiosis/spermiogenesis associated 1
10	47688689	47688836	10	47688827	47688880	5'UTR	ENSSSCG00000011056	FRMD4A	protein_coding	FERM domain containing 4A
10	54270212	54270253	10	54268471	54270326	3'UTR	ENSSSCG00000032444	PLXDC2	protein_coding	plexin domain containing 2
10	56027649	56028118	10	56027733	56027816	CDS	ENSSSCG00000032896		protein_coding	
10	64497855	64498447	10	64498388	64498505	CDS	ENSSSCG00000011131	PRKCQ	protein_coding	protein kinase C theta
10	64497855	64498447	10	64498378	64498387	5'UTR	ENSSSCG00000011131	PRKCQ	protein_coding	protein kinase C theta
10	64497855	64498447	10	64498028	64498198	5'UTR	ENSSSCG00000011131	PRKCQ	protein_coding	protein kinase C theta
10	64497855	64498447	10	64498378	64498505	5'UTR	ENSSSCG00000011131	PRKCQ	protein_coding	protein kinase C theta
10	64497855	64498447	10	64498378	64498505	CDS	ENSSSCG00000011131	PRKCQ	protein_coding	protein kinase C theta
10	65705759	65705777	10	65705591	65706389	3'UTR	ENSSSCG00000039612		protein_coding	malignant T-cell-amplified sequence 1
11	1125802	1125938	11	1125630	1125807	CDS	ENSSSCG00000009276	XPO4	protein_coding	exportin 4
11	3411279	3411989	11	3411261	3411305	CDS	ENSSSCG00000035421	ATP8A2	protein_coding	phospholipid-transporting ATPase IB
11	7019135	7019798	11	7019691	7019849	CDS	ENSSSCG00000009326	KATNAL1	protein_coding	katanin catalytic subunit A1 like 1
11	7709672	7710065	11	7709696	7709837	CDS	ENSSSCG00000009333		protein_coding	
11	8818942	8819726	11	8819379	8819423	CDS	ENSSSCG00000029039	BRCA2	protein_coding	BRCA2 DNA repair associated
11	8818942	8819726	11	8819465	8820496	CDS	ENSSSCG00000029039	BRCA2	protein_coding	BRCA2 DNA repair associated
11	8818942	8819726	11	8819379	8819437	CDS	ENSSSCG00000029039	BRCA2	protein_coding	BRCA2 DNA repair associated
11	8818942	8819726	11	8819470	8820512	CDS	ENSSSCG00000029039	BRCA2	protein_coding	BRCA2 DNA repair associated
11	8818942	8819726	11	8819430	8820496	CDS	ENSSSCG00000029039	BRCA2	protein_coding	BRCA2 DNA repair associated
11	8818942	8819726	11	8819470	8820496	CDS	ENSSSCG00000029039	BRCA2	protein_coding	BRCA2 DNA repair associated
11	12232655	12233058	11	12232550	12232660	CDS	ENSSSCG00000026164		protein_coding	spermatogenesis- and oogenesis-specific basic helix-loop-helix-containing protein 2

11	12234611	12235155	11	12234641	12234749	CDS	ENSSSCG00000026164		protein_coding	spermatogenesis- and oogenesis-specific basic helix-loop-helix-containing protein 2
11	12234611	12235155	11	12234641	12234710	CDS	ENSSSCG00000026164		protein_coding	spermatogenesis- and oogenesis-specific basic helix-loop-helix-containing protein 2
11	12841890	12842404	11	12842165	12842322	CDS	ENSSSCG00000009359	SUPT20H	protein_coding	SPT20 homolog%2C SAGA complex component
11	14142130	14142355	11	14142131	14142234	CDS	ENSSSCG00000040697	PROSER1	protein_coding	proline and serine rich 1 [Source:NCBI gene;Acc:102157866]
11	14333482	14333801	11	14332604	14333674	3'UTR	ENSSSCG00000037746	NHLRC3	protein_coding	NHL repeat containing 3
11	21772352	21772864	11	21772637	21772712	CDS	ENSSSCG00000023738	COG3	protein_coding	solute carrier family 25 member 30
11	21985715	21985735	11	21983665	21987836	3'UTR	ENSSSCG00000034156	GTF2F2	protein_coding	general transcription factor IIF subunit 2
11	24704949	24705438	11	24705051	24705869	3'UTR	ENSSSCT00000040111			
11	24704949	24705438	11	24702165	24705091	3'UTR	ENSSSCT00000041854			
11	24704949	24705438	11	24705300	24705869	3'UTR	ENSSSCT00000041854			
11	24704949	24705438	11	24704880	24705869	3'UTR	ENSSSCT00000042913			
11	24704949	24705438	11	24705047	24705869	3'UTR	ENSSSCT00000043134			
11	24704949	24705438	11	24705034	24705869	3'UTR	ENSSSCT00000048214			
11	24704949	24705438	11	24705045	24705869	3'UTR	ENSSSCT00000058773			
11	24704949	24705438	11	24702159	24705869	3'UTR	ENSSSCT00000059793			
11	24704949	24705438	11	24702159	24705869	3'UTR	ENSSSCT00000064995			
11	25450865	25450895	11	25450668	25452585	3'UTR	ENSSSCG00000029837	VWA8	protein_coding	von Willebrand factor A domain containing
11	47986794	47986969	11	47986821	47986891	CDS	ENSSSCG00000040184	LMO7	protein_coding	LIM domain 7
11	68846424	68846688	11	68844378	68846470	3'UTR	ENSSSCG00000009520	ZIC2	protein_coding	Zic family member 2
11	69066614	69067165	11	69067104	69067182	CDS	ENSSSCG00000009522	PCCA	protein_coding	propionyl-CoA carboxylase subunit alpha
11	70151472	70151765	11	70151355	70151501	CDS	ENSSSCG00000009526	ITGBL1	protein_coding	integrin subunit beta like 1
12	2571692	2571985	12	2569782	2573825	3'UTR	ENSSSCG00000024261	CBX2	protein_coding	chromobox 2
12	2571692	2571985	12	2570845	2573825	3'UTR	ENSSSCG00000024261	CBX2	protein_coding	chromobox 2
12	2571692	2571985	12	2570848	2572062	3'UTR	ENSSSCG00000024261	CBX2	protein_coding	chromobox 2
12	3512506	3513087	12	3512460	3512603	CDS	ENSSSCG00000036056	DNAH17	protein_coding	dynein axonemal heavy chain 17
12	10354812	10354943	12	10351719	10355531	3'UTR	ENSSSCG00000039947	KCNJ2	protein_coding	potassium voltage-gated channel subfamily J member 2
12	10354812	10354943	12	10351715	10355531	3'UTR	ENSSSCG00000039947	KCNJ2	protein_coding	potassium voltage-gated channel subfamily J member 2
12	11234869	11235125	12	11234860	11235001	CDS	ENSSSCG00000017256	ABCA6	protein_coding	ATP binding cassette subfamily A member
12	11657612	11657830	12	11657399	11657737	3'UTR	ENSSSCG00000017258	FAM20A	protein_coding	FAM20A golgi associated secretory pathway pseudokinase
12	12495016	12495432	12	12495307	12495369	CDS	ENSSSCG00000023992	CEP112	protein_coding	centrosomal protein 112
12	15984958	15985169	12	15984974	15985093	CDS	ENSSSCG00000017299	10-Mar	protein_coding	membrane associated ring-CH-type finger
12	16130610	16130981	12	16129256	16131827	3'UTR	ENSSSCG00000017301	TLK2	protein_coding	tousled like kinase 2
12	16130610	16130981	12	16129248	16131827	3'UTR	ENSSSCG00000017301	TLK2	protein_coding	tousled like kinase 2
12	16130610	16130981	12	16130912	16131827	3'UTR	ENSSSCG00000017301	TLK2	protein_coding	tousled like kinase 2
12	16130610	16130981	12	16130909	16131827	3'UTR	ENSSSCG00000017301	TLK2	protein_coding	tousled like kinase 2
12	16881641	16881715	12	16879337	16882371	3'UTR	ENSSSCG00000017308	CDC27	protein_coding	cell division cycle 27
12	17705384	17705535	12	17705352	17708903	3'UTR	ENSSSCG00000017316	NSF	protein_coding	N-ethylmaleimide sensitive factor%2C vesicle fusing ATPase
12	17891852	17892618	12	17892399	17893290	5'UTR	ENSSSCG00000017577		protein_coding	
12	18026104	18026216	12	18025988	18026181	CDS	ENSSSCG00000017325	PLEKHM1	protein_coding	pleckstrin homology and RUN domain containing M1
12	18457442	18457884	12	18457746	18457806	CDS	ENSSSCG00000017343	GFAP	protein_coding	glial fibrillary acidic protein
12	19347130	19347162	12	19346207	19349734	3'UTR	ENSSSCG00000020744	DUSP3	protein_coding	dual specificity phosphatase 3
12	20057448	20058097	12	20057811	20057979	CDS	ENSSSCG00000040866	CNTD1	protein_coding	cyclin N-terminal domain containing 1
12	20572067	20572094	12	20571888	20573159	3'UTR	ENSSSCG00000017406	STAT5B	protein_coding	signal transducer and activator of transcription 5B
12	20572067	20572094	12	20571888	20573158	3'UTR	ENSSSCG00000017406	STAT5B	protein_coding	signal transducer and activator of transcription 5B
12	20572067	20572094	12	20571888	20574353	3'UTR	ENSSSCG00000017406	STAT5B	protein_coding	signal transducer and activator of transcription 5B
12	20572122	20572131	12	20571888	20573159	3'UTR	ENSSSCG00000017406	STAT5B	protein_coding	signal transducer and activator of transcription 5B
12	20572122	20572131	12	20571888	20573158	3'UTR	ENSSSCG00000017406	STAT5B	protein_coding	signal transducer and activator of transcription 5B
12	20572122	20572131	12	20571888	20574353	3'UTR	ENSSSCG00000017406	STAT5B	protein_coding	signal transducer and activator of transcription 5B
12	21951286	21951584	12	21950727	21951460	3'UTR	ENSSSCG00000017470	TNS4	protein_coding	tensin 4
12	21963683	21963979	12	21962639	21965581	3'UTR	ENSSSCT00000042372			
12	22099099	22099403	12	22097390	22102012	3'UTR	ENSSSCT00000054346			
12	22577678	22577812	12	22577598	22577735	CDS	ENSSSCG00000017495	GRB7	protein_coding	growth factor receptor bound protein 7
12	22774515	22775231	12	22774292	22777589	3'UTR	ENSSSCG00000017504	CDK12	protein_coding	cyclin dependent kinase 12
12	22917650	22918248	12	22917702	22917763	CDS	ENSSSCG00000017506	FBXL20	protein_coding	F-box and leucine rich repeat protein 20
12	23143281	23143313	12	23141341	23143923	3'UTR	ENSSSCG00000017511	PLXDC1	protein_coding	plexin domain containing 1
12	24315579	24315601	12	24313883	24316764	3'UTR	ENSSSCG00000017525	NFE2L1	protein_coding	nuclear factor%2C erythroid 2 like 1
12	24315579	24315601	12	24313883	24316763	3'UTR	ENSSSCG00000017525	NFE2L1	protein_coding	nuclear factor%2C erythroid 2 like 1
12	24413894	24414098	12	24413923	24414123	CDS	ENSSSCG00000017527	SKAP1	protein_coding	src kinase associated phosphoprotein 1
12	24415985	24416647	12	24416310	24416373	CDS	ENSSSCG00000017527	SKAP1	protein_coding	src kinase associated phosphoprotein 1
12	25160614	25160742	12	25159807	25160917	3'UTR	ENSSSCG00000026330	GIP	protein_coding	gastric inhibitory polypeptide
12	25508153	25508407	12	25507809	25508720	CDS	ENSSSCG00000017546	ZNF652	protein_coding	zinc finger protein 652
12	26270559	26270576	12	26267995	26271561	3'UTR	ENSSSCG00000017578	ITGA3	protein_coding	integrin subunit alpha 3
12	26270559	26270576	12	26267233	26271561	3'UTR	ENSSSCG00000017578	ITGA3	protein_coding	integrin subunit alpha 3
12	26270559	26270576	12	26267043	26271561	3'UTR	ENSSSCG00000017578	ITGA3	protein_coding	integrin subunit alpha 3

12	31643339	31643369	12	31639497	31644080	3'UTR	ENSSSCG00000017604	HLF	protein_coding	HLF transcription factor%2C PAR bZIP family member
12	31643339	31643369	12	31639497	31643593	3'UTR	ENSSSCG00000017604	HLF	protein_coding	HLF transcription factor%2C PAR bZIP family member
12	35399037	35399053	12	35396569	35399286	3'UTR	ENSSSCG00000034217	SKA2	protein_coding	spindle and kinetochore associated complex subunit 2
12	39088219	39088951	12	39088500	39088685	CDS	ENSSSCG00000021275	DDX52	protein_coding	DExD-box helicase 52
12	45693717	45694163	12	45694158	45694249	CDS	ENSSSCG00000017790	TAOK1	protein_coding	TAO kinase 1
12	49612915	49613491	12	49612820	49613080	CDS	ENSSSCG00000017861	ASPA	protein_coding	aspartoacylase
12	49612915	49613491	12	49612845	49613080	CDS	ENSSSCG00000017861	ASPA	protein_coding	aspartoacylase
12	49630667	49631035	12	49630304	49630778	3'UTR	ENSSSCG00000017861	ASPA	protein_coding	aspartoacylase
12	50540339	50540603	12	50540272	50540415	CDS	ENSSSCG00000017884	TEKT1	protein_coding	tektin 1
12	50732432	50732503	12	50731529	50734781	3'UTR	ENSSSCG00000035820	TXNDC17	protein_coding	thioredoxin domain containing 17
12	51953367	51953473	12	51953170	51953585	3'UTR	ENSSSCG00000017902	CAMTA2	protein_coding	calmodulin binding transcription activator 2
12	51953367	51953473	12	51949957	51954761	3'UTR	ENSSSCG00000017904	ENO3	protein_coding	enolase 3
12	52103099	52103147	12	52101537	52104733	3'UTR	ENSSSCG00000017914	GLTPD2	protein_coding	glycolipid transfer protein domain
12	52228369	52228560	12	52227979	52228739	CDS	ENSSSCG00000017924	PELP1	protein_coding	proline%2C glutamate and leucine rich
12	53115395	53115592	12	53114194	53117122	3'UTR	ENSSSCG00000021363	CHD3	protein_coding	chromodomain helicase DNA binding
12	54556882	54557475	12	54556923	54557022	CDS	ENSSSCG00000017994	CFAP52	protein_coding	cilia and flagella associated protein 52
13	987289	987567	13	987426	987517	CDS	ENSSSCG00000011179	MRPL3	protein_coding	mitochondrial ribosomal protein L3
13	1744547	1744757	13	1744416	1744549	CDS	ENSSSCG00000029094	PIK3R4	protein_coding	phosphoinositide-3-kinase regulatory
13	1918333	1918607	13	1918349	1918441	CDS	ENSSSCG00000037430	COL6A6	protein_coding	collagen type VI alpha 6 chain
13	2500686	2500904	13	2500637	2500767	CDS	ENSSSCG00000030697	CAPN7	protein_coding	calpain 7
13	16960244	16960577	13	16960570	16960769	CDS	ENSSSCG00000038607	GADL1	protein_coding	glutamate decarboxylase like 1
13	20949051	20949768	13	20949471	20949553	5'UTR	ENSSSCT00000045527			
13	23840195	23840247	13	23840211	23840468	5'UTR	ENSSSCT00000042469			
13	24496901	24497161	13	24497156	24497236	CDS	ENSSSCG00000011269	MYRIP	protein_coding	myosin VIIA and Rab interacting protein
13	27916588	27916787	13	27916095	27917788	3'UTR	ENSSSCG00000032080	ZNF35	protein_coding	zinc finger protein 35
13	29413301	29413696	13	29411457	29414078	3'UTR	ENSSSCG00000029879	LTF	protein_coding	lactotransferrin
13	30934703	30934741	13	30934510	30934794	3'UTR	ENSSSCG00000011349	NPG3	protein_coding	peptide antibiotic PR39
13	30977921	30977959	13	30977602	30977957	3'UTR	ENSSSCG00000011349	NPG3	protein_coding	peptide antibiotic PR39
13	30987950	30987988	13	30987631	30987976	3'UTR	ENSSSCG00000011349	NPG3	protein_coding	peptide antibiotic PR39
13	33565161	33565842	13	33565686	33565786	CDS	ENSSSCG00000011416	DOCK3	protein_coding	dedicator of cytokinesis 3
13	33820194	33820218	13	33820125	33820266	CDS	ENSSSCG00000011425	RAD54L2	protein_coding	RAD54 like 2
13	34360914	34361137	13	34357850	34361738	3'UTR	ENSSSCG00000011435	TWF2	protein_coding	toll like receptor 9
13	38629581	38629911	13	38629166	38629816	5'UTR	ENSSSCG00000030359	ARHGEF3	protein_coding	Rho guanine nucleotide exchange factor 3
13	40995632	40996384	13	40995632	40995784	CDS	ENSSSCT00000056788			
13	45142090	45143105	13	45142532	45142600	CDS	ENSSSCG00000027617	SYNPR	protein_coding	synaptoporin
13	45478452	45479085	13	45478955	45479037	CDS	ENSSSCG00000011493		protein_coding	ataxin 7
13	46035104	46035180	13	46033759	46035297	3'UTR	ENSSSCG00000011496	ADAMTS9	protein_coding	ADAM metallopeptidase with thrombospondin type 1 motif 9
13	46035104	46035180	13	46034833	46035110	3'UTR	ENSSSCG00000011496	ADAMTS9	protein_coding	ADAM metallopeptidase with thrombospondin type 1 motif 9
13	46035104	46035180	13	46035150	46035297	3'UTR	ENSSSCG00000011496	ADAMTS9	protein_coding	ADAM metallopeptidase with thrombospondin type 1 motif 9
13	46035104	46035180	13	46033758	46035297	3'UTR	ENSSSCG00000011496	ADAMTS9	protein_coding	ADAM metallopeptidase with thrombospondin type 1 motif 9
13	50540786	50541539	13	50541414	50541774	3'UTR	ENSSSCT00000012594			
13	50540786	50541539	13	50540645	50541774	3'UTR	ENSSSCT00000063514			
13	52502977	52503433	13	52503349	52503450	CDS	ENSSSCG00000027675	FOXP1	protein_coding	forkhead box P1
13	67933955	67934659	13	67934546	67934608	CDS	ENSSSCG00000011578	TAMM41	protein_coding	TAM41 mitochondrial translocator assembly and maintenance homolog
13	68598753	68599289	13	68598980	68599039	CDS	ENSSSCG00000024453	RAF1	protein_coding	Raf-1 proto-oncogene%2C serine/threonine kinase
13	72345993	72346333	13	72346030	72346180	CDS	ENSSSCG00000011622	KBTBD12	protein_coding	kelch repeat and BTB domain containing 12
13	72587752	72587776	13	72587194	72587942	CDS	ENSSSCG00000021397	PODXL2	protein_coding	podocalyxin like 2
13	75075004	75075098	13	75074886	75075286	5'UTR	ENSSSCG00000036503	RAB6B	protein_coding	RAB6B%2C member RAS oncogene family
13	77416649	77417254	13	77417167	77417284	CDS	ENSSSCG00000011652	STAG1	protein_coding	stromal antigen 1
13	79069755	79070122	13	79069836	79069847	CDS	ENSSSCT00000041330			
13	79083527	79084025	13	79083150	79083809	3'UTR	ENSSSCG00000036610	ARMC8	protein_coding	armadillo repeat containing 8
13	79083527	79084025	13	79083150	79083797	3'UTR	ENSSSCG00000036610	ARMC8	protein_coding	armadillo repeat containing 8
13	82326276	82326444	13	82326208	82326291	CDS	ENSSSCG00000011672	RASA2	protein_coding	RAS p21 protein activator 2
13	83340232	83340911	13	83340048	83340694	5'UTR	ENSSSCG00000011680	PLS1	protein_coding	plastin 1
13	83340232	83340911	13	83340517	83340694	5'UTR	ENSSSCG00000011680	PLS1	protein_coding	plastin 1
13	89421371	89421651	13	89421463	89421602	CDS	ENSSSCG00000011700	CP	protein_coding	ceruloplasmin
13	89731790	89732166	13	89729840	89732092	3'UTR	ENSSSCG00000011703	TM4SF4	protein_coding	transmembrane 4 L six family member 4
13	89731790	89732166	13	89732128	89732667	3'UTR	ENSSSCG00000011703	TM4SF4	protein_coding	transmembrane 4 L six family member 4
13	91472186	91472447	13	91470932	91474355	3'UTR	ENSSSCG00000032365	GPR87	protein_coding	G protein-coupled receptor 87
13	95421605	95422185	13	95421955	95422079	CDS	ENSSSCG00000026718	PLCH1	protein_coding	phospholipase C eta 1
13	104294923	104295260	13	104294145	104296717	3'UTR	ENSSSCT00000039663			
13	104294923	104295260	13	104294145	104296717	3'UTR	ENSSSCT00000055033			
13	104294923	104295260	13	104294145	104296717	3'UTR	ENSSSCT00000056713			
13	104451534	104451581	13	104449985	104452253	3'UTR	ENSSSCG00000011736	SLITRK3	protein_coding	SLIT and NTRK like family member 3
13	106736288	106737072	13	106736243	106736444	CDS	ENSSSCG00000040950		protein_coding	
13	106736288	106737072	13	106736445	106738337	3'UTR	ENSSSCG00000040950		protein_coding	
13	106736288	106737072	13	106736243	106736440	CDS	ENSSSCG00000040950		protein_coding	
13	108518240	108518256	13	108517310	108522018	3'UTR	ENSSSCG00000032225	LRRC31	protein_coding	leucine rich repeat containing 31
13	111022378	111022649	13	111021958	111024467	3'UTR	ENSSSCG00000020906	TNFSF10	protein_coding	TNF superfamily member 10
13	111022378	111022649	13	111021975	111024467	3'UTR	ENSSSCG00000020906	TNFSF10	protein_coding	TNF superfamily member 10
13	111022378	111022649	13	111022029	111024517	3'UTR	ENSSSCG00000020906	TNFSF10	protein_coding	TNF superfamily member 10

13	117928278	117929042	13	117928802	117928898	CDS	ENSSSCG00000011766	PEX5L	protein_coding	peroxisomal biogenesis factor 5 like
13	122488157	122488191	13	122487521	122488295	3'UTR	ENSSSCG00000026516	EPHB3	protein_coding	EPH receptor B3
13	124513957	124514479	13	124514018	124514119	CDS	ENSSSCG00000011801	HRG	protein_coding	histidine rich glycoprotein
13	127328923	127329002	13	127328895	127330224	3'UTR	ENSSSCG00000025592	TP63	protein_coding	tumor protein p63
13	128072369	128072843	13	128071398	128075570	3'UTR	ENSSSCG00000021206	IL1RAP	protein_coding	interleukin 1 receptor accessory protein
13	132376212	132376710	13	132376272	132376343	CDS	ENSSSCG00000031147	ACAP2	protein_coding	ArfGAP with coiled-coil%2C ankyrin repeat and PH domains 2
13	132376212	132376710	13	132376344	132376644	5'UTR	ENSSSCG00000031147	ACAP2	protein_coding	ArfGAP with coiled-coil%2C ankyrin repeat and PH domains 2
13	137688116	137688558	13	137688342	137688471	CDS	ENSSSCG00000036766	SLC49A4	protein_coding	solute carrier family 49 member 4
13	138729351	138730018	13	138729842	138729990	CDS	ENSSSCG00000011881	IQCB1	protein_coding	IQ motif containing B1
13	139127317	139127781	13	139127460	139127571	CDS	ENSSSCG00000039888	STXBP5L	protein_coding	syntaxin binding protein 5 like
13	139135311	139135744	13	139135692	139136052	CDS	ENSSSCG00000039888	STXBP5L	protein_coding	syntaxin binding protein 5 like
13	140838904	140839228	13	140838661	140841593	3'UTR	ENSSSCG00000026224	ARHGAP31	protein_coding	Rho GTPase activating protein 31
13	146130436	146131241	13	146130755	146130926	CDS	ENSSSCG00000011916	ATP6V1A	protein_coding	ATPase H+ transporting V1 subunit A
13	146130436	146131241	13	146130755	146130766	CDS	ENSSSCG00000011916	ATP6V1A	protein_coding	ATPase H+ transporting V1 subunit A
13	146130436	146131241	13	146130805	146130926	CDS	ENSSSCG00000011916	ATP6V1A	protein_coding	ATPase H+ transporting V1 subunit A
13	148534691	148535551	13	148534605	148534946	CDS	ENSSSCG00000011933	NECTIN3	protein_coding	nectin cell adhesion molecule 3
13	159216706	159216727	13	159216142	159218902	3'UTR	ENSSSCG00000011973	COL8A1	protein_coding	collagen type VIII alpha 1 chain
13	159216706	159216727	13	159216145	159218902	3'UTR	ENSSSCG00000011973	COL8A1	protein_coding	collagen type VIII alpha 1 chain
13	173876232	173877001	13	173876905	173877035	CDS	ENSSSCG00000012000	GBE1	protein_coding	1%2C4-alpha-glucan branching enzyme 1
13	192942977	192943367	13	192942975	192943019	CDS	ENSSSCG00000025119	GRIK1	protein_coding	glutamate ionotropic receptor kainate type subunit 1
13	198290792	198290965	13	198290778	198290887	CDS	ENSSSCG00000028112	CLIC6	protein_coding	chloride intracellular channel 6
13	198290792	198290965	13	198290798	198290887	CDS	ENSSSCG00000028112	CLIC6	protein_coding	chloride intracellular channel 6
13	200408351	200408381	13	200405063	200409405	3'UTR	ENSSSCG00000012059	HLCS	protein_coding	holocarboxylase synthetase
13	200408351	200408381	13	200407559	200409405	3'UTR	ENSSSCG00000012059	HLCS	protein_coding	holocarboxylase synthetase
13	200408351	200408381	13	200405064	200409405	3'UTR	ENSSSCG00000012059	HLCS	protein_coding	holocarboxylase synthetase
13	204830701	204830747	13	204829377	204831975	3'UTR	ENSSSCG00000012076	MX2	protein_coding	Interferon-induced GTP-binding protein
13	205568565	205569181	13	205569039	205569282	CDS	ENSSSCG00000027745	ABCG1	protein_coding	ATP binding cassette subfamily G member
13	206192793	206192893	13	206192624	206193086	3'UTR	ENSSSCG00000037663	PKNOX1	protein_coding	PBX/knotted 1 homeobox 1
13	206192793	206192893	13	206192624	206195468	3'UTR	ENSSSCG00000037663	PKNOX1	protein_coding	PBX/knotted 1 homeobox 1
13	206192793	206192893	13	206192624	206193231	3'UTR	ENSSSCG00000037663	PKNOX1	protein_coding	PBX/knotted 1 homeobox 1
14	6253471	6253575	14	6253357	6253476	CDS	ENSSSCG00000009611	XPO7	protein_coding	exportin 7
14	6797003	6797086	14	6793368	6797676	3'UTR	ENSSSCG00000032622	PPP3CC	protein_coding	solute carrier family 39 member 14
14	8432596	8433025	14	8431181	8433350	3'UTR	ENSSSCG00000009644	ADAM28	protein_coding	ADAM metallopeptidase domain 28
14	11197723	11197757	14	11197532	11197750	CDS	ENSSSCG00000009665	CHRNA2	protein_coding	cholinergic receptor nicotinic alpha 2
14	11197723	11197757	14	11197532	11197753	CDS	ENSSSCG00000009665	CHRNA2	protein_coding	cholinergic receptor nicotinic alpha 2
14	13967692	13967708	14	13967576	13967952	3'UTR	ENSSSCG00000009692	PINX1	protein_coding	PIN2 (TERF1) interacting telomerase
14	13967692	13967708	14	13967585	13967952	3'UTR	ENSSSCG00000009692	PINX1	protein_coding	PIN2 (TERF1) interacting telomerase
14	14085760	14086003	14	14084981	14087418	3'UTR	ENSSSCG00000009693	XKR6	protein_coding	XK related 6
14	15172166	15172379	14	15170853	15174169	3'UTR	ENSSSCG00000032245	DEFB134	protein_coding	defensin beta 134
14	16657715	16658039	14	16657496	16657737	CDS	ENSSSCG00000032048	SCRG1	protein_coding	stimulator of chondrogenesis 1
14	20040040	20040055	14	20039721	20040066	3'UTR	ENSSSCG00000009713	CLCN3	protein_coding	chloride voltage-gated channel 3
14	20040040	20040055	14	20039999	20040157	CDS	ENSSSCG00000009713	CLCN3	protein_coding	chloride voltage-gated channel 3
14	20040040	20040055	14	20037634	20040066	3'UTR	ENSSSCG00000009713	CLCN3	protein_coding	chloride voltage-gated channel 3
14	20307771	20308022	14	20307744	20307832	CDS	ENSSSCG00000009714	NEK1	protein_coding	NIMA related kinase 1
14	22846670	22846959	14	22846886	22847069	CDS	ENSSSCG00000033051	POLE	protein_coding	DNA polymerase epsilon%2C catalytic
14	30277319	30278036	14	30278012	30278115	CDS	ENSSSCG00000009792	ZCCHC8	protein_coding	zinc finger CCHC-type containing 8
14	30874001	30874022	14	30873786	30874232	CDS	ENSSSCT00000010749			
14	31210749	31211153	14	31210706	31210831	CDS	ENSSSCG00000037811	RNF34	protein_coding	ring finger protein 34
14	31416304	31416678	14	31415191	31418629	3'UTR	ENSSSCG00000009817	P2RX7	protein_coding	purinergic receptor P2X 7
14	31741899	31742055	14	31740935	31742258	3'UTR	ENSSSCG00000034386	ATP2A2	protein_coding	ATPase sarcoplasmic/endoplasmic reticulum Ca2+ transporting 2
14	31741899	31742055	14	31740935	31744978	3'UTR	ENSSSCG00000034386	ATP2A2	protein_coding	ATPase sarcoplasmic/endoplasmic reticulum Ca2+ transporting 2
14	40571996	40572662	14	40572585	40572629	CDS	ENSSSCT00000062818			
14	40611837	40612143	14	40611687	40611916	CDS	ENSSSCG00000009914	MLEC	protein_coding	malectin
14	40611837	40612143	14	40611917	40612036	3'UTR	ENSSSCG00000009914	MLEC	protein_coding	malectin
14	40611837	40612143	14	40612103	40617296	3'UTR	ENSSSCG00000009914	MLEC	protein_coding	malectin
14	40611837	40612143	14	40611917	40617381	3'UTR	ENSSSCG00000009914	MLEC	protein_coding	malectin
14	40612496	40612520	14	40612103	40617296	3'UTR	ENSSSCG00000009914	MLEC	protein_coding	malectin
14	40612496	40612520	14	40611917	40617381	3'UTR	ENSSSCG00000009914	MLEC	protein_coding	malectin
14	40888235	40888427	14	40884585	40889202	3'UTR	ENSSSCT00000028422			
14	40888235	40888427	14	40887199	40890469	3'UTR	ENSSSCG00000009919	HNF1A	protein_coding	HNF1 homeobox A
14	42144582	42144673	14	42142812	42145124	3'UTR	ENSSSCG00000009944	CORO1C	protein_coding	coronin 1C
14	42488785	42488952	14	42487420	42490855	3'UTR	ENSSSCG00000039261	WSCD2	protein_coding	WSC domain containing 2
14	42852808	42853400	14	42852843	42852960	CDS	ENSSSCG00000009950		protein_coding	piwi like RNA-mediated gene silencing 3
14	49005491	49005533	14	49004507	49005603	3'UTR	ENSSSCG00000038719		protein_coding	
14	49008612	49008645	14	49007623	49008640	3'UTR	ENSSSCG00000038719		protein_coding	
14	50603279	50603583	14	50603323	50603460	CDS	ENSSSCG00000010100	LRRC74B	protein_coding	leucine rich repeat containing 74B
14	54849648	54850012	14	54849917	54850020	CDS	ENSSSCG00000010145	HEATR1	protein_coding	HEAT repeat containing 1
14	55880849	55881263	14	55880862	55880961	CDS	ENSSSCG00000034844		protein_coding	tubulin folding cofactor E
14	55880849	55881263	14	55880962	55880995	5'UTR	ENSSSCG00000034844		protein_coding	tubulin folding cofactor E
14	56792171	56792840	14	56792319	56792408	CDS	ENSSSCG00000010162	SLC35F3	protein_coding	solute carrier family 35 member F3
14	59204773	59205045	14	59204752	59204813	CDS	ENSSSCG00000010175	GNPAT	protein_coding	glyceronephosphate O-acyltransferase
14	62645941	62646229	14	62645781	62645991	CDS	ENSSSCG00000010209	FAM13C	protein_coding	family with sequence similarity 13 member
14	63176155	63176227	14	63175653	63179765	3'UTR	ENSSSCG00000010211	CCDC6	protein_coding	coiled-coil domain containing 6
14	73132199	73132848	14	73132682	73132713	CDS	ENSSSCG00000010261	PPA1	protein_coding	inorganic pyrophosphatase 1 [Source:NCBI gene;Acc:100155201]

14	75229090	75229944	14	75229449	75232002	3'UTR	ENSSSCT00000040682			
14	75678560	75679268	14	75678941	75679010	CDS	ENSSSCG00000010290	MCU	protein_coding	mitochondrial calcium uniporter
14	75700925	75701254	14	75700988	75701158	CDS	ENSSSCG00000010290	MCU	protein_coding	mitochondrial calcium uniporter
14	75974417	75975063	14	75974900	75975028	CDS	ENSSSCG00000010295	ECD	protein_coding	ecdysoneless cell cycle regulator
14	76077138	76077451	14	76075423	76078269	3'UTR	ENSSSCT00000038043			
14	76077138	76077451	14	76077214	76077261	CDS	ENSSSCG00000010298	CFAP70	protein_coding	cilia and flagella associated protein 70
14	76456898	76457195	14	76456173	76457686	3'UTR	ENSSSCG00000010303	SYNPO2L	protein_coding	synaptopodin 2 like
14	76456898	76457195	14	76456168	76457686	3'UTR	ENSSSCG00000010303	SYNPO2L	protein_coding	synaptopodin 2 like
14	77586750	77587765	14	77586686	77587027	CDS	ENSSSCG00000010316	KAT6B	protein_coding	lysine acetyltransferase 6B
14	88520086	88520470	14	88517068	88521604	3'UTR	ENSSSCG00000010376	GDF10	protein_coding	growth differentiation factor 10
14	90168017	90168104	14	90167540	90170827	3'UTR	ENSSSCG00000010392	CHAT	protein_coding	choline O-acetyltransferase
14	90683492	90684212	14	90683604	90683790	CDS	ENSSSCG00000010402	ZFAND4	protein_coding	zinc finger AN1-type containing 4
14	96371650	96372330	14	96371050	96372532	CDS	ENSSSCG00000010426	PCDH15	protein_coding	protocadherin related 15
14	107349229	107349394	14	107349218	107349345	CDS	ENSSSCG00000010497	ENTPD1	protein_coding	ectonucleoside triphosphate diphosphohydrolase 1
14	107349229	107349394	14	107349217	107349345	CDS	ENSSSCG00000010497	ENTPD1	protein_coding	ectonucleoside triphosphate diphosphohydrolase 1
14	109589158	109589338	14	109588919	109589192	5'UTR	ENSSSCG00000010532	LOXL4	protein_coding	lysyl oxidase like 4
14	109764258	109764836	14	109764514	109765170	3'UTR	ENSSSCG00000010535	HPSE2	protein_coding	heparanase 2 (inactive)
14	112095631	112095974	14	112095864	112095997	CDS	ENSSSCG00000010559		protein_coding	PDZ domain containing 7
14	112095631	112095974	14	112095823	112095863	3'UTR	ENSSSCG00000010559		protein_coding	PDZ domain containing 7
14	123968741	123968987	14	123968789	123968887	CDS	ENSSSCG00000010640	NRAP	protein_coding	nebulin related anchoring protein
14	124762651	124762787	14	124761462	124764733	3'UTR	ENSSSCG00000010651	ABLIM1	protein_coding	actin binding LIM protein 1
14	124762651	124762787	14	124760404	124764733	3'UTR	ENSSSCG00000010651	ABLIM1	protein_coding	actin binding LIM protein 1
14	124762651	124762787	14	124760808	124764733	3'UTR	ENSSSCG00000010651	ABLIM1	protein_coding	actin binding LIM protein 1
14	124762651	124762787	14	124761463	124764733	3'UTR	ENSSSCG00000010651	ABLIM1	protein_coding	actin binding LIM protein 1
14	124763367	124763595	14	124761462	124764733	3'UTR	ENSSSCG00000010651	ABLIM1	protein_coding	actin binding LIM protein 1
14	124763367	124763595	14	124760404	124764733	3'UTR	ENSSSCG00000010651	ABLIM1	protein_coding	actin binding LIM protein 1
14	124763367	124763595	14	124760808	124764733	3'UTR	ENSSSCG00000010651	ABLIM1	protein_coding	actin binding LIM protein 1
14	124763367	124763595	14	124761463	124764733	3'UTR	ENSSSCG00000010651	ABLIM1	protein_coding	actin binding LIM protein 1
14	128967113	128967402	14	128966495	128968637	3'UTR	ENSSSCG00000010679	EIF3A	protein_coding	eukaryotic translation initiation factor 3 subunit A
14	128967113	128967402	14	128966336	128968637	3'UTR	ENSSSCG00000010679	EIF3A	protein_coding	eukaryotic translation initiation factor 3 subunit A
14	128970517	128970882	14	128970365	128970540	CDS	ENSSSCG00000010679	EIF3A	protein_coding	eukaryotic translation initiation factor 3 subunit A
14	132308818	132309422	14	132308913	132308931	CDS	ENSSSCG00000002978	PSP	protein_coding	porcine seminal protein I
14	132308818	132309422	14	132309107	132309111	CDS	ENSSSCG00000002978	PSP	protein_coding	porcine seminal protein I
14	132308818	132309422	14	132309112	132309435	3'UTR	ENSSSCG00000002978	PSP	protein_coding	porcine seminal protein I
14	141249031	141249507	14	141248199	141249909	5'UTR	ENSSSCT00000040893			
15	3192363	3192700	15	3190343	3194131	3'UTR	ENSSSCG00000022919	KIF5C	protein_coding	kinesin family member 5C
15	10848494	10848588	15	10848288	10848533	CDS	ENSSSCG00000023746	LRP1B	protein_coding	
15	13135112	13135871	15	13134226	13138261	3'UTR	ENSSSCG00000032198	SPOPL	protein_coding	speckle type BTB/POZ protein like
15	13135112	13135871	15	13134225	13138261	3'UTR	ENSSSCG00000032198	SPOPL	protein_coding	speckle type BTB/POZ protein like
15	21956424	21957446	15	21956524	21956615	CDS	ENSSSCG00000015711	DPP10	protein_coding	dipeptidyl peptidase like 10
15	21956424	21957446	15	21957078	21957125	CDS	ENSSSCG00000015711	DPP10	protein_coding	dipeptidyl peptidase like 10
15	23697658	23698300	15	23697538	23698601	3'UTR	ENSSSCG00000036229	INSIG2	protein_coding	insulin induced gene 2
15	23697658	23698300	15	23697538	23699590	3'UTR	ENSSSCG00000036229	INSIG2	protein_coding	insulin induced gene 2
15	27046274	27046586	15	27046355	27046369	CDS	ENSSSCT00000056606			
15	29986007	29986678	15	29986097	29986260	CDS	ENSSSCG00000039157	CLASP1	protein_coding	cytoplasmic linker associated protein 1
15	33590838	33591540	15	33591537	33591650	3'UTR	ENSSSCG00000015747		protein_coding	myomesin 2
15	37852625	37853185	15	37851130	37853437	3'UTR	ENSSSCG00000015755	AGPAT5	protein_coding	1-acylglycerol-3-phosphate O-
15	37936900	37936956	15	37936423	37939954	3'UTR	ENSSSCG00000036877		protein_coding	zinc finger protein 705A-like
15	37936900	37936956	15	37935973	37939954	3'UTR	ENSSSCG00000036877		protein_coding	zinc finger protein 705A-like
15	38210608	38210879	15	38210368	38211792	3'UTR	ENSSSCG00000035210		protein_coding	
15	47216885	47217485	15	47216985	47217061	CDS	ENSSSCG00000045907	ADAM2	protein_coding	ADAM metallopeptidase domain 2 [Source:NCBI gene;Acc:397006]
15	47226772	47227154	15	47227028	47227084	CDS	ENSSSCG00000045907	ADAM2	protein_coding	ADAM metallopeptidase domain 2 [Source:NCBI gene;Acc:397006]
15	48240359	48240520	15	48240015	48241776	3'UTR	ENSSSCG00000015820	NSD3	protein_coding	nuclear receptor binding SET domain
15	48240359	48240520	15	48239773	48241776	3'UTR	ENSSSCG00000015820	NSD3	protein_coding	nuclear receptor binding SET domain
15	48240359	48240520	15	48240015	48241800	3'UTR	ENSSSCG00000015820	NSD3	protein_coding	nuclear receptor binding SET domain
15	48240359	48240520	15	48239773	48241774	3'UTR	ENSSSCG00000015820	NSD3	protein_coding	nuclear receptor binding SET domain
15	68929682	68930062	15	68928254	68931312	3'UTR	ENSSSCG00000015897	IFIH1	protein_coding	interferon induced with helicase C domain 1
15	70262577	70263130	15	70262091	70264033	3'UTR	ENSSSCG00000034262	FIGN	protein_coding	fidgetin%2C microtubule severing factor
15	71434365	71435001	15	71434906	71435021	CDS	ENSSSCG00000025598	COBLL1	protein_coding	cordon-bleu WH2 repeat protein like 1
15	71434365	71435001	15	71434906	71434983	5'UTR	ENSSSCG00000025598	COBLL1	protein_coding	cordon-bleu WH2 repeat protein like 1
15	72748596	72749134	15	72748988	72750180	CDS	ENSSSCG00000015913	SCN9A	protein_coding	sodium voltage-gated channel alpha subunit
15	72748596	72749134	15	72745114	72748987	3'UTR	ENSSSCG00000015913	SCN9A	protein_coding	sodium voltage-gated channel alpha subunit
15	75310311	75310732	15	75310602	75310685	CDS	ENSSSCG00000015923	NOSTRIN	protein_coding	nitric oxide synthase trafficking
15	77689866	77690403	15	77690106	77690260	CDS	ENSSSCG00000025049	CYBRD1	protein_coding	cytochrome b reductase 1
15	80412064	80412647	15	80412301	80412471	CDS	ENSSSCG00000023297	SCRN3	protein_coding	secernin 3
15	80412064	80412647	15	80412472	80414829	3'UTR	ENSSSCG00000023297	SCRN3	protein_coding	secernin 3
15	80412064	80412647	15	80412472	80417243	3'UTR	ENSSSCG00000023297	SCRN3	protein_coding	secernin 3
15	85547755	85547842	15	85547718	85547944	CDS	ENSSSCG00000016008	CWC22	protein_coding	CWC22 spliceosome associated protein
15	88332617	88333581	15	88332857	88334030	3'UTR	ENSSSCG00000016018	FRZB	protein_coding	frizzled related protein
15	88332617	88333581	15	88332860	88334030	3'UTR	ENSSSCG00000016018	FRZB	protein_coding	frizzled related protein
15	99860968	99861809	15	99861200	99861298	CDS	ENSSSCG00000032814	DNAH7	protein_coding	dynein axonemal heavy chain 7
15	101593907	101594619	15	101594215	101594306	CDS	ENSSSCG00000016082	BOLL	protein_coding	boule homolog%2C RNA binding protein
15	104556172	104556387	15	104556147	104556241	CDS	ENSSSCG00000016095	CLK1	protein_coding	CDC like kinase 1

15	104556172	104556387	15	104556147	104556241	3'UTR	ENSSSCG00000016095	CLK1	protein_coding	CDC like kinase 1
15	104892566	104892596	15	104889425	104894921	3'UTR	ENSSSCG00000026940	CASP10	protein_coding	caspase 10
15	109070846	109071350	15	109071336	109071616	CDS	ENSSSCG00000026383	NRP2	protein_coding	neuropilin 2
15	109090171	109090183	15	109089289	109092308	3'UTR	ENSSSCG00000026383	NRP2	protein_coding	neuropilin 2
15	109090171	109090183	15	109089289	109093525	3'UTR	ENSSSCG00000026383	NRP2	protein_coding	neuropilin 2
15	109092965	109093689	15	109089289	109093525	3'UTR	ENSSSCG00000026383	NRP2	protein_coding	neuropilin 2
15	115525697	115526379	15	115526111	115526254	CDS	ENSSSCG00000016164	IKZF2	protein_coding	IKAROS family zinc finger 2
15	117728254	117728740	15	117728475	117728612	CDS	ENSSSCG00000016174	FN1	protein_coding	fibronectin 1
15	118350738	118351003	15	118350146	118350768	CDS	ENSSSCG00000016177	XRCC5	protein_coding	X-ray repair cross complementing 5 [Source:NCBI gene;Acc:100514133]
15	118350738	118351003	15	118350769	118352702	3'UTR	ENSSSCG00000016177	XRCC5	protein_coding	X-ray repair cross complementing 5 [Source:NCBI gene;Acc:100514133]
15	118629002	118629294	15	118629147	118629148	CDS	ENSSSCG00000035501	SMARCAL1	protein_coding	SWI/SNF related%2C matrix associated%2C actin dependent regulator of chromatin%2C subfamily a like 1
15	118629002	118629294	15	118629223	118629260	CDS	ENSSSCG00000035501	SMARCAL1	protein_coding	SWI/SNF related%2C matrix associated%2C actin dependent regulator of chromatin%2C subfamily a like 1
15	120496615	120497178	15	120493374	120497238	3'UTR	ENSSSCG00000016194	USP37	protein_coding	ubiquitin specific peptidase 37
15	120496615	120497178	15	120497151	120497238	CDS	ENSSSCG00000016194	USP37	protein_coding	ubiquitin specific peptidase 37
15	120496615	120497178	15	120492554	120497150	3'UTR	ENSSSCG00000016194	USP37	protein_coding	ubiquitin specific peptidase 37
15	120496615	120497178	15	120492554	120497770	3'UTR	ENSSSCG00000016194	USP37	protein_coding	ubiquitin specific peptidase 37
15	120496615	120497178	15	120492555	120497150	3'UTR	ENSSSCG00000016194	USP37	protein_coding	ubiquitin specific peptidase 37
15	120548827	120549381	15	120549038	120549196	CDS	ENSSSCG00000016194	USP37	protein_coding	ubiquitin specific peptidase 37
15	121271274	121271440	15	121271107	121271336	CDS	ENSSSCG00000016212	ANKZF1	protein_coding	ankyrin repeat and zinc finger domain containing 1
15	121271274	121271440	15	121271337	121272736	3'UTR	ENSSSCG00000016212	ANKZF1	protein_coding	ankyrin repeat and zinc finger domain containing 1
15	121327078	121327114	15	121326475	121327381	3'UTR	ENSSSCG00000016217	DNAJB2	protein_coding	DnaJ heat shock protein family (Hsp40) member B2
15	121327078	121327114	15	121325527	121327386	3'UTR	ENSSSCG00000016217	DNAJB2	protein_coding	DnaJ heat shock protein family (Hsp40) member B2
15	121327078	121327114	15	121326475	121327389	3'UTR	ENSSSCG00000016217	DNAJB2	protein_coding	DnaJ heat shock protein family (Hsp40) member B2
15	121327078	121327114	15	121326475	121327382	3'UTR	ENSSSCG00000016217	DNAJB2	protein_coding	DnaJ heat shock protein family (Hsp40) member B2
15	121327078	121327114	15	121325527	121327384	3'UTR	ENSSSCG00000016217	DNAJB2	protein_coding	DnaJ heat shock protein family (Hsp40) member B2
15	124094675	124095374	15	124095296	124095330	CDS	ENSSSCG00000028418	PAX3	protein_coding	paired box 3
15	124190641	124191280	15	124191044	124191279	CDS	ENSSSCG00000028418	PAX3	protein_coding	paired box 3
15	124918171	124918487	15	124913767	124918536	3'UTR	ENSSSCG00000038149	KCNE4	protein_coding	potassium voltage-gated channel subfamily E regulatory subunit 4
15	126516204	126516874	15	126516837	126516929	CDS	ENSSSCG00000016237	DOCK10	protein_coding	dedicator of cytokinesis 10
15	131659289	131659646	15	131658414	131659384	CDS	ENSSSCG00000035592	GPR55	protein_coding	G protein-coupled receptor 55
15	131794921	131794971	15	131794398	131795555	3'UTR	ENSSSCG00000016270	C2orf72	protein_coding	chromosome 15 C2orf72 homolog
15	133784853	133785213	15	133785041	133785067	CDS	ENSSSCG00000009432	DGKD	protein_coding	diacylglycerol kinase delta
15	134044565	134045011	15	134044794	134044925	CDS	ENSSSCG00000036274		protein_coding	UDP glucuronosyltransferase 1 family%2C polypeptide A6
15	134047656	134047699	15	134046125	134050844	3'UTR	ENSSSCG00000036274		protein_coding	UDP glucuronosyltransferase 1 family%2C polypeptide A6
15	139647530	139647563	15	139647440	139647700	5'UTR	ENSSSCG00000016401	KIF1A	protein_coding	kinesin family member 1A
15	139647530	139647563	15	139646918	139647700	CDS	ENSSSCG00000016401	KIF1A	protein_coding	kinesin family member 1A [Source:NCBI gene;Acc:100517246]
15	140331264	140331700	15	140327703	140331274	3'UTR	ENSSSCG00000037090	NEU4	protein_coding	neuraminidase 4
16	4095126	4095172	16	4091879	4097090	3'UTR	ENSSSCG00000034714	OTULIN	protein_coding	OTU deubiquitinase with linear linkage specificity
16	4095711	4095754	16	4091879	4097090	3'UTR	ENSSSCG00000034714	OTULIN	protein_coding	OTU deubiquitinase with linear linkage specificity
16	22599731	22600100	16	22599973	22600047	5'UTR	ENSSSCG00000016846	WDR70	protein_coding	WD repeat domain 70
16	22599731	22600100	16	22600002	22600047	CDS	ENSSSCG00000016846	WDR70	protein_coding	WD repeat domain 70
16	22599731	22600100	16	22599576	22600001	5'UTR	ENSSSCG00000016846	WDR70	protein_coding	WD repeat domain 70
16	30305875	30305999	16	30305684	30306176	5'UTR	ENSSSCG00000016882	PARP8	protein_coding	poly(ADP-ribose) polymerase family
16	30305875	30305999	16	30305961	30306078	CDS	ENSSSCG00000016882	PARP8	protein_coding	poly(ADP-ribose) polymerase family
16	30305875	30305999	16	30305663	30305960	5'UTR	ENSSSCG00000016882	PARP8	protein_coding	poly(ADP-ribose) polymerase family
16	30972531	30972856	16	30970981	30973907	3'UTR	ENSSSCG00000016883	ISL1	protein_coding	ISL LIM homeobox 1
16	32416463	32416968	16	32416502	32416594	CDS	ENSSSCG00000016887	ITGA2	protein_coding	integrin subunit alpha 2
16	34227011	34227475	16	34226093	34227565	3'UTR	ENSSSCG00000016901	GZMK	protein_coding	granzyme K
16	35999658	35999854	16	35999623	35999717	CDS	ENSSSCG00000016920	MIER3	protein_coding	MIER family member 3
16	43173706	43174370	16	43174110	43174257	CDS	ENSSSCG00000033581	RGS7BP	protein_coding	regulator of G protein signaling 7 binding
16	52336166	52336704	16	52335407	52337888	3'UTR	ENSSSCG00000023307	FBXW11	protein_coding	F-box and WD repeat domain containing 11
16	52336166	52336704	16	52335407	52337913	3'UTR	ENSSSCG00000023307	FBXW11	protein_coding	F-box and WD repeat domain containing 11
16	52336166	52336704	16	52335407	52337890	3'UTR	ENSSSCG00000023307	FBXW11	protein_coding	F-box and WD repeat domain containing 11
16	52336166	52336704	16	52335407	52337110	3'UTR	ENSSSCG00000023307	FBXW11	protein_coding	F-box and WD repeat domain containing 11
16	52336166	52336704	16	52335407	52337730	3'UTR	ENSSSCG00000023307	FBXW11	protein_coding	F-box and WD repeat domain containing 11
16	53204891	53205141	16	53203887	53207271	3'UTR	ENSSSCG00000021902	GABRP	protein_coding	gamma-aminobutyric acid type A receptor pi subunit
16	53204891	53205141	16	53203887	53205824	3'UTR	ENSSSCG00000021902	GABRP	protein_coding	gamma-aminobutyric acid type A receptor pi subunit
16	54212545	54212993	16	54212742	54212890	CDS	ENSSSCG00000017008	DOCK2	protein_coding	dedicator of cytokinesis 2
16	54212545	54212993	16	54212742	54212890	5'UTR	ENSSSCG00000017008	DOCK2	protein_coding	dedicator of cytokinesis 2

16	55132017	55132687	16	55132679	55132822	CDS	ENSSSCG00000017012	SLIT3	protein_coding	slit guidance ligand 3
16	66009643	66010040	16	66009278	66013237	3'UTR	ENSSSCG00000038003	ITK	protein_coding	IL2 inducible T cell kinase
16	66208392	66208872	16	66206286	66208970	3'UTR	ENSSSCG00000017061		protein_coding	hepatitis A virus cellular receptor 1
16	72088168	72088248	16	72088168	72088357	CDS	ENSSSCG00000017094	6-Mar	protein_coding	membrane associated ring-CH-type finger 6
16	75426019	75426159	16	75424872	75429411	3'UTR	ENSSSCG00000035825	UBE2QL1	protein_coding	ubiquitin conjugating enzyme E2 Q family
17	30814	31200	17	30977	31918	CDS	ENSSSCG00000007001		protein_coding	ankyrin repeat domain-containing protein
17	30814	31200	17	30899	31918	CDS	ENSSSCG00000007001		protein_coding	ankyrin repeat domain-containing protein
17	1118492	1118853	17	1116919	1119271	3'UTR	ENSSSCG00000006969	TRMT9B	protein_coding	tRNA methyltransferase 9B (putative)
17	1118492	1118853	17	1116919	1118531	3'UTR	ENSSSCG00000006969	TRMT9B	protein_coding	tRNA methyltransferase 9B (putative)
17	1118492	1118853	17	1116919	1120230	3'UTR	ENSSSCG00000006969	TRMT9B	protein_coding	tRNA methyltransferase 9B (putative)
17	7287845	7288053	17	7287791	7288021	CDS	ENSSSCG00000032493	TRIML1	protein_coding	tripartite motif family like 1
17	9052623	9052907	17	9052454	9052629	CDS	ENSSSCG00000032653	F11	protein_coding	coagulation factor XI
17	9309155	9309898	17	9309490	9309504	CDS	ENSSSCG00000023054	IDO2	protein_coding	indoleamine 2%2C3-dioxygenase 2
17	10777645	10777909	17	10775733	10780592	3'UTR	ENSSSCG00000007022	ANK1	protein_coding	ankyrin 1
17	11016661	11016705	17	11016635	11016842	CDS	ENSSSCG00000007023	KAT6A	protein_coding	lysine acetyltransferase 6A
17	13555359	13555375	17	13553252	13555855	3'UTR	ENSSSCG00000021473		protein_coding	
17	14245081	14245377	17	14242504	14246639	3'UTR	ENSSSCG00000037063	PROKR2	protein_coding	prokineticin receptor 2
17	14778273	14778461	17	14775494	14779239	3'UTR	ENSSSCG00000007046	TRMT6	protein_coding	tRNA methyltransferase 6
17	17488487	17488904	17	17488681	17488818	CDS	ENSSSCG00000007056	PLCB1	protein_coding	phospholipase C beta 1
17	17488487	17488904	17	17488738	17488818	CDS	ENSSSCG00000007056	PLCB1	protein_coding	phospholipase C beta 1
17	17488487	17488904	17	17488677	17488737	5'UTR	ENSSSCG00000007056	PLCB1	protein_coding	phospholipase C beta 1
17	17488487	17488904	17	17488681	17488737	5'UTR	ENSSSCG00000007056	PLCB1	protein_coding	phospholipase C beta 1
17	21902894	21903509	17	21899701	21904399	3'UTR	ENSSSCG00000007072	SPTLC3	protein_coding	serine palmitoyltransferase long chain base subunit 3
17	31079446	31079472	17	31076190	31081633	3'UTR	ENSSSCG00000034913	GINS1	protein_coding	GINS complex subunit 1
17	31931106	31931218	17	31929892	31931712	CDS	ENSSSCT00000044169			
17	32375417	32375740	17	32375566	32375677	CDS	ENSSSCG00000007155	C20orf194	protein_coding	chromosome 17 C20orf194 homolog
17	35539304	35539326	17	35538491	35540971	3'UTR	ENSSSCG00000007232	DUSP15	protein_coding	dual specificity protein phosphatase 15
17	37546928	37547167	17	37547143	37551486	3'UTR	ENSSSCT00000041841			
17	38430972	38431870	17	38431685	38431801	CDS	ENSSSCG00000007289	TRPC4AP	protein_coding	transient receptor potential cation channel subfamily C member 4 associated protein
17	41392818	41393168	17	41391858	41395556	3'UTR	ENSSSCT00000056119			
17	41393195	41393225	17	41391858	41395556	3'UTR	ENSSSCT00000056119			
17	41862626	41862786	17	41862414	41866670	3'UTR	ENSSSCG00000007350	PPP1R16B	protein_coding	protein phosphatase 1 regulatory subunit
17	47525460	47525492	17	47523804	47526750	3'UTR	ENSSSCG00000035053		protein_coding	peptidase inhibitor 3
17	47525460	47525492	17	47524294	47526750	3'UTR	ENSSSCG00000035053		protein_coding	peptidase inhibitor 3
17	47544956	47544980	17	47543788	47546190	3'UTR	ENSSSCG00000035053		protein_coding	peptidase inhibitor 3
17	47544956	47544980	17	47543788	47546190	3'UTR	ENSSSCG00000033675	SPAI	protein_coding	Sus scrofa elafin family member (WAP-3)%2C mRNA.
17	47544956	47544980	17	47543277	47546190	3'UTR	ENSSSCG00000033675	SPAI	protein_coding	Sus scrofa elafin family member (WAP-3)%2C mRNA.
17	47763418	47763430	17	47762382	47765454	3'UTR	ENSSSCG00000035296		protein_coding	pancreatic trypsin inhibitor-like
17	47840735	47841121	17	47840300	47843538	3'UTR	ENSSSCG00000039858	PTI	protein_coding	
17	47840735	47841121	17	47840300	47843994	3'UTR	ENSSSCG00000039858	PTI	protein_coding	
17	50764931	50765429	17	50765134	50765241	CDS	ENSSSCG00000025534	CSE1L	protein_coding	chromosome segregation 1 like
17	56675530	56676349	17	56675990	56676187	CDS	ENSSSCG00000007490	CBLN4	protein_coding	cerebellin 4 precursor
17	56675530	56676349	17	56673724	56675989	3'UTR	ENSSSCG00000007490	CBLN4	protein_coding	cerebellin 4 precursor
17	57032756	57032947	17	57030553	57035130	3'UTR	ENSSSCG00000007495	CASS4	protein_coding	Cas scaffold protein family member 4
18	6142898	6143076	18	6142451	6143449	5'UTR	ENSSSCG00000028266	TMUB1	protein_coding	transmembrane and ubiquitin like domain containing 1
18	6988918	6989096	18	6988884	6993720	3'UTR	ENSSSCG00000035615	GSTK1	protein_coding	glutathione S-transferase kappa 1
18	7433604	7433637	18	7433627	7438705	3'UTR	ENSSSCG00000016475		protein_coding	
18	7433604	7433637	18	7433603	7437870	3'UTR	ENSSSCT00000048057			
18	7798753	7799417	18	7799338	7799466	CDS	ENSSSCG00000016487	MGAM2	protein_coding	maltase-glucoamylase 2 (putative)
18	7807294	7808064	18	7808029	7808184	CDS	ENSSSCG00000016487	MGAM2	protein_coding	maltase-glucoamylase 2 (putative)
18	7871900	7872121	18	7871532	7872376	3'UTR	ENSSSCG00000034639	MGAM	protein_coding	maltase-glucoamylase
18	7871900	7872121	18	7871052	7872376	3'UTR	ENSSSCG00000034639	MGAM	protein_coding	maltase-glucoamylase
18	7920980	7921402	18	7921130	7921186	CDS	ENSSSCG00000034639	MGAM	protein_coding	maltase-glucoamylase
18	7964964	7965573	18	7965405	7965504	5'UTR	ENSSSCG00000034639	MGAM	protein_coding	maltase-glucoamylase
18	7987222	7987724	18	7987390	7987421	CDS	ENSSSCT00000052711			
18	9058853	9059407	18	9059232	9059351	CDS	ENSSSCG00000016494	BRAF	protein_coding	B-Raf proto-oncogene%2C serine/threonine kinase
18	33783286	33783399	18	33783393	33783487	CDS	ENSSSCG00000025602	DOCK4	protein_coding	dedicator of cytokinesis 4
18	42769674	42769760	18	42767384	42771560	3'UTR	ENSSSCG00000037158	MTURN	protein_coding	maturin%2C neural progenitor differentiation regulator homolog
18	42770160	42770189	18	42767384	42771560	3'UTR	ENSSSCG00000037158	MTURN	protein_coding	maturin%2C neural progenitor differentiation regulator homolog
18	43845107	43845288	18	43844933	43846836	3'UTR	ENSSSCG00000016690	CREB5	protein_coding	cAMP responsive element binding protein 5
18	50024678	50025283	18	50024806	50024814	CDS	ENSSSCT00000043780			
18	50086059	50086216	18	50085986	50086141	CDS	ENSSSCG00000016726	ADCY1	protein_coding	adenylate cyclase 1
18	55379667	55379952	18	55379632	55379694	CDS	ENSSSCG00000016772	VPS41	protein_coding	VPS41 subunit of HOPS complex
X	15138551	15138897	X	15138598	15138697	5'UTR	ENSSSCG00000030274	PPEF1	protein_coding	protein phosphatase with EF-hand domain 1
X	28784976	28785544	X	28785459	28785631	CDS	ENSSSCG00000028148	DMD	protein_coding	dystrophin
X	42144586	42144618	X	42142480	42146081	3'UTR	ENSSSCG00000012278	CFP	protein_coding	complement factor properdin
X	43000202	43000537	X	42998782	43002446	3'UTR	ENSSSCG00000021934	OTUD5	protein_coding	OTU deubiquitinase 5
X	46779852	46780127	X	46777923	46782578	3'UTR	ENSSSCG00000035822	FAM120C	protein_coding	family with sequence similarity 120C
X	48706798	48707099	X	48706842	48706981	CDS	ENSSSCG00000012352	KLF8	protein_coding	Kruppel like factor 8
X	50162755	50163228	X	50163028	50163209	CDS	ENSSSCG00000012362	ARHGEF9	protein_coding	Cdc42 guanine nucleotide exchange factor 9
X	50162755	50163228	X	50159926	50163027	3'UTR	ENSSSCG00000012362	ARHGEF9	protein_coding	Cdc42 guanine nucleotide exchange factor 9
X	50162755	50163228	X	50159911	50163027	3'UTR	ENSSSCG00000012362	ARHGEF9	protein_coding	Cdc42 guanine nucleotide exchange factor 9

X	50162755	50163228	X	50163050	50163209	CDS	ENSSSCG00000012362	ARHGEF9	protein_coding	Cdc42 guanine nucleotide exchange factor 9
X	50162755	50163228	X	50159932	50163027	3'UTR	ENSSSCG00000012362	ARHGEF9	protein_coding	Cdc42 guanine nucleotide exchange factor 9
X	50162755	50163228	X	50159925	50163027	3'UTR	ENSSSCG00000012362	ARHGEF9	protein_coding	Cdc42 guanine nucleotide exchange factor 9
X	51162444	51162816	X	51162485	51162657	CDS	ENSSSCG00000027657	ZC4H2	protein_coding	zinc finger C4H2-type containing
X	56774999	56775661	X	56775290	56775368	CDS	ENSSSCG00000030850	TEX11	protein_coding	testis expressed 11
X	57143999	57144224	X	57143895	57144080	CDS	ENSSSCG00000012397		protein_coding	interleukin 2 receptor subunit gamma
X	61650848	61651535	X	61650862	61651015	CDS	ENSSSCG00000012434	ATRX	protein_coding	ATRX chromatin remodeler
X	87286083	87286226	X	87286055	87286152	CDS	ENSSSCG00000022993	TBC1D8B	protein_coding	TBC1 domain family member 8B
X	88052221	88052285	X	88051103	88053273	3'UTR	ENSSSCG00000012561	FRMPD3	protein_coding	FERM and PDZ domain containing 3
X	90307468	90307845	X	90304923	90309334	3'UTR	ENSSSCG00000037591	AMMECR1	protein_coding	Alport syndrome%2C mental retardation%2C midface hypoplasia and elliptocytosis chromosomal region gene 1
X	97532219	97532262	X	97529258	97534185	3'UTR	ENSSSCG00000012631	LONRF3	protein_coding	LON peptidase N-terminal domain and ring finger 3
X	97906298	97906796	X	97905373	97906760	3'UTR	ENSSSCG00000012624	SLC25A43	protein_coding	solute carrier family 25 member 43
X	110947906	110948352	X	110946246	110950993	3'UTR	ENSSSCG00000012691	ZNF449	protein_coding	zinc finger protein 449
X	123845665	123845889	X	123845655	123845737	CDS	ENSSSCG00000012755	GABRQ	protein_coding	gamma-aminobutyric acid type A receptor theta subunit
X	125754551	125754692	X	125754671	125754995	5'UTR	ENSSSCG00000034345	SPRY3	protein_coding	sprouty RTK signaling antagonist 3
Y	4971849	4972075	Y	4968583	4973283	3'UTR	ENSSSCG00000034469		protein_coding	cytokine receptor-like factor 2
Y	39826876	39827069	Y	39826885	39827004	CDS	ENSSSCG00000038249		protein_coding	gamma-taxilin-like

Supplementary Table S3. Annotation results of common SSRs existed among four pig breeds and functional genes affected by SSRs.

8 Summary statistics of alleles detected in 60 pigs

Breed	Sample	chr1: 272,578,714-272,578,954					chr11:70,376,652-70,376,765				chr18:1,858,964-1,859,153				
WZS	W01			230		234			115	117		182			190
	W04					234			115	117				188	
	W06			230		234	109			117		182			190
	W07	224				234	109			117				188	190
	W09					234	109				180				190
	W13					234			115	117				188	190
	W23			230		234			115	117					190
	W24					234	109			117				188	190
	W30	224				234			115	117				188	
	W32	224		230			109		115					188	190
	W35	224				234	109							188	
	W43	224				234	109							188	190
	W44					234				117			186		190
	W46			230		234		113		117				188	190
	W48			230		234			115	117	180			188	
BM	B01			230					115	117					190
	B02	224			232		109			117		182			190
	B03			230			109			117				188	
	B04			230		234			115	117				188	190
	B05	224				234	109			117		182			190
	B06	224		230			109	113				182			190
	B07		228	230			109		115				186		190
	B08			230		234		113			180				190
	B09			230	232		109		115					188	190
	B10					234	109			117			186	188	
	B11			230			109			117				188	190
	B12			230		234			115	117				188	190
	B13			230		234	109		115					188	
	B14			230					115	117				188	190
	B22			230		234	109			117				188	

LC	L01	224								115	117				188	190	
	L02	224					109				117					190	
	L03	224					109				117				188	190	
	L04				234					115	117				188		
	L08	224								115	117				188		
	L09		228				109				117					190	
	L12				234		109				117					190	
	L20	224			234		109				117				188	190	
	L21	224								115	117				188	190	
	L23	224		230					113		117		182		188		
	L36	224					109				117				188		
	L37	224								115	117				188		
	L38	224			234		109				117				188		
	L40	224			234		109				117			186	188		
	L43	224			234		109				117				188	190	
ZX	Z02	224			234			111	113						188		
	Z06		228		234		109		113								212
	Z08		228		234		109			115							212
	Z10	224		230		107	109								188		
	Z13	224		230		107	109						182		188		
	Z17	224	228												188		
	Z22	224			234	107	109								188	190	
	Z24	224			234	107	109						182		188		
	Z28	224		230			109		113				182		188		
	Z29		228				109			115						190	
	Z35			230	234				113		117	180			188		
	Z39				234			111	113			180					212
	Z42	224			234			111	113						188		
	Z45		228		234				113		117	180	182				
	Z50		228	230			109						182		188		

Supplementary Table S4. Alleles of three polymorphic SSRs are detected in 60 pigs. All data were obtained from at least three independent experiments.
